# Supplementary material for: Divergent genomic trajectories predate the origin of animals and fungi
Source: Nature. 2022 Aug 24;609(7928):747–53. doi: 10.1038/s41586-022-05110-4 (PMC9492541; doi:10.1038/s41586-022-05110-4)
Supplement: Supplementary file 1 — Supplementary Figs. 1–3 show a full representation of the boxplots shown in Fig. 1c, a comparison of the ancestral gene content reconstruction analyses using the same dataset as the original analysis as well as the proteomes of Paraphelidium tribonemae and Olpidium bornovanus and original source images for the PCR results shown in Supplementary Information 1 – Fig.3a,b. [file 41586_2022_5110_MOESM1_ESM.pdf]

nature portfolio

https://doi.org/10.1038/s41586-022-05110-4

Supplementary information

Divergent genomic trajectories predate the origin of animals and fungi

In the format provided by the
authors and unedited

Supplementary Figure 1

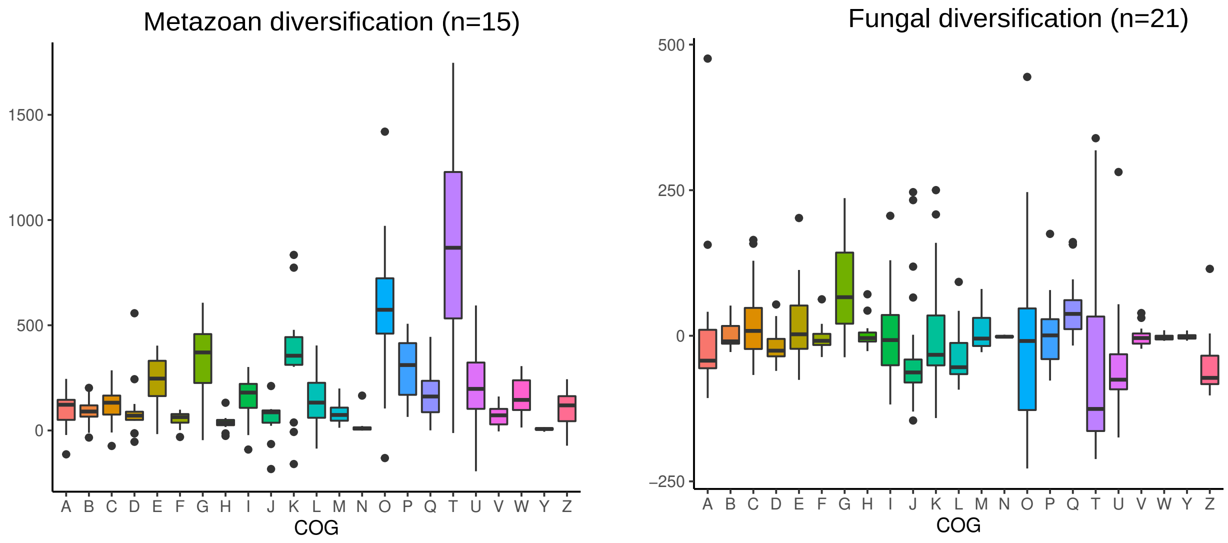

Supplementary Figure 1. Full representation of the boxplots shown in Fig. 1C.

Supplementary Figure 2

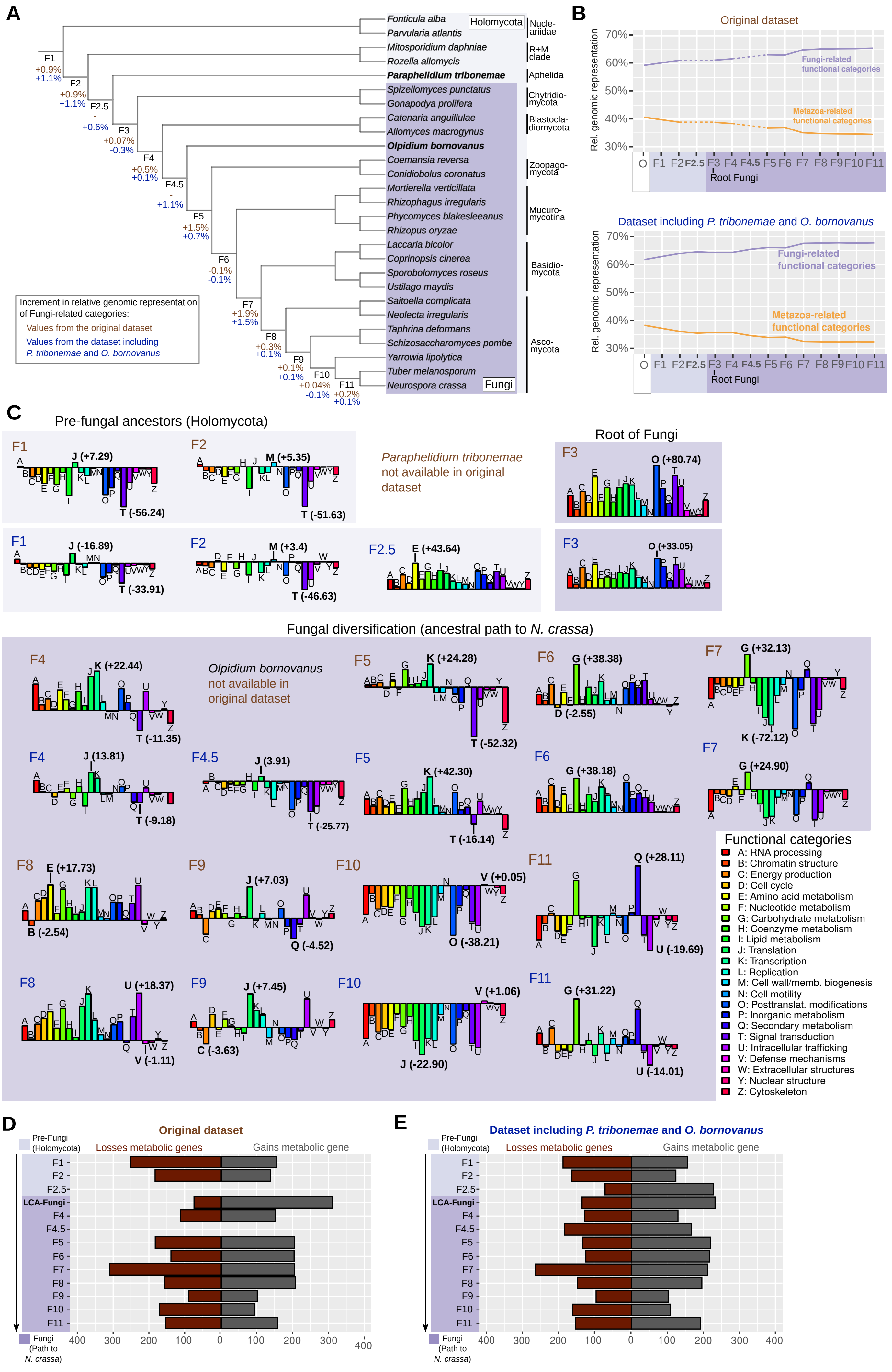

Supplementary Figure 2. Comparison of the ancestral gene content reconstruction analyses using the same dataset as the original analysis as well as the proteomes of *Paraphelidium tribonemae* (transcriptomic data) and *Olpidium bornovanus*. (**A**) Increment in the relative genomic representation of Fungi-related categories in the ancestral path towards *N. crassa* (i) in the original dataset and (ii) in the dataset which also includes *P. tribonemae* (transcriptome) and *O. bornovanus*. (**B**) Similar information to (A) but displayed as line plots. (**C**) Net gains and losses of functional categories from each dataset. Results from the inclusion of *P. tribonemae* suggests that approximately half of net gains detected at the root of Fungi in the original dataset (Fig. 1) could have occurred before the origin of Fungi. (**D-E**) Gains and losses of metabolic genes (KEGG orthology groups) in the ancestral nodes preceding *N. crassa* for the two datasets. Due to computational time limitations, some steps of the original methodological pipeline used to reconstruct ancestral gene contents (see Methods) were not implemented for this second dataset. In particular, prokaryotic homologs were not incoporoated into the orthogroups and the orthogroups were not processed with the MAPBOS pipeline. Overall, the results from the dataset including *P. tribonemae* and *O. bornovanus* dataset confirm the findings from the original dataset due to the fact that all trends of change at the level of genomic composition of functional categories (A-B), net gains and losses of functional categories (C), and net gains and losses of metabolic genes (D) are consistent at node-level between the original and the new dataset; only the ancestral nodes that are located after the positions in which the newly added taxa branch experienced some changes (this pattern is particularly evident in panels C and D).

Supplementary Figure 3

A **B**

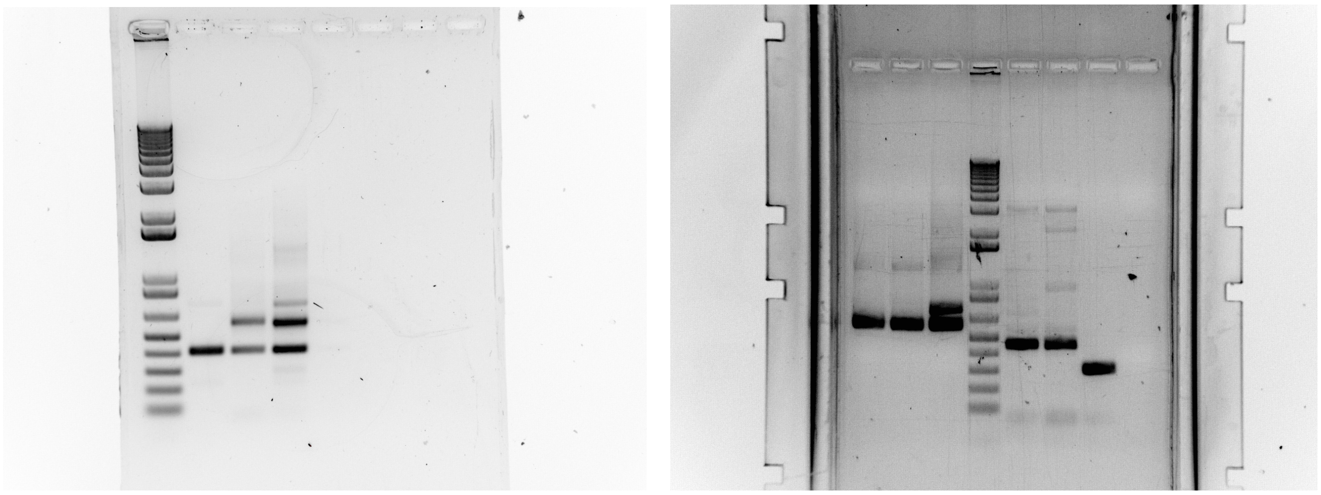

Supplementary Figure 3. (**A**) Original source image for the PCR results shown in Supplementary Information 1-Fig. 3A. In Supplementary Information 1-Fig. 3A, the original source image was cropped to show the lanes corresponding to the DNA ladder (lane 1 counting from the left) and to the two PCR amplifications discussed in Supplementary Information 1-Fig. 3A (lanes 2 and 3). The original source image was also slightly rotated in Supplementary Information 1-Fig. 3A in order to align the lanes with the vertical axis. (**B**) Original source image for the PCR results shown in Supplementary Information 1-Fig. 3B. In Supplementary Information 1-Fig. 3B, the original source image was cropped to show the lanes corresponding to the DNA ladder and to the PCR amplifications discussed in Supplementary Information 1-Fig. 3B.
