# Supplementary material for: Divergent genomic trajectories predate the origin of animals and fungi
Source: Nature. 2022 Aug 24;609(7928):747–53. doi: 10.1038/s41586-022-05110-4 (PMC9492541; doi:10.1038/s41586-022-05110-4)
Supplement: Supplementary file 2 — Reporting Summary [file 41586_2022_5110_MOESM2_ESM.pdf]

nature portfolio

Corresponding author(s): IÑAKI RUIZ-TRILLO, EDUARD OCAÑA-PALLARES

Last updated by author(s): May 24, 2022

Reporting Summary

Nature Portfolio wishes to improve the reproducibility of the work that we publish. This form provides structure for consistency and transparency in reporting. For further information on Nature Portfolio policies, see our Editorial Policies and the Editorial Policy Checklist.

Statistics

For all statistical analyses, confirm that the following items are present in the figure legend, table legend, main text, or Methods section.

n/a	Confirmed	
☐	☒	The exact sample size (*n*) for each experimental group/condition, given as a discrete number and unit of measurement
☒	☐	A statement on whether measurements were taken from distinct samples or whether the same sample was measured repeatedly
☐	☒	The statistical test(s) used AND whether they are one- or two-sided<br>*Only common tests should be described solely by name; describe more complex techniques in the Methods section.*
☒	☐	A description of all covariates tested
☒	☐	A description of any assumptions or corrections, such as tests of normality and adjustment for multiple comparisons
☐	☒	A full description of the statistical parameters including central tendency (e.g. means) or other basic estimates (e.g. regression coefficient) AND variation (e.g. standard deviation) or associated estimates of uncertainty (e.g. confidence intervals)
☐	☒	For null hypothesis testing, the test statistic (e.g. *F*, *t*, *r*) with confidence intervals, effect sizes, degrees of freedom and *P* value noted<br>*Give P values as exact values whenever suitable.*
☐	☒	For Bayesian analysis, information on the choice of priors and Markov chain Monte Carlo settings
☒	☐	For hierarchical and complex designs, identification of the appropriate level for tests and full reporting of outcomes
☒	☐	Estimates of effect sizes (e.g. Cohen's *d*, Pearson's *r*), indicating how they were calculated

Our web collection on statistics for biologists contains articles on many of the points above.

Software and code

Policy information about availability of computer code

Data collection	Genomic data was manually collected from online repositories, no software was used for this purpose.
Data analysis	Custom algorithms and scripts that are central to the research, all written in Python (3.6.4), are described in Material and methods and Supplementary Information. Only open source software was used for data analysis: BRAKER1 v1.9, PASA v2.0.2, BLASTp v2.5, OrthoFinder v2.7, MAFFT v7.123b, trimAl v1.4.rev15, IQ-TREE v1.6.7, PhyloBayes-MPI v1.8, DIAMOND v0.8.22.84, ALE v0.4, CompositeSearch v1.0, PfamScan.pl v1.0, eggNOG-mapper v1.0.3-3-g3e2272, trimmomatic v0.36, FastQC v0.11.5, nxtrim v0.4.1, SPAdes v3.10.1, GeneMark-ES/ET v4.33, AUGUSTUS v3.1.0, SEECER v0.1.3, TopHat v2.1.1, Databionics ESOM Tools v1.0, bowtie2 v2.2.9, CD-HIT v4.6, CONCOCT v0.4.1, IDBA-UD v1.1.1, Ray Meta v.2.3.1, BUSCO v1.22, QUAST v4.2, samtools 1.3.1, PASA v2.0.2, blat v35x1, GMAP v2015-12-31, RepeatMasker version open-4.0.6, RepeatModeler v1.0.4, TransDecoder.LongOrfs v3.0.1, Trinity v2.2.0, pandas v1.0.5, Keras v2.4.3, scikit-learn v0.23.1, R v3.6.3, numpy v1.18.5.<br><br>The custom code developed in this study is available at https://doi.org/10.5281/zenodo.6586559

For manuscripts utilizing custom algorithms or software that are central to the research but not yet described in published literature, software must be made available to editors and reviewers. We strongly encourage code deposition in a community repository (e.g. GitHub). See the Nature Portfolio guidelines for submitting code & software for further information.

Data

Policy information about availability of data

All manuscripts must include a data availability statement. This statement should provide the following information, where applicable:

- Accession codes, unique identifiers, or web links for publicly available datasets
- A description of any restrictions on data availability
- For clinical datasets or third party data, please ensure that the statement adheres to our policy

The raw sequence data and assembled genomes generated in this study have been deposited at the European Nucleotide Archive (project accession code PRJEB52884). The genome assemblies have also been deposited at Figshare (https://doi.org/10.6084/m9.figshare.19895962.v1). Protein sequences of the species used in this study were downloaded from the GenBank public databases (https://www.ncbi.nlm.nih.gov/protein/), Uniprot (https://www.uniprot.org/), JGI genome database (https://genome.jgi.doe.gov/portal/) and Ensembl genomes (https://www.ensembl.org). The following specific databases were also used in this study: Pfam A v29 (https://pfam.xfam.org/), EggNOG emapperdb-4.5.1 (http://eggnog5.embl.de) and UniProt reference proteomes Release 2016_02 (https://www.uniprot.org/). The supporting data files of this study are available in Figshare (https://doi.org/10.6084/m9.figshare.13140191.v1).

Field-specific reporting

Please select the one below that is the best fit for your research. If you are not sure, read the appropriate sections before making your selection.

☐ Life sciences ☐ Behavioural & social sciences ☒ Ecological, evolutionary & environmental sciences

For a reference copy of the document with all sections, see nature.com/documents/nr-reporting-summary-flat.pdf

Life sciences study design

All studies must disclose on these points even when the disclosure is negative.

Sample size	*Describe how sample size was determined, detailing any statistical methods used to predetermine sample size OR if no sample-size calculation was performed, describe how sample sizes were chosen and provide a rationale for why these sample sizes are sufficient.*
Data exclusions	*Describe any data exclusions. If no data were excluded from the analyses, state so OR if data were excluded, describe the exclusions and the rationale behind them, indicating whether exclusion criteria were pre-established.*
Replication	*Describe the measures taken to verify the reproducibility of the experimental findings. If all attempts at replication were successful, confirm this OR if there are any findings that were not replicated or cannot be reproduced, note this and describe why.*
Randomization	*Describe how samples/organisms/participants were allocated into experimental groups. If allocation was not random, describe how covariates were controlled OR if this is not relevant to your study, explain why.*
Blinding	*Describe whether the investigators were blinded to group allocation during data collection and/or analysis. If blinding was not possible, describe why OR explain why blinding was not relevant to your study.*

Behavioural & social sciences study design

All studies must disclose on these points even when the disclosure is negative.

Study description	*Briefly describe the study type including whether data are quantitative, qualitative, or mixed-methods (e.g. qualitative cross-sectional, quantitative experimental, mixed-methods case study).*
Research sample	*State the research sample (e.g. Harvard university undergraduates, villagers in rural India) and provide relevant demographic information (e.g. age, sex) and indicate whether the sample is representative. Provide a rationale for the study sample chosen. For studies involving existing datasets, please describe the dataset and source.*
Sampling strategy	*Describe the sampling procedure (e.g. random, snowball, stratified, convenience). Describe the statistical methods that were used to predetermine sample size OR if no sample-size calculation was performed, describe how sample sizes were chosen and provide a rationale for why these sample sizes are sufficient. For qualitative data, please indicate whether data saturation was considered, and what criteria were used to decide that no further sampling was needed.*
Data collection	*Provide details about the data collection procedure, including the instruments or devices used to record the data (e.g. pen and paper, computer, eye tracker, video or audio equipment) whether anyone was present besides the participant(s) and the researcher, and whether the researcher was blind to experimental condition and/or the study hypothesis during data collection.*
Timing	*Indicate the start and stop dates of data collection. If there is a gap between collection periods, state the dates for each sample cohort.*
Data exclusions	*If no data were excluded from the analyses, state so OR if data were excluded, provide the exact number of exclusions and the*

Data exclusions

rationale behind them, indicating whether exclusion criteria were pre-established.

Non-participation

State how many participants dropped out/declined participation and the reason(s) given OR provide response rate OR state that no participants dropped out/declined participation.

Randomization

If participants were not allocated into experimental groups, state so OR describe how participants were allocated to groups, and if allocation was not random, describe how covariates were controlled.

Ecological, evolutionary & environmental sciences study design

All studies must disclose on these points even when the disclosure is negative.

Study description

We have reconstructed the tempo and mode of the genomic divergence process that accompanied the origin of animals and fungi since the divergence of the last shared common ancestor of both groups (Opisthokonta). For it, we first analysed in a comparative manner the gene contents of modern animals and fungi in order the identify the fundamental genomic differences between them. Then, based on the gene content of extant representatives of both groups as well as from other opisthokont lineages that branch between Metazoa and Fungi, we used a phylogenetic approach (a gene-tree/species-tree reconciliation software) to reconstruct the ancestral gene contents and the genetic turnover occurred at every ancestral lineage in the Opisthokonta phylogeny. This methodological workflow allowed us to compare the genetic changes that occurred in the evolutionary path towards both groups at the level of (i) gene gains and losses, (ii) functional specialization of the gene content, and (iii) between-group differences in the relative preference for a series of mechanisms that can operate as sources of new genes.

Research sample

This study re-analyses mostly publicly available protein sequence predictions of species gene contents (hereafter referred to as gene content) from genomic or transcriptomic data, as well as the gene contents annotated from four newly sequenced protist opisthokont species in this mansucript: Parvularia atlantis, Ministeria vibrans, Pigoraptor vietnamica and Pigoraptor chileana. In particular, the dataset included the gene contents from 16 metazoans (Holozoa, Opisthokonta), 18 non-metazoan Holozoa, 21 Fungi (Holomycota, Opisthokonta), 4 non-fungal Holomycota, and 24 non-opisthokont eukaryotes. The species included in the dataset were chosen in order to maximize the taxonomic representation of the distinct metazoan and fungal groups, while also from the distinct opisthokont lineages that branch between animals and fungi. Gene content data from other eukaryotic groups were also included in order to ensure that the reconstructed gene trees could include phylogenetic information also from the evolutionary history preceding the origin of the specific group of interest for this study (Opstihokonta). Sequences from prokaryotes and virsuses were also added into the dataset for this same purpose, although they were incorporated after the clustering of the sampled eukaryotic sequences into orthogroups (see Methods section in the manuscript for a detailed explanation). Prokaryotic and viral sequences were incorporated from a database that included 8231104, 331476 and 20955 sequences from Bacteria, Archaea and viruses, respetively, which correspond to all Uniprot reference proteomes from these groups (release 2016_02). The final dataset (euk_db, see Methods section in the manuscript for details) that was used for the ancestral reconstruction analysis of gene content consisted of 1117614 eukaryotic sequences and 58017 non-eukaryotic sequences.

Sampling strategy

Sampling strategy: The dataset size was constrainted by the availability of genomic data and by some computational analyses. In particular, there were some methodological steps that were more computationally demanding than others, and for which a reduced version (euk_db) of the original dataset (draft_euk_db) was used (see Supplementary Table 4 for a description of the eukaryotic groups included in each dataset, and Methods section for an explanation of which dataset was used in every methodological step). The draft_euk_db dataset included gene content data from 83 eukaryotic species that represent all major eukaryotic groups. According to the interest of this study, the taxonomic representation of the eukaryotic supergroup Opisthokonta was prioritized (59 species). Only 9 opisthokont species were not included in the reduced euk_db dataset, 8 of them because gene content annotations came from transcriptomic data, and one species (Oscarella carmela) because its reduced gene content size was suggestive of incomplete genomic data or of this being an outlier species with a highly reduced genome. A total of 15 species were from draft_euk_db were not included in euk_db.

The size of the taxon sampling used in this study (83 genomes) is in the same scale than the taxon samplings used in similar studies (e.g., 69 genomes in https://doi.org/10.7554/eLife.26036.005, 72 genomes in https://doi.org/10.1038/s41467-019-12085-w), with the advantage that we have incorporated four novel species genomes that we sequenced for two taxonomic groups that were previously represented by only one species genome each (Filasterea and Nucleariidea). This allowed us to use the most updated possible taxon sampling at genome level with regard to the taxonomic groups that are phylogenetically related to animals and to fungi (among which Filasterea and Nucleariidea are included, see Fig. 1). In phylogenetic analyses, statistical robustness is estimated using bootstrap support values or, in Bayesian analyses, posterior probabilities; low support values might indicate a lack of statistical power to distinguish between hypotheses of relationship. The uncertainty associated with our estimates is provided using these standard metrics. The high support values in the reconstructed phylogenies (see Supplementary Information 3-Fig. 1A,B) indicate that sampling was sufficient.

Data collection

The first author of the manuscript downloaded the genomic data from public genomic repositories (see Data availability statement). Data downloading procedure was recorded in an Excel file and is shown in Supplementary Table 4.

Timing and spatial scale

The genomic data were downloaded from 2016 to 2018 from the public genomic repositories as indicated in the 'Data collection' section, and more into detail in the Methods section.

Data exclusions

Transcriptomic data was only used for species tree reconstruction, in particular for those taxa with phylogenetically relevant positions in the context of the Opisthoknota phylogeny but for which genomic data is not yet available. Transcriptomic data was not used in the ancestral gene content reconstruction analyis because the gene contents that are obtained from transcriptome tend to be more inaccurate than those that are obtained when the genome sequence is also available. For example, uncollapsed transcriptome isoforms that may have been annotated as separate gene sequences can lead the reconciliation software to infer false gene duplications, unexpressed genes can be confused with gene losses, sequence contamination -which is harder to detect in

	transcriptomic data- can be confused with horizontal gene transfers, and partial fragments can lead to artifactual protein domain architectures which can confuse the algorithm used to detect gene fusion events.
Reproducibility	The raw genomic data, software, software versions and software parameters are specified in the Methods section, to enable all analyses to be reproduced or built upon as needed. Note that all analyses are done with bioinformatic methods and hence the reproducibility of the results is guaranteed as long as the same data, software versions and software parameters are used.
Randomization	The paper reports phylogenetic and comparative genomic analyses. As is standard for the field, the robustness of inferences was assessed using bootstrap support values and Bayesian posterior probabilities.
Blinding	Blinding was not relevant to the study design, because this was a phylogenetic and comparative genomic analysis of all of the available data (that is, the experimental design did not involve allocating data to groups).

Did the study involve field work? ☐ Yes ☒ No

Field work, collection and transport

Field conditions	*Describe the study conditions for field work, providing relevant parameters (e.g. temperature, rainfall).*
Location	*State the location of the sampling or experiment, providing relevant parameters (e.g. latitude and longitude, elevation, water depth).*
Access & import/export	*Describe the efforts you have made to access habitats and to collect and import/export your samples in a responsible manner and in compliance with local, national and international laws, noting any permits that were obtained (give the name of the issuing authority, the date of issue, and any identifying information).*
Disturbance	*Describe any disturbance caused by the study and how it was minimized.*

Reporting for specific materials, systems and methods

We require information from authors about some types of materials, experimental systems and methods used in many studies. Here, indicate whether each material, system or method listed is relevant to your study. If you are not sure if a list item applies to your research, read the appropriate section before selecting a response.

Materials & experimental systems

n/a	Involved in the study	
☒	☐	Antibodies
☐	☒	Eukaryotic cell lines
☒	☐	Palaeontology and archaeology
☒	☐	Animals and other organisms
☒	☐	Human research participants
☒	☐	Clinical data
☒	☐	Dual use research of concern

Methods

n/a	Involved in the study	
☒	☐	ChIP-seq
☒	☐	Flow cytometry
☒	☐	MRI-based neuroimaging

Antibodies

Antibodies used	*Describe all antibodies used in the study; as applicable, provide supplier name, catalog number, clone name, and lot number.*
Validation	*Describe the validation of each primary antibody for the species and application, noting any validation statements on the manufacturer's website, relevant citations, antibody profiles in online databases, or data provided in the manuscript.*

Eukaryotic cell lines

Policy information about cell lines

Cell line source(s)	Ministeria vibrans' culture was bought in ATCC (Ministeria vibrans Tong. ATCC 50519). Parvularia atlantis' (formerly Nuclearia sp.) culture was bought in ATCC (Nuclearia sp. ATCC 50694). The cultures of Pigoraptor vietnamica (formerly Opistho-1) and Pigoraptor chileana (formerly Opistho-2) descend from the environmental isolates (P. vietnamica from a Freshwater Lake, Vietnam, and P. chileana from freshwater temporary water body, Chile) used in the manuscript https://doi.org/10.1016/j.cub.2017.06.006 (see 'METHOD DETAILS' section).
Authentication	The presence of our organisms of interest in the sequenced cell lines was validated through microscopy observation and genetic analyses before genomic sequencing. The genes from the organisms of interests are found in the sequenced data, confirming their presence in the sequenced cell lines.
Mycoplasma contamination	The culture lines used in this study are not pure cell lines, and hence include other species besides our organisms of interest. For this reason, the genomic data produced was fully decontaminated by means of a comprehensive bioinformatic analyses consisting of distinct iterative rounds of decontamination, as described in Supplementary Information.

Commonly misidentified lines (See ICLAC register)	*Name any commonly misidentified cell lines used in the study and provide a rationale for their use.*

Palaeontology and Archaeology

Specimen provenance	*Provide provenance information for specimens and describe permits that were obtained for the work (including the name of the issuing authority, the date of issue, and any identifying information). Permits should encompass collection and, where applicable, export.*
Specimen deposition	*Indicate where the specimens have been deposited to permit free access by other researchers.*
Dating methods	*If new dates are provided, describe how they were obtained (e.g. collection, storage, sample pretreatment and measurement), where they were obtained (i.e. lab name), the calibration program and the protocol for quality assurance OR state that no new dates are provided.*

☐ Tick this box to confirm that the raw and calibrated dates are available in the paper or in Supplementary Information.

Ethics oversight	*Identify the organization(s) that approved or provided guidance on the study protocol, OR state that no ethical approval or guidance was required and explain why not.*

Note that full information on the approval of the study protocol must also be provided in the manuscript.

Animals and other organisms

Policy information about studies involving animals; ARRIVE guidelines recommended for reporting animal research

Laboratory animals	*For laboratory animals, report species, strain, sex and age OR state that the study did not involve laboratory animals.*
Wild animals	*Provide details on animals observed in or captured in the field; report species, sex and age where possible. Describe how animals were caught and transported and what happened to captive animals after the study (if killed, explain why and describe method; if released, say where and when) OR state that the study did not involve wild animals.*
Field-collected samples	*For laboratory work with field-collected samples, describe all relevant parameters such as housing, maintenance, temperature, photoperiod and end-of-experiment protocol OR state that the study did not involve samples collected from the field.*
Ethics oversight	*Identify the organization(s) that approved or provided guidance on the study protocol, OR state that no ethical approval or guidance was required and explain why not.*

Note that full information on the approval of the study protocol must also be provided in the manuscript.

Human research participants

Policy information about studies involving human research participants

Population characteristics	*Describe the covariate-relevant population characteristics of the human research participants (e.g. age, gender, genotypic information, past and current diagnosis and treatment categories). If you filled out the behavioural & social sciences study design questions and have nothing to add here, write "See above."*
Recruitment	*Describe how participants were recruited. Outline any potential self-selection bias or other biases that may be present and how these are likely to impact results.*
Ethics oversight	*Identify the organization(s) that approved the study protocol.*

Note that full information on the approval of the study protocol must also be provided in the manuscript.

Clinical data

Policy information about clinical studies

All manuscripts should comply with the ICMJE guidelines for publication of clinical research and a completed CONSORT checklist must be included with all submissions.

Clinical trial registration	*Provide the trial registration number from ClinicalTrials.gov or an equivalent agency.*
Study protocol	*Note where the full trial protocol can be accessed OR if not available, explain why.*
Data collection	*Describe the settings and locales of data collection, noting the time periods of recruitment and data collection.*
Outcomes	*Describe how you pre-defined primary and secondary outcome measures and how you assessed these measures.*

Dual use research of concern

Policy information about [dual use research of concern]

Hazards

Could the accidental, deliberate or reckless misuse of agents or technologies generated in the work, or the application of information presented in the manuscript, pose a threat to:

No	Yes	
☐	☐	Public health
☐	☐	National security
☐	☐	Crops and/or livestock
☐	☐	Ecosystems
☐	☐	Any other significant area

Experiments of concern

Does the work involve any of these experiments of concern:

No	Yes	
☐	☐	Demonstrate how to render a vaccine ineffective
☐	☐	Confer resistance to therapeutically useful antibiotics or antiviral agents
☐	☐	Enhance the virulence of a pathogen or render a nonpathogen virulent
☐	☐	Increase transmissibility of a pathogen
☐	☐	Alter the host range of a pathogen
☐	☐	Enable evasion of diagnostic/detection modalities
☐	☐	Enable the weaponization of a biological agent or toxin
☐	☐	Any other potentially harmful combination of experiments and agents

ChIP-seq

Data deposition

☐ Confirm that both raw and final processed data have been deposited in a public database such as GEO.

☐ Confirm that you have deposited or provided access to graph files (e.g. BED files) for the called peaks.

Data access links<br>*May remain private before publication.*	*For "Initial submission" or "Revised version" documents, provide reviewer access links. For your "Final submission" document, provide a link to the deposited data.*
Files in database submission	*Provide a list of all files available in the database submission.*
Genome browser session<br>(e.g. UCSC)	*Provide a link to an anonymized genome browser session for "Initial submission" and "Revised version" documents only, to enable peer review. Write "no longer applicable" for "Final submission" documents.*

Methodology

Replicates	*Describe the experimental replicates, specifying number, type and replicate agreement.*
Sequencing depth	*Describe the sequencing depth for each experiment, providing the total number of reads, uniquely mapped reads, length of reads and whether they were paired- or single-end.*
Antibodies	*Describe the antibodies used for the ChIP-seq experiments; as applicable, provide supplier name, catalog number, clone name, and lot number.*
Peak calling parameters	*Specify the command line program and parameters used for read mapping and peak calling, including the ChIP, control and index files used.*
Data quality	*Describe the methods used to ensure data quality in full detail, including how many peaks are at FDR 5% and above 5-fold enrichment.*
Software	*Describe the software used to collect and analyze the ChIP-seq data. For custom code that has been deposited into a community repository, provide accession details.*

Flow Cytometry

Plots

Confirm that:

☒ The axis labels state the marker and fluorochrome used (e.g. CD4-FITC).

☒ The axis scales are clearly visible. Include numbers along axes only for bottom left plot of group (a 'group' is an analysis of identical markers).

☒ All plots are contour plots with outliers or pseudocolor plots.

☒ A numerical value for number of cells or percentage (with statistics) is provided.

Methodology

Sample preparation	Sorted cells were sampled from polyxenic protist cultures including the eukaryotic species of interest as well as an uncertain fraction of bacterial contamination
Instrument	BD FACSAria II cell sorter (Becton Dickinson, San Jose, CA). Model number: P5X10001
Software	Facsdiva Software Version 6.1.2
Cell population abundance	The final population sorted represented less than 1% of the total cells in the sample. The aim was to enrich the population of eukaryotic cells and to minimize the fraction of contaminantion in the sequenced metagenomic data. As expected, some contamination remained in the sequenced pool of sorted cells which was subsequently eliminated with a comprehensive bioinformatic pipeline that is thoroughtly explained in Supplementary Information 1.
Gating strategy	We used Forward Scatter (FSC) and Side Scatter (SCC) lasers together with the green fluorescence (FITC channel 525/50 nm) to target larger and complex eukaryotic cells that incorporated Lysotracker-green DND-26, which is eukaryotic specific. Next, we could discriminate which eukaryotic cells had a larger fraction of bacterial cells attached with the dye 5-Cyano-2,3-ditolyl tetrazolium chloride (CTC, PerCPCy5.5 channel 685/35 nm). We sorted the population of eukaryotic cells that presented the lowest CTC signal in order to minimize the fraction of bacterial contamination in the sorted cells.

☒ Tick this box to confirm that a figure exemplifying the gating strategy is provided in the Supplementary Information.

Magnetic resonance imaging

Experimental design

Design type	*Indicate task or resting state; event-related or block design.*
Design specifications	*Specify the number of blocks, trials or experimental units per session and/or subject, and specify the length of each trial or block (if trials are blocked) and interval between trials.*
Behavioral performance measures	*State number and/or type of variables recorded (e.g. correct button press, response time) and what statistics were used to establish that the subjects were performing the task as expected (e.g. mean, range, and/or standard deviation across subjects).*

Acquisition

Imaging type(s)	*Specify: functional, structural, diffusion, perfusion.*
Field strength	*Specify in Tesla*
Sequence & imaging parameters	*Specify the pulse sequence type (gradient echo, spin echo, etc.), imaging type (EPI, spiral, etc.), field of view, matrix size, slice thickness, orientation and TE/TR/flip angle.*
Area of acquisition	*State whether a whole brain scan was used OR define the area of acquisition, describing how the region was determined.*

Diffusion MRI ☐ Used ☐ Not used

Preprocessing

Preprocessing software	*Provide detail on software version and revision number and on specific parameters (model/functions, brain extraction, segmentation, smoothing kernel size, etc.).*
Normalization	*If data were normalized/standardized, describe the approach(es): specify linear or non-linear and define image types used for transformation OR indicate that data were not normalized and explain rationale for lack of normalization.*
Normalization template	*Describe the template used for normalization/transformation, specifying subject space or group standardized space (e.g.*

Normalization template | *original Talairach, MNI305, ICBM152) OR indicate that the data were not normalized.*

Noise and artifact removal | *Describe your procedure(s) for artifact and structured noise removal, specifying motion parameters, tissue signals and physiological signals (heart rate, respiration).*

Volume censoring | *Define your software and/or method and criteria for volume censoring, and state the extent of such censoring.*

Statistical modeling & inference

Model type and settings | *Specify type (mass univariate, multivariate, RSA, predictive, etc.) and describe essential details of the model at the first and second levels (e.g. fixed, random or mixed effects; drift or auto-correlation).*

Effect(s) tested | *Define precise effect in terms of the task or stimulus conditions instead of psychological concepts and indicate whether ANOVA or factorial designs were used.*

Specify type of analysis: ☐ Whole brain ☐ ROI-based ☐ Both

Statistic type for inference (See Eklund et al. 2016) | *Specify voxel-wise or cluster-wise and report all relevant parameters for cluster-wise methods.*

Correction | *Describe the type of correction and how it is obtained for multiple comparisons (e.g. FWE, FDR, permutation or Monte Carlo).*

Models & analysis

n/a	Involved in the study
☐	☐ Functional and/or effective connectivity
☐	☐ Graph analysis
☐	☐ Multivariate modeling or predictive analysis

Functional and/or effective connectivity | *Report the measures of dependence used and the model details (e.g. Pearson correlation, partial correlation, mutual information).*

Graph analysis | *Report the dependent variable and connectivity measure, specifying weighted graph or binarized graph, subject- or group-level, and the global and/or node summaries used (e.g. clustering coefficient, efficiency, etc.).*

Multivariate modeling and predictive analysis | *Specify independent variables, features extraction and dimension reduction, model, training and evaluation metrics.*
