# Supplementary material for: Divergent genomic trajectories predate the origin of animals and fungi
Source: Nature. 2022 Aug 24;609(7928):747–53. doi: 10.1038/s41586-022-05110-4 (PMC9492541; doi:10.1038/s41586-022-05110-4)
Supplement: Supplementary file 3 — Detailed explanation of the methodological pipeline followed to produce genomic data for Ministeria vibrans, Parvularia atlantis, Pigoraptor vietnamica and Pigoraptor chileana. [file 41586_2022_5110_MOESM3_ESM.pdf]

Supplementary Information 1

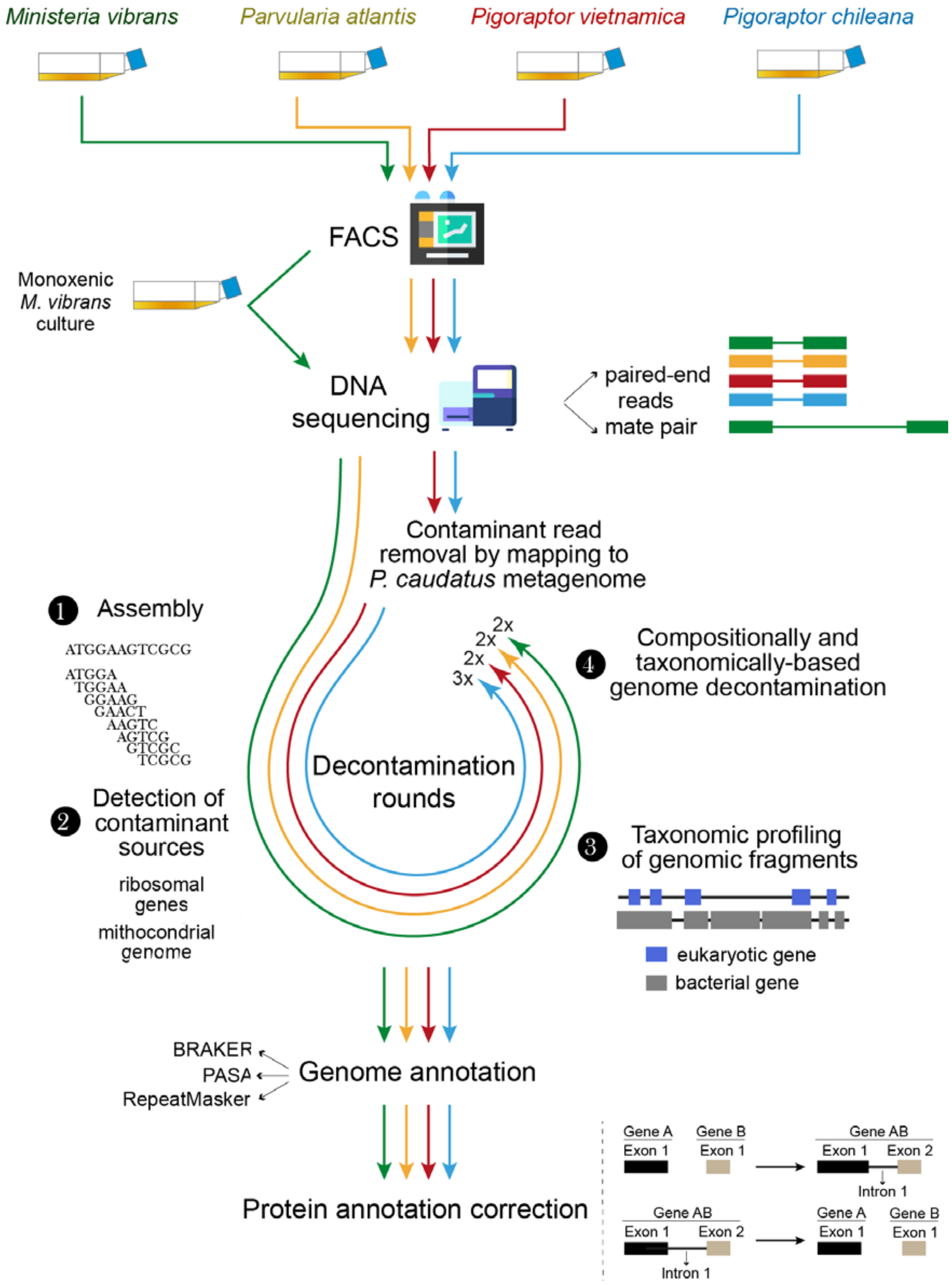

Supplementary Information 1-Fig. 1. Schematic representation of the methodological approach followed for the acquisition of genomic data for these four unicellular opisthokonts from polyxenic cultures. Icons made by Freepik from www.flaticon.com.

1) *Ministeria vibrans* (Filasterea, Opisthokonta)

1.1) Cultures, Cell cytometry and DNA sequencing

We started from non-axenic cultures of *M. vibrans* ATCC 50519[1] growing in ATCC Medium 1525 and maintained at 23 ºC. Fluorescence-activated cell sorting (FACS) was used to isolate *M. vibrans* in a rich medium supplemented with antibiotics and with chemically killed bacteria (see below), also maintained at 23 ºC. Flow cytometry analyses and cell sorting of the cultures were performed in a BD FACSAria II cell sorter (Becton Dickinson, San Jose, CA) equipped with 488 argon laser. For that, samples were incubated with 5-cyao-2,3-diotolyl tetrazolium chloride and LysoTracker Green DND-26 to differentially label bacterial and eukaryotic cells, respectively. We used the gating strategy based in the following sequence (Supplementary Information 1-Fig. 2): Forward Scatter (FSC) vs. green fluorescence (FITC channel 525/50 nm band-pass filter, Lysotracker-green Fluorescence); and FSC versus Side Scatter (SCC). From these plots, we defined a population including those larger and green fluorescent cells (P2, Supplementary Information 1-Fig. 2A-B). That population was subsequently gated for red (PerCPCy5.5 channel 685/35 nm band-pass filter) versus green dot-plot (PerCP-5.5 vs. FITC), to finally sort only green cells (P4, Supplementary Information 1-Fig. 2C) corresponding to the eukaryotic cells. Sorted cells were collected in 48-well plates filled with rich medium. The rich medium was prepared by mixing two-thirds of ATCC® 327-X™ with one-third of Phosphate-buffered saline and adding 3.6g/100mL of salts, being sterilized with filters of 0.22 µM. Cultures of *M. vibrans* growing in the rich medium were supplemented with Gentamicin and Chloramphenicol to maintain them free of potential bacterial contamination. They were also fed with chemically killed *Enterobacter aerogenes* samples that were prepared by treating them overnight with 0.5% of formaldehyde and then washed two times with PBS.

We used a duplex PCR-based assay for measuring the ratio of 18S/16S ribosomal genes[2]. Results indicated a considerable improvement of the *M. vibrans*/Bacteria ratio in the new cultures compared to the original cultures (Supplementary Information 1-Fig. 3A).

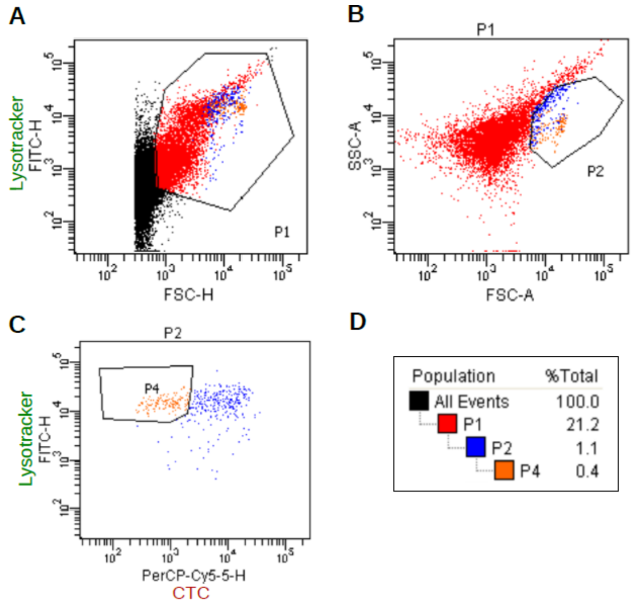

Supplementary Information 1-Fig. 2. Plots showing the strategy to sorter *M. vibrans* cells using fluorescence-activated cell sorting. Samples were incubated with 5-cyao-2,3-diotolyl tetrazolium chloride (CTC) and LysoTracker Green DND-26 (LysoTracker) to differentially label bacterial and eukaryotic cells, respectively. Flow cytometry analyses and cell sorting of the cultures were performed in a BD FACSAria II cell sorter (Becton Dickinson, San Jose, CA) equipped with 488 argon laser. We used the gating strategy based in the following sequence: (**A**) Forward Scatter (FSC) vs. green fluorescence (FITC channel 525/50 nm band-pass filter, Lysotracker green Fluorescence); and (**B**) FSC vs Side Scatter (SCC). From these plots, we defined a population including those larger and green fluorescent cells (P2). (**C**) That population was subsequently gated for red (PerCPCy5.5 channel 685/35 nm band-pass filter) vs green dot-plot (PerCP-5.5 vs. FITC), to finally sort only orange cells (P4) corresponding to the eukaryotic cells. Sorted cells were collected in 48-well plates. (**D**) Percentage of events corresponding to each defined population.

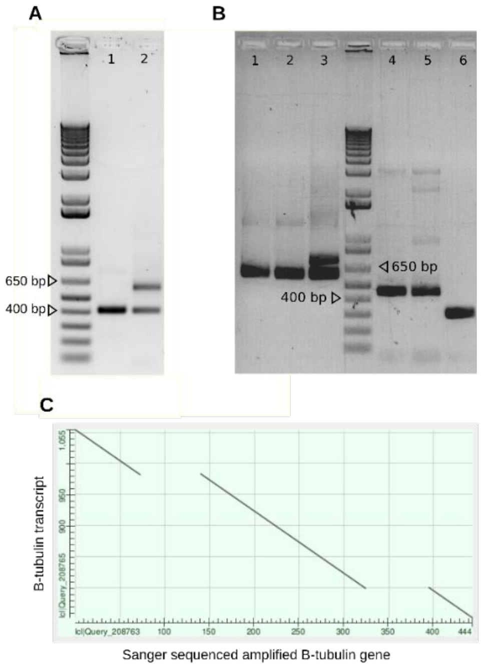

Supplementary Information 1-Fig. 3 (**A**) Agarose gel showing the amplification products of the duplex PCR-based assay for measuring the ratio of 18S/16S ribosomal genes[2]. Lanes 1 and 2 correspond to DNA extractions from the new and the original cultures of *M. vibrans*, respectively. The expected band length for the amplified 18S and 16S products are 400 and 600 bp, respectively. The intensity of the bands indicate that the new cultures show better 18S/16S ratios than the old cultures. For gel source data, see Supplementary Figure 3A. (**B**) Agarose gel showing the amplification products of two PCRs: (i) using 16S universal primers (lanes 1-3) and (ii) using specific primers for the *P. atlantis* B-tubulin gene (lanes 4-6). Lanes 1, 2, 4, 5 correspond to DNA extractions from the pooling of cells sorted by flow cytometry analyses of *P. atlantis* cultures; whereas lanes 3 and 6 correspond to cDNA from *P. atlantis* cultures. The cDNA from *P. atlantis* was obtained using the protocol described in [3]. The B-tubulin was used as a marker for the presence of *P. atlantis* genomic DNA instead of the 18S because we were not able to amplify this ribosomal gene from previous genomic DNA extractions. The amplified bands suggest the presence of both 16S and B-tubulin genes in the DNA extractions from the pooling of sorted cells, suggesting the presence of our organism of interest but also of uncertain bacterial contamination. We confirmed that the bands in the lanes 4 and 5

correspond to the B-tubulin gene by Sanger sequencing. For gel source data, see Supplementary Figure 3B. (**C**) Dot plot alignment between the Sanger sequenced B-tubulin gene and the B-tubulin transcript (obtained from the RNA-seq *de novo* assembly). The dot plot was performed using the online *blastn suite*. The different lengths between the lanes 4 and 5 and the lane 6 is explained by the presence of two intronic sequences in this gene. B-tubulin forward primer sequence: GCAGATGCTTAACGTCCAGAGC. B-tubulin Reverse primer sequence: GATGCCTCCTGGTACTGCTGG.

DNA was extracted from a pooling of multiple cultures to achieve the required amounts for DNA library preparations. Extractions were done with PureLink® Genomic DNA Mini Kit following the standard protocol. From the extracted DNA, two libraries were prepared for paired-end and mate-pair sequencing (PE and MP, respectively). Each library was sequenced in a 20% Illumina HiSeq 2500 lane using the sequencing kit HiSeq v4 chemistry. The insert sizes for PE and MP were 560 bp and 3000 bp, respectively, and 125 bp of read length. Library preparation and DNA sequencing of *M. vibrans* and other species were done at the CRG Genomics unit (Barcelona).

1.2) Read pre-processing

We followed different pre-processing strategies for PE and MP reads. PE reads were preprocessed with *trimmomatic*[4] *v0.36* using the following parameters: *SLIDINGWINDOW:12:30 LEADING:30 ILLUMINACLIP:2:30:10 MINLEN:80*. TruSeq-PE related adapter sequences from *trimmomatic* were used as contaminant database for *ILLUMINACLIP*. To validate that the *ILLUMINACLIP* parameter did not substantially trim false positive adapter reads, we preprocessed a set of simulated PE reads from *Capsaspora owczarzaki* genome, the closest relative to *M. vibrans*. *C. owczarzaki* genome was not sequenced using Illumina chemistry, and hence non TruSeq-PE related adapters are expected among the simulated reads. Reads were simulated using *DWGSIM v0.1.11* [-e 0 -E 0 -C 40 -1 125 -2 125] (https://github.com/nh13/DWGSIM). Only 21 of the 9420724 of the simulated reads were trimmed [*ILLUMINACLIP:2:30:10 MINLEN:80*]. Hence, we expect a negligible false discovery rate from this adapter trimming strategy. We used *FastQC v0.11.5* during all pre-processing steps for read quality assessment (www.bioinformatics.babraham.ac.uk/projects/fastqc). Before *trimmomatic* [*SLIDINGWINDOW:20:30 LEADING:30 ILLUMINACLIP:2:30:10 MINLEN:50*], MP reads were preprocessed using *nxtrim*[5] *v0.4.1* [*--separate --justmp*] in order to keep only reads in mate-pair orientation as well as to remove Nextera Transposase sequences. For MP, the contaminant database also included Mate Pair Adapter Sequence Elements (see Table 1 in: https://www.illumina.com/documents/products/technotes/technote_nextera_matepair_data_processing.pdf).

1.3) First round of read decontamination

Because we expected a fraction of the sequenced reads to correspond to bacterial contamination (at least from *E. aerogenes*), prior to a definitive genome assembly, we decided to do a draft assembly in order to identify the contaminant contigs and remove the corresponding reads.

For the genome assemblies, we decided to use *SPAdes*[6] *v3.10.1* software because (1) it produced the best assemblies in previous studies of our laboratory that included genome data from unicellular relatives to *M. vibrans*[7], (2) it allows to combine assemblies with different k-mers, (3) it allows to perform both read error and contig miss-match corrections, and (4) because the --meta parameter (i.e., *metaSPAdes*) supports metagenomic data with uneven coverage. For the preliminary assembly (first assembly), we ran *SPAdes* using only the preprocessed paired PE reads and considering the input data as a metagenome [--meta, only non-default software parameters will be specified].

1.3.1) Evaluation of contaminant sources

We first inspected the assembled contigs in order to detect and remove potential vector and/or adapter sequences not trimmed during the pre-processing of the reads. To do so, we used *BLASTn*[8] with the parameters recommended by *VecScreen* documentation (www.ncbi.nlm.nih.gov/tools/vecscreen) [-task blastn -reward 1 -penalty -5 -gapopen 3 -gapextend 3 -dust yes -soft_masking true -evalue 700 -searchsp 1750000000000], using UniVec database as reference. We removed from the assembly two contigs that aligned with high identity and query coverage with UniVec sequences.

We then evaluated the contigs for potential sources of eukaryotic and prokaryotic contamination. For eukaryotic contamination, we searched for 18S ribosomal and mitochondrial sequences. In 18S searches, we aligned an in-house curated database of 18S sequences from a variety of eukaryotic groups with the assembled contigs using *BLASTn* [-evalue 1e-20]. In mitochondrial searches, we aligned with *tBLASTn* [-evalue 1e-20] the cytochrome c oxidase subunits I and III (COX-I, COX-III) and the cytochrome b (Cyt-b) protein sequences from *Andalucia godoyi*[9], as these three proteins are found in most of mitochondrial genomes. To check for prokaryotic contamination, we aligned contigs with a local 16S ribosomal database downloaded from NCBI. All contigs found to be potential 18S, mitochondrial or 16S sequences were aligned with the NCBI nt online database and alignment results were manually inspected. We only found 18S and mitochondrial contigs corresponding to *M. vibrans*, suggesting the absence of eukaryotic contamination. However, we found 16S sequences corresponding to *E. aerogenes* but also to *Stenotrophomonas maltophilia*, which suggested an unexpected potential contamination also from this bacterial species.

1.3.2) Taxonomic classification

We used indirect and direct sequence-similarity based approaches to classify contigs into potential contaminant or potential *M. vibrans*. The indirect strategy consisted of classifying contigs according to the average taxonomic signal shown by their preliminary predicted genes. For that, the genome was preliminary annotated with *BRAKER1*[10], a RNA-seq-based annotation pipeline that combines *GeneMark-ES/ET*[11] *v4.33* and *AUGUSTUS*[12] *v3.1.0*. *M. vibrans* RNA-seq reads were downloaded from NCBI (SRX096925 and SRX096927), corrected with *SEECER*[13] *v.0.1.3* and aligned with contigs using *TopHat*[14] *v2.1.1*. The *accepted_hits.bam* file was used as input for *braker.pl* v1.9. Predicted proteins were then aligned [*BLASTP*: -task blastp-fast, -evalue 1e-3] with an in-house database including all the prokaryotic Uniprot reference proteomes and 25 eukaryotic proteomes [euk_prok_db], mostly from Opisthokonta but also at least one proteome from all the major eukaryotic groups[15]. We then classified each gene into Eukaryote (E), Bacteria (B), Putatively Eukaryote (PE), Putatively Bacteria (PB) or Unknown (?) according to the following criteria: (a) If the best hit of a protein was an eukaryotic sequence and the second best hit as well, the corresponding gene was categorized as 'E'. However, if the second best hit was a prokaryotic sequence, and the division between the exponents of the E-values from the second and the first best hits was less than 0.75, the gene was categorized as 'PE'. (b) If the best hit of a protein was a prokaryotic sequence and there were no hits with an eukaryotic sequence, the corresponding gene was categorized as 'B'. In the opposite case, if the division between the exponents of the E-values from the best hit with an eukaryotic sequence and the best hit was higher than 0.75, the corresponding gene was categorized as 'PB'. Genes that did not align were categorized as '?'. Finally, contigs were classified as potentially *M. vibrans* ('Euk profile') or as potentially contaminant ('Bact profile') when the E+PE/B+PB ratio was >1 or <1, respectively. We excluded from this classification contigs with less than 2500 bp.

For the direct approach, we followed two strategies. The first one (automated strategy), consisted in aligning all the assembled contigs with the NCBI nt database [*BLASTn*: -task megablast -evalue 1e-5], and classify those contigs whose best hit was a prokaryotic sequence as contaminants ('Automated Bact +'). For the second strategy (curated strategy), we separately aligned contigs with the NCBI nt database [*BLASTn*: -evalue 1e-5]. By using *nucl_gb.accession2taxid*, *nodes.dmp* and *names.dmp* files, we ranked the organisms represented in the database according to the number of best hits received by their sequences. Organisms with more than 5 hits were considered as potential contaminants, and their genomes were included into a contaminants database. In particular, the contaminant database included the genomes of *E. aerogenes* and *Stenotrophomonas maltophilia*, but also of other bacterial and eukaryotic species that received few hits. We then aligned contigs with this contaminant database [*BLASTN*: -evalue 1e-30], and classified as bacterial contaminants those that aligned with its best scoring database sequence with > 90% of total query coverage and average identity. The total query coverage is the percentage of contig positions that aligned in any hit with the best scoring database sequence.

To compute the average identities, we first assigned to each aligned position the highest identity value among the hits in which that position aligned, and then the average was computed for every contig.

Contigs that aligned with the contaminant database but that did not satisfy the above-mentioned thresholds were aligned with the NCBI nt online database and alignment results were manually inspected (no evidence of contamination was found on these). All contigs classified as contaminants using this approach (‘Curated Bact +’) corresponded to *E. aerogenes* and *S. maltophilia*, suggesting there are no further contaminant sources. These two contaminant sources were already detected in the fast round of contamination assessment, confirming that the screening of the 18S, 16S and mitochondrial contigs was a valid approach for the identification of contaminant sources in this assembly.

From the results of the taxonomic classification approaches, all contigs in the assembly can belong to one or more of the following categories: ‘Curated Bact +’ for the curated strategy, ‘Automated Bact +’ for the automated strategy, and ‘Euk profile’ or ‘Bact profile’ for the indirect strategy (the last two are mutually exclusive). We also created a ‘No data’ category for contigs not classified in any of the four previous categories. The 57.05% of the assembly (in terms of length) was classified as ‘Euk profile’ (Supplementary Information 1-Fig. 4), suggesting that most of the data is likely to correspond to the *M. vibrans* genome (already expected from the 18S/16S heteroduplex PCR results, see Supplementary Information 1-Fig. 3). A 14.02% of the assembly was in ‘No data’ category. This includes the *M. vibrans* mitochondrial genome, from which no genes were predicted by *BRAKER1* (probably because of differences in the genetic code). Very few contaminant contigs were expected in ‘No data’, since the sum of the lengths of those contigs identified as *E. aerogenes* and *S. maltophilia* by the curated strategy is similar to the expected length of both genomes. Thus, most ‘No data’ contigs likely represent artefactual contigs or non-coding regions of *M. vibrans* genome. The contigs that are neither in ‘Euk profile’ nor in ‘No data’, which correspond to the 28.90% of the data, are potential bacterial contaminants because of being in at least one of these categories: ‘Curated Bact +’, ‘Automated Bact +’ or ‘Bact profile’. The vast majority of them were contaminants according to the three categories (25.08% of the data). All contigs classified as ‘Curated Bact +’ were also classified as ‘Bact profile’ and/or ‘Automated Bact +’. However, these two strategies also identified as contaminants contigs that were not detected by the curated strategy (3.20% of the data).

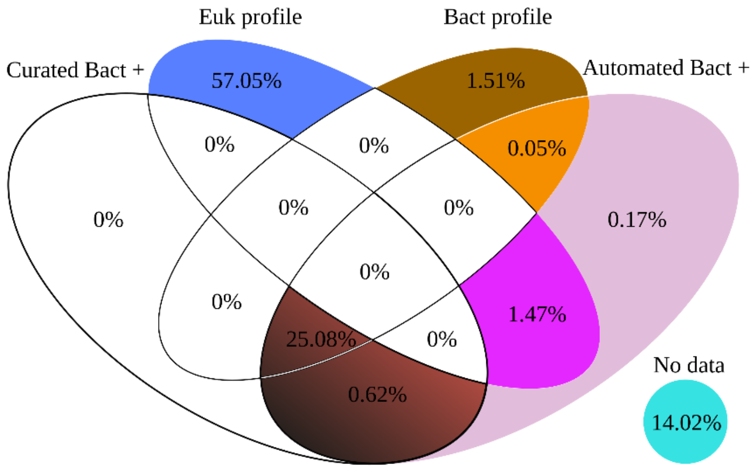

Supplementary Information 1-Fig. 4. Venn diagram showing the distribution in percentage of total length of the *M. vibrans* contigs from the first assembly for each possible category according to the taxonomic classification.

1.3.3) Tetranucleotide-distance clustering using ESOM tools

We also classified contigs using Databionics ESOM Tools[16], an unsupervised compositional approach that allows the clustering of contigs according to similarities in their tetranucleotide frequencies. This methodology has been proved to be a successful contig binning tool for metagenomic datasets even at the taxonomic level of genus[16]. First, we used *esomWrapper.pl* (available in https://github.com/tetramerFreqs/Binning) to calculate the tetranucleotide frequencies. Briefly, a 1-bp sliding window was used to count for the tetranucleotides of both strands of the contigs. Contigs <2.5 kb were previously discarded and those > 5 kb were split into contig windows of 5 kb length. Data was normalized by fragment length and also with the Robust ZT option. We set the training parameters as specified by [16], changing only the row and column numbers by the values indicated in *esom.log* file. Once the training step was completed, we visualized the clustering results in a map with the UMatrix background and the bwparatoumx gradient (Supplementary Information 1-Fig. 5). Each contig is represented by a dot and colored according to the corresponding taxonomic classification (Supplementary Information 1-Fig. 4). The position of each contig in the map depends only on its tetranucleotide frequency and is independent of the taxonomic classification. Due to differences in the genome-wide compositional features between taxa, contigs from the same genome are expected to cluster together[16]. Tetranucleotide frequency distances between neighbour contigs are represented with a white/black background gradient for smaller and larger distances, respectively.

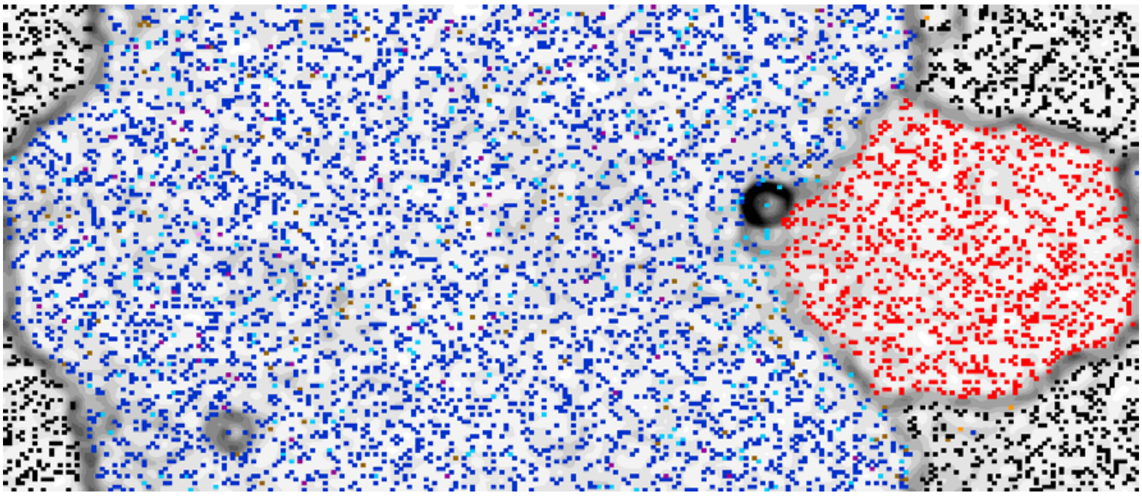

Supplementary Information 1-Fig. 5. ESOM map of the *M. vibrans* contigs from the first assembly. Each dot in the map correspond to one contig/contig window, which are colored according to the area where the corresponding contig was included in the Venn diagram shown in Supplementary Information 1-Fig. 4. Tetranucleotide frequency distances between neighbour contig/contig windows are represented with a white/black background gradient for smaller and larger distances, respectively.

The topology of the map shows two big clusters that include all contigs identified as *E. aerogenes* and *S. maltophilia* by the curated strategy (see red and black dot regions, respectively; note that the map is continuous from top to bottom and side to side). The black dot region also includes five dots of distinct color (i.e., fragments that were not detected as *S. maltophilia* but that apparently share compositional similarity to *S. maltophilia* detected contigs). The vast majority of contigs classified as 'Euk profile' and 'No data' appear out of the two bacterial regions of the map, suggesting that the large region in the middle corresponds to *M. vibrans* genome, with the only exception of one dark blue contig included in the *S. maltophilia* cluster. This suggest a good precision for the indirect approach in identifying the contigs corresponding to the *M. vibrans* genome. All 'Euk profile' contigs classified as bacterial by the automated strategy ('Automated Bact +') are found in the *M. vibrans* region (see dark purple dots). Moreover, the vast majority of contigs classified as contaminants by the indirect and automated strategies but not by the curated strategy are also found in *M. vibrans* region (colored in orange, brown and pink). We thus expect most of these contigs to have been misclassified as contaminants because of false positive alignments with bacterial sequences (we used relaxed E-value thresholds in the *BLAST* searches). Also within the *M. vibrans* region, two smaller clusters are observed: a first one in the left bottom includes two 'Euk profile' contigs; a second one located near the red bacterial region includes one 'No data' contig (light blue color). Below to the second cluster, there are also some contigs which we consider in-between the *M. vibrans* and the *E. aerogenes* regions. All these uncertain contigs (see yellow dots in Supplementary Information 1-Fig. 6) were aligned with the NCBI nt online database and results were manually inspected. We finally decided to include three

of these suspicious contigs, which were classified as contaminants according to the automated or indirect strategies, into the 'Contaminant set', which also included all 'Curated Bact +' contigs. Other contigs evaluated in ESOM were included into the 'Non-contaminant' set.

Overall, while the results shown by the sequence-similarity and the tetranucleotide distance approaches are highly consistent between them, the combination of both methodologies was necessary to detect the few contigs that were misclassified by either the taxonomic or the ESOM approaches. Among the three strategies used for taxonomic classification, the results from ESOM proved that the curated strategy was the most accurate. However, this strategy was only applicable to *M. vibrans* data because the genomes of the two contaminants were available. The combination of the automated and the indirect strategies also allowed to detect all contaminant contigs, at the expense of some false positives. However, these false positive cases were later corrected with ESOM, this pointing that the combination of the automated and indirect strategies with a tetranucleotide distance analyses are good alternatives for complex metagenomic data. On the one hand, the automated strategy should work for data with undetermined contaminant bacteria because it uses the NCBI nt as database. On the other hand, given that amino acid sequences allow to detect homology at larger evolutionary distances than nucleotides, the indirect strategy should work for cases in which the contaminant genome is not available.

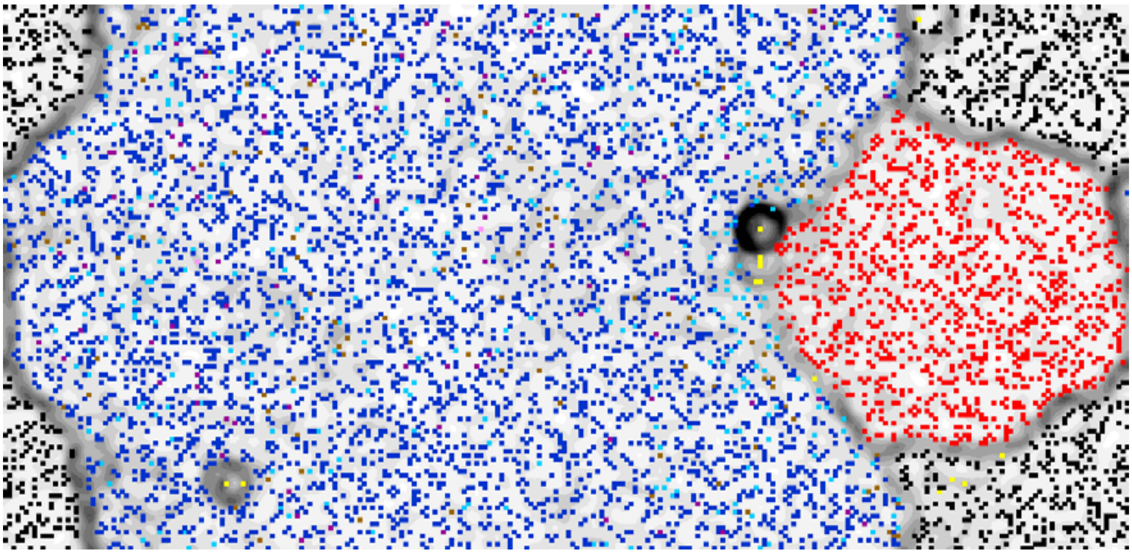

Supplementary Information 1-Fig. 6. ESOM map of the *M. vibrans* contigs from the first assembly, as in Supplementary Information 1-Fig. 5, but colored in yellow those contig/contig windows that were further inspected in alignments against NCBI databases.

1.4) <u>Second round of read decontamination</u>

We expected that a second assembly, after having removed at least a major fraction of the contaminant reads, will greatly reduce potential uneven coverage problems, which are typical in metagenomic data and may had limited the quality of the first assembly. The absence of this

constraint enables the usage of higher k-mers values and the mismatch correction mode during the assembly (incompatible with the metagenome mode in *SPAdes*).

We first removed from the PE and MP libraries the reads that aligned with the ‘Contaminant’ but not with the ‘Non-contaminant’ post-ESOM sets. For PE reads, we only removed them if both paired reads satisfied this criterion. We used *bowtie2*[17] *v2.2.9* for read alignments. Then, the surviving reads were assembled with *SPAdes* [-k 53,75,91,109 --careful --hqmp1-fr --cov-cutoff auto]. The average coverage of the 109-mer assembly was 46.65.

We next compared the completeness and the contiguity of this second assembly with the first assembly. Completeness and contiguity were estimated by aligning a set of *de novo* assembled *bona fide* and non-redundant *M. vibrans* transcripts with both assemblies [*BLASTn*: -evalue 1e-3]. In particular, we estimated completeness by counting how many transcripts aligned with the genome with an average identity of >95% and with a total query coverage of >95%. Contiguity was estimated as completeness, but only hits with the best scoring target scaffold were considered (i.e., a transcript which sequence is complete but fragmented into distinct scaffolds will sum for completeness but not for contiguity). The set of *bona fide* and non-redundant *M. vibrans* transcripts was chosen first by reducing redundancy with *CD-HIT*[18] *v4.6* [-c 0.70], and second by keeping only those transcripts without prokaryotes among whose three *BLASTx* best targeting species (a total of 10056 transcript sequences) [-evalue 1e-3, -db euk_prok_db]. Completeness and contiguity measures were found to be better for the second assembly (9577 and 8889, respectively) than for the first assembly (9520 and 8348, respectively). This supports the strategy of re-assembling the non-contaminant reads identified during the decontamination of the first assembly, and also agrees with our decontamination approach performing well in terms of specificity (i.e., very few *M. vibrans* genomic data was misidentified as bacterial contaminant). Indeed, we only found 13 transcripts present in the first assembly that were not recovered in the second assembly. We hence added to the second assembly the 8 contig fragments (3441 bp) of the first assembly to which those 8 transcripts aligned (their sequence names include the suffix '_fromdraftassembly').

Scaffolds were next submitted to a second round of decontamination. We first searched for potential remaining vector/adapter sequences using *BLASTn* with UniVec database (explained in 1.3.1). We removed one short scaffold (360 bp) and we also detected two scaffolds likely containing contaminant sequences related to Illumina technology (we chose a score cutoff value of >36.5 to distinguish between true and false positive contaminants, since it was the highest value with which a scaffold from *C. owczarzaki*, the closest relative to *M. vibrans* and whose genome was not sequenced with Illumina technology, aligned to a UniVec target related to Illumina technology). We removed the aligned regions of the other two scaffolds and hence each one was split into two sub-scaffolds. We did that just to ensure that our assembly did not include

fragments that were misassembled because reads with contaminant sequences connected during the de Bruijn graph step[6].

Before ESOM analyses, scaffolds were taxonomically-classified into eukaryotic/bacterial using the indirect and the automated approaches (both explained in 1.3.2). Scaffolds were split in different categories, according to the results from the taxonomic classification: ‘Bact profile’, ‘Euk profile’, ‘Automated Bact +’, ‘No data’ (see 1.3.2); and we also considered an extra category to include contigs classified as ‘Euk profile’ and ‘Automated Bact+’ (‘Euk profile, Automated Bact+’). Because in ESOM each scaffold can only be present within one category, those detected as bacterial by the two approaches were included in ‘Bact profile’ but not in ‘Automated Bact+’. Finally, we also incorporated into ESOM analyses all contaminant contigs from the first assembly.

We did not find scaffolds with windows present inside and outside the region of the map with the contaminant contigs from the first assembly (colored in black in Supplementary Information 1-Fig. 7). This suggests the absence of *M. vibrans/Bacteria* chimeric scaffolds. ‘Euk profile’ and ‘Euk profile, Automated Bact+’ scaffolds that localized within the bacterial region were further inspected by means of online *BLASTn/BLASTx* searches (colored in yellow in Supplementary Information 1-Fig. 8). Scaffolds surrounding grey areas outside the bacterial region (i.e., the region including most of ‘Euk profile’ scaffolds, filled mostly with dark blue dots in Supplementary Information 1-Fig. 7) were also inspected (colored in pink in Supplementary Information 1-Fig. 8). While the white/black gradient suggests a distinct compositional pattern in these scaffold windows respect to the average genome, we did not remove them because alignment results did not suggest contamination either from *E. aerogenes/S. Maltophilia* scaffolds or other genomes. Moreover, most of them have eukaryotic genes annotated, and we already rejected the possibility of eukaryotic contamination during the decontamination of the first assembly. Still, four of these scaffolds, with a length between and 2093 and 6202 bp, have bacterial but not eukaryotic genes annotated. Because of this, we added the tag "_potentialcontaminant" as a suffix in their scaffold names in the FASTA file.

All scaffolds found outside the bacterial region as well as those labeled as "_potentialcontaminant" were included in the *Mvib.gDNA.clean.v1.fasta* file (all FASTA files produced will be available online as soon as we publish the corresponding manuscript). Because scaffolds with <2000 bp were not considered during ESOM analyses, by default we did not include them in this set, with two exceptions. First, those that were aligned by the set of reliable transcripts. Second, those with eukaryotic genes annotated. In both cases, those scaffolds that also had one bacterial or potential bacterial gene annotated were also labeled with the suffix "_potentialcontaminant". As a final step to ensure that we kept only *bona fide M. vibrans* scaffolds, we aligned them [-evalue 1e-5] with the contaminant database created during the curated strategy (see 1.3.2). Alignment results only suggested one scaffold (141 bp) as potential contaminant, and hence was also labeled as

"_potentialcontaminant". In total, 27 of 2860 scaffolds (20516 bp of 29797085) were labeled as "_potentialcontaminant". We also labeled the names of the scaffolds containing the mitochondrial genome (assembled in a single scaffold; 55949 bp) and the 18S genes as "_mitochondrial" and "_ribosomal", respectively.

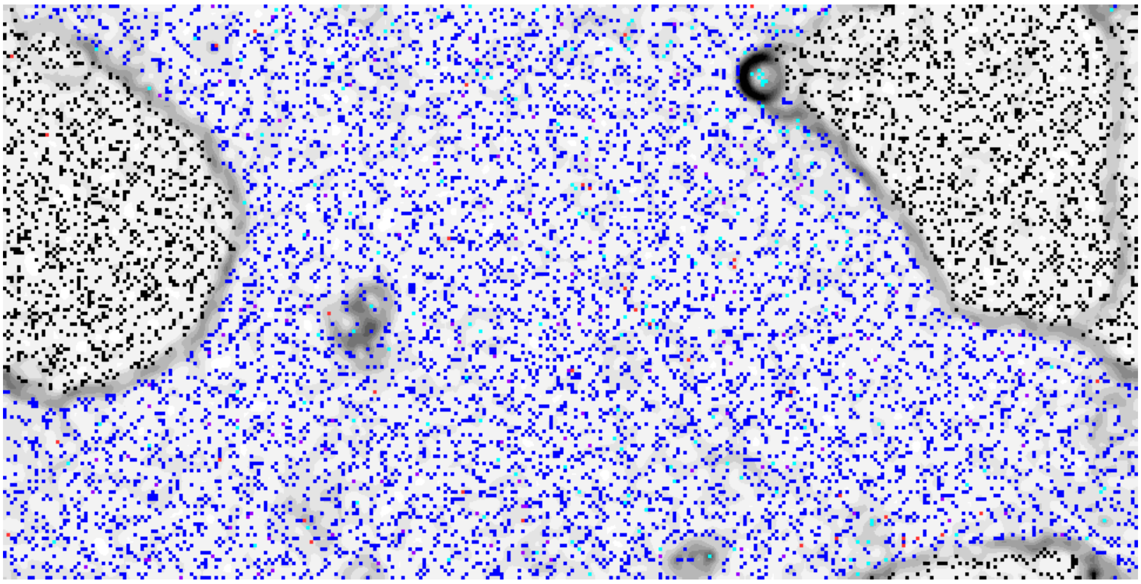

Supplementary Information 1-Fig. 7. ESOM map of the *M. vibrans* scaffolds from the second assembly. Each dot in the map correspond to one scaffold/scaffold window. Tetranucleotide frequency distances between neighbour scaffold/scaffold windows are represented with a white/black background gradient for smaller and larger distances, respectively. Color code: red for 'Bact profile', dark blue for 'Euk profile', green for 'Automated Bact +', light blue 'No data' (see previous sections) purple for 'Euk profile, Automated Bact+', and black for contaminant contigs from the first assembly.

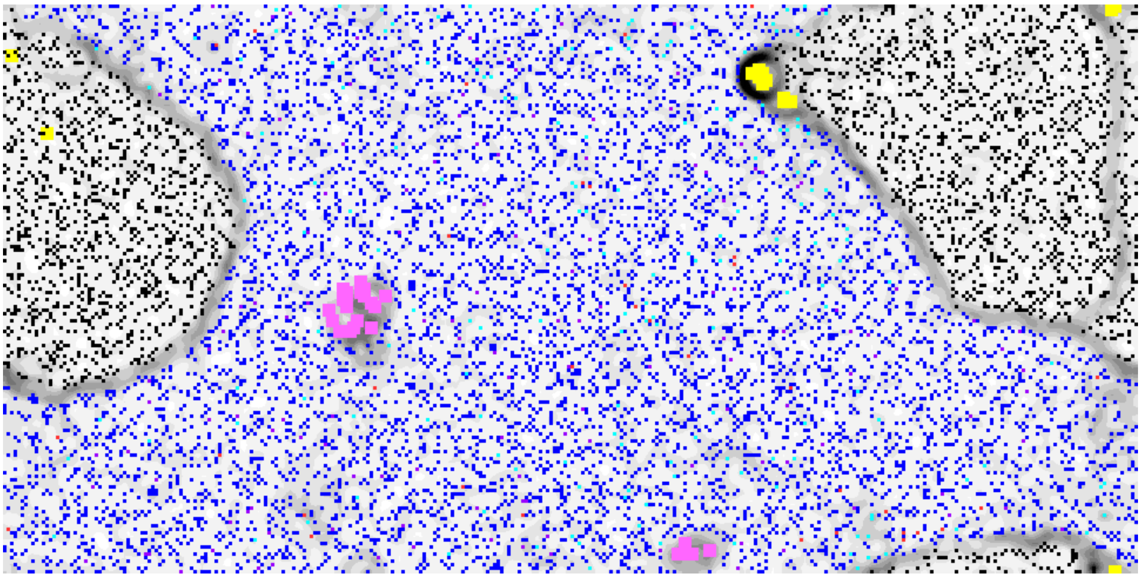

Supplementary Information 1-Fig. 8. ESOM map of the *M. vibrans* scaffolds from the second assembly, as in Supplementary Information 1-Fig. 7, but colored in yellow and pink those scaffolds/scaffold windows that were further inspected in alignments against NCBI databases.

1.5) <u>Benchmarking our supervised binning approach by comparing it to *CONCOCT* (unsupervised approach)</u>

As shown in previous sections, we used a supervised approach to classify (or bin) genomic fragments from the *M. vibrans* metagenome, based on the taxonomic annotation and the tetranucleotide distance-based clustering (i.e., ESOM). At the time of this analysis, unsupervised binning tools such as *CONCOCT*[19] were already proved to be successful with complex metagenomic data (e.g., [20]). We compared the performance of our approach with the results provided by *CONCOCT v0.4.1*.

CONCOCT grouped 4525 of the 20548 contigs from the first assembly into 61 bins, this corresponding to 94.95% of the assembly length (excluding vectors). Regarding to the detection of contaminant contigs, in our binning approach of the first assembly, 1008 of the contigs (10331925 bp) were classified as bacterial contaminants after ESOM analyses. The *CONCOCT* bins 10 and 60 included 83 of these 1008 contigs (10091058 bp). However, these two bins also included one and two contigs, respectively, that were not classified as bacterial by our approach. Based on the analyses of the *BLAST* results with NCBI nt, two of these three contigs would be *bona fide* bacterial, and hence were misclassified by our approach as non-bacterial. The other contig could have been misclassified as bacterial by *CONCOCT*. Apart of the bins 10 and 60, the bins 44, 56 and 58 also included in total 7 additional contigs classified as bacterial by our approach (3, 3 and 1 contigs, respectively; 12121 bp). Despite a manual inspection of *BLAST* results suggest that these 7 contigs would be *bona fide* bacterial, the bins 56 and 58 also included 4 and 52 contigs that were not classified as bacterial by our approach. The 4 contigs within the bin 56 would be *bona fide* non-bacterial (indeed, the *M. vibrans* 18S ribosomal gene is one of these contigs). In the case of bin 58, only 1 of the 52 contigs would be *bona fide* bacterial.

Regarding the detection of *M. vibrans* contigs, 4432 of the 19537 contigs that we classified as *M. vibrans* were included in *CONCOCT* bins. These corresponds to the 93.97% of length spanned by the 19537 contigs (29783450 bp, excluding the 3 contigs that our approach misclassified as non-bacterial in the binning of the first assembly); indicating that ~6% of the data would have been directly lost because of not having been included in any bin. 4376 contigs that are non-bacterial according to our approach are in those *CONCOCT* bins that did not include *bona fide* bacterial contigs (93.43% of 29783450 bp). Among them, the bin 40 includes 20884537 bp (2082 contigs) and none of the *bona fide* bacterial contigs, suggesting that it includes a substantial fraction of the *M. vibrans* genome. The remaining non-bacterial contigs according to our binning approach (7104273 bp) are distributed in 59 other *CONCOCT* bins. We consider that this extra number of

bins does not reflect the presence of further contaminants sources, but instead of different compositional features or uneven coverage between distinct regions of the *M. vibrans* genome. First, because the comprehensive sequence-similarity and ESOM analyses done did not indicate the presence of further contaminants apart of the two bacterial species. Second, because 98.04% of the data that we considered as *M. vibrans* genome were in *CONOCT* bins in which at least one contig was aligned by a *bona fide M. vibrans* transcript (RNA-seq *de novo* assembled transcripts whose best hit with euk_prok_db was a *C. owczarzaki* protein [the phylogenetically closest genome to *M. vibrans* available], and whose three best target species are eukaryotes). Thus, *CONCOCT* most likely put a substantial fraction of *M. vibrans* genome (7104273 bp) in separate bins, some of which include bacterial contigs. Indeed, the bin 56 is a good example of this, as it includes the *M. vibrans* 18S ribosomal gene together with three *bona fide* bacterial contigs and three *bona fide* non-bacterial contigs, this showing the limitations of unsupervised approaches when dealing with complex regions of eukaryotic genomes (e.g., unusual coverage or compositional features).

Overall, these results suggest that our approach would have been more accurate than *CONCOCT* with the *M. vibrans* data. On the one hand, although *CONCOCT* binned most of the *bona fide* bacterial data into two bins (as expected), 7 *bona fide* bacterial contigs were misclassified in bins mostly composed by *bona fide M. vibrans* contigs. Moreover, one *bona fide* non-bacterial contig was included in one of the two bins that include most of the bacterial contigs. Despite our binning approach in the first assembly misclassified three contigs as non-bacterial, they were probably eliminated during the decontamination of the second assembly, since we did not found them in *Mvib.gDNA.clean.v1.fasta*. On the other hand, *CONCOCT* split the *bona fide M. vibrans* contigs in 59 bins, whereas we grouped them as a single bin (i.e., *Mvib.gDNA.clean.v1.fasta*). Another relevant feature is that 5.05% of the data was not included in any *CONCOCT* bin. We thus concluded that our approach was more accurate than the unsupervised approach, and hence we also used it to decontaminate the genomes of *P. atlantis* and the two *Pigoraptor* species.

2) *Parvularia atlantis* (Nucleariids, Opisthokonta)

2.1) Cultures, Cell cytometry and DNA sequencing

We started from cultures of *P. atlantis*[21] (formerly Nuclearia sp. ATCC 50694) growing in ATCC Medium 802 and maintained at 23 ºC. As the initial *M. vibrans* culture, *P. atlantis* grow in non-axenic conditions with an undetermined diversity of contaminant bacteria. Because we could not isolate and grow *P. atlantis* in better culture conditions, we directly extracted DNA from a pooling of cells sorted by flow cytometry analyses (see *M. vibrans* section for FACS and DNA extraction protocols). The cell sorting strategy allowed to enrich the *P. atlantis*/Bacteria ratio but not to get rid of bacterial contamination, as shown by the presence of amplified 16S ribosomal PCR-product in the extracted DNA (Supplementary Information 1-Fig. 3B). A total of 264 ng were obtained from

the extraction. This yield of DNA was enough to construct a PE but not a MP library (we only did MP library for *M. vibrans*). The PE library was prepared and sequenced in a 50% Illumina HiSeq 2500 lane using the sequencing kit HiSeq v4 chemistry (read insert size: 520 bp; read length: 125 bp).

PE reads were preprocessed with *trimmomatic*, using the following parameters: *LEADING:30 TRAILING:20 SLIDINGWINDOW:2:20 ILLUMINACLIP:2:30:10 MINLEN:80*. As with *M. vibrans*, TruSeq-PE related adapter sequences from *trimmomatic* were used as contaminant database for *ILLUMINACLIP*. The single and paired preprocessed reads were submitted to a read correction step using *SPAdes* [--only-error-correction].

2.2) <u>Benchmarking of metagenomic assemblers</u>

The genomic data of *M. vibrans* was a simple metagenome case, where most of the reads belonged to the eukaryotic species, with also reads belonging to two well defined contaminant bacteria. In the case of *P. atlantis*, despite we enriched the proportion of eukaryotic cells by cell cytometry sorting, we also expected a substantial fraction of reads from an uncertain diversity of contaminant bacterial species (Supplementary Information 1-Fig. 3B). Hence, we decided to benchmark *metaSPAdes* (used for *M. vibrans*) with two other popular metagenome assemblers available at that time: *IDBA-UD*[22] and *Ray*[23] *Meta v.2.3.1*. In particular, we evaluated standard contiguity metrics (e.g., N50, L75) and also the tendency to assemble potential chimeric contigs.

For that, the three metagenome assemblies were ran using the preprocessed and corrected paired and unpaired reads, with default assembly parameters. Because we were interested in the fraction of the assembly corresponding to *P. atlantis*, we ran *BUSCO*[24] *v1.22*, using the *all Eukaryota* dataset, in order to obtain the *BUSCO orthologs* from our metagenomes. We then aligned these *BUSCO orthologs* with euk_prok_db, and we kept those whose best scoring hit was a eukaryotic protein. We expected at least most of the contigs encoding these *bona fide* eukaryotic *BUSCO orthologs* (*BUSCO contigs*) to correspond to *P. atlantis* genome. *BUSCO contigs* were annotated using the *BRAKER1* pipeline, and every predicted gene was taxonomically classified into eukaryotic or bacterial following the indirect strategy (see 1.3.2). RNA-seq reads, required by the *BRAKER1* pipeline, were downloaded from NCBI (SRR1617645), preprocessed using *trimmomatic* [ILLUMINACLIP:2:30:10 SLIDINGWINDOW:4:5 LEADING:5 TRAILING:5 MINLEN:25], and corrected using *SEECER*.

Completeness and contiguity of *BUSCO contigs* were estimated with *QUAST*[25] *v4.2* and *BUSCO*. In agreement with the bibliography[26], *metaSPAdes* outperformed *IDBA-UD* and *Ray Meta* in most of the metrics (see Supplementary Information 1-Table 1 below). *metaSPAdes* was also the assembler with the highest number of both eukaryotic and bacterial genes in *BUSCO contigs*, and showed the lowest ratio of *BUSCO contigs* without bacterial genes. While these results could

be interpreted as *metaSPAdes* being the assembler with more chimeric contigs, they could also be explained because *BUSCO contigs* were more and longer than in the other two assemblers. Moreover, because not all genes annotated as bacterial are necessarily *bona fide* contaminants (e.g., horizontal gene transfer, low scoring *BLAST* hits with bacterial sequences -which are more represented than eukaryotes in our database-, etc.); not every contig with genes annotated as bacterial is necessarily a chimera.

Because each species had its own abundance in the sequenced sample, differences in coverage between those genomic fragments corresponding to *P. atlantis* and those corresponding to contaminant bacteria can be expected. If so, and if there are chimeric contigs in the assemblies, which is uncertain, we would expect them to probably correspond to those contigs showing larger differences between the coverages of the regions corresponding to eukaryotic genes and those corresponding to bacterial genes. We hence considered the number of *BUSCO contigs* showing elevated internal coverage differences as a proxy to estimate the tendency of every assembler to construct potential chimeric contigs. *BUSCO contigs* with internal coverage differences were determined in the following manner: for each *BUSCO contig* that contained eukaryotic and bacterial genes, we computed (i) the coverage of each gene (cov_gene), and (ii) the average coverage of all eukaryotic genes in this specific contig (cov_all_euk_genes). Then, we calculated the absolute distance between the coverage of each gene (eukaryotic or bacterial) and this average (dist_gene = abs(cov_gene - cov_all_euk_genes)). If the highest distance corresponded to a bacterial gene, we considered this contig as a potential chimera. The coverage of each gene was estimated with *samtools*[27] *1.3.1 depth*, previously mapping the clean and preprocessed DNA-seq reads to *BUSCO contigs* using *bowtie2* [--no-mixed --no-discordant --maxins 750].

The number of contigs showing potential chimeric features were the same in *metaSPAdes* and *IDBA-UD*, both lower than in *Ray Meta*. However, *metaSPAdes* showed the proportionally less potential chimeric contigs (i.e., the best), given that the number of *BUSCO contigs* is higher than in *IDBA-UD*. Thus, we concluded that *metaSPAdes* was the best assembler for our data because since it showed better contiguity and completeness measures than the other two assemblers, and this does not seem to come at the expense of increasing the probability of assembling contigs showing chimeric-like features. Our results agree with a published benchmarking of metagenome assemblers, which recommends the use of *metaSPAdes* when the main objective is to retrieve the genome of a species that is well represented in the sample[26].

Supplementary Information 1-Table 1. Benchmarking of three popular metagenomic assemblers for *P. atlantis* data.

# All contigs	**metaSPAdes**	**IDBA-UD**	**Raymeta**
**Complete BUSCOs (total 429)**	275	260	265

**Complete and single-copy BUSCOs**	230	212	218
**Complete and duplicated BUSCOs**	45	48	47
**Fragmented BUSCOs**	72	85	83
**Missing BUSCOs**	82	84	81
**Contigs**	61669	36486	187875
**Contigs > 999 bp**	9647	14036	10078
**N50**	97390	40013	97458
**L75**	1235	2625	1132
**Genome size (Mb)**	158747712	154204313	152524304
**Genome size, only > 999 bp (Mb)**	142696796	143141922	117504526
**Genome size, only > 49999 bp (Mb)**	88898272	69342700	73494015
# Contigs with single copy complete BUSCOs euks (*BUSCO contigs*)			
**Complete BUSCOs (total 429)**	238	211	217
**Complete and single-copy BUSCOs**	237	210	215
**Complete and duplicated BUSCOs**	1	1	2
**Fragmented BUSCOs**	35	24	34
**Missing BUSCOs**	156	194	178
**Contigs**	181	179	179
**Contigs > 24999 bp**	75	30	45
**N50**	35054	20656	26013
**L75**	86	88	85
**Genome size (Mb)**	4600459	2945403	3533535
**Genome size, only > 24999 bp (Mb)**	3190783	1119512	1827248
**Nº euk genes**	1682	1116	1316
**Nº bact genes**	86	56	73
**% euk genes / total genes**	95,14%	95,22%	94,74%
**Contigs with at least 1 bact gene**	118	50	57
**Contigs without bact genes**	63	129	122
**% contigs without bact genes / total contigs**	34,81%	72,07%	68,16%
**Potential chimeric contigs**	16	16	19
**% potential chimeric contigs / total contigs**	8,84%	8,94%	10,61%

2.3) First round of read decontamination

The assembled contigs from *metaSPAdes* were evaluated for potential contamination (see 1.3.1). 19 contigs were removed because they showed >50% of average identity and >95% average coverage with UniVec sequences. We only found one contig showing signatures of a *bona fide* 18S gene, of 10705 bp and high assembly coverage (a metric computed by *SPAdes* for every contig, available in the sequence name). Its best hit with a local 18S database corresponded to the 18S sequence of *P. atlantis*. We also found 5 other contigs aligning to the 18S database, but were all of short length (<302 bp) and had very low assembly coverage values. We also found 71 contigs with potential 16S sequences.

We also searched for potential mitochondrial sequences in contigs (see 1.3.2). 101 contigs were aligned by the *A. godoyi* sequences, most of which were expected to be bacterial contamination. We used the output from *tBLASTn* to extract the putative amino acid sequences from the alignments, and then aligned the extracted sequences with a database including all sequences from prok_db and the NCBI CDS translations of complete mitochondrial genomes [mito_db], downloaded from ftp://ftp.ncbi.nlm.nih.gov/blast/db/ [*BLASTp*: -evalue 1e-3]. We considered as potential mitochondrial sequences 5 contigs whose predicted sequences performed the best hit with a mitochondrial protein. We expect two of these contigs to correspond to *P. atlantis* mitochondria. First, because they have a length of 9773 and 13093 bp and a high assembly coverage. Second, because the three sequences predicted from them (COX-I, COX-II, and Cyt-b) did not align with a perfect identity with NCBI nt. The other three contigs showed residual length and assembly coverage values, and none of them aligned with a perfect identity with NCBI nt, suggesting that, as with the spurious 18S contigs, they likely corresponded to miss-assembled mitochondrial regions of *P. atlantis* rather than to eukaryotic contamination.

Contigs were taxonomically classified using the indirect approach and the automated direct approach (see 1.3.2). All contigs > 2000 bp were submitted to ESOM analyses, splitting contigs larger than 7999 bp into contigs windows of 4000 bp. Before ESOM analyses, contigs were classified into one of the following categories, and colored accordingly in the ESOM map (Supplementary Information 1-Fig. 9): '16S', '18S', 'Bact profile' (excluding contigs in the 16S category), 'BUSCO Automated Bact +' (*BUSCO contigs* classified as bacterial according to the direct approach), 'BUSCO Automated Bact -' (*BUSCO contigs* non-classified as bacterial according to the direct approach), 'Euk profile Automated Bact +' (non-*BUSCO contigs* classified as eukaryotic according to the indirect approach, and as bacterial according to the direct approach), 'Euk profile Automated Bact -' (*non-BUSCO* contigs classified as eukaryotic according to the indirect approach, and non-classified as bacterial according to the direct approach), 'Automated Bact +' (other contigs only classified as bacterial according to the direct approach) and 'No data'.

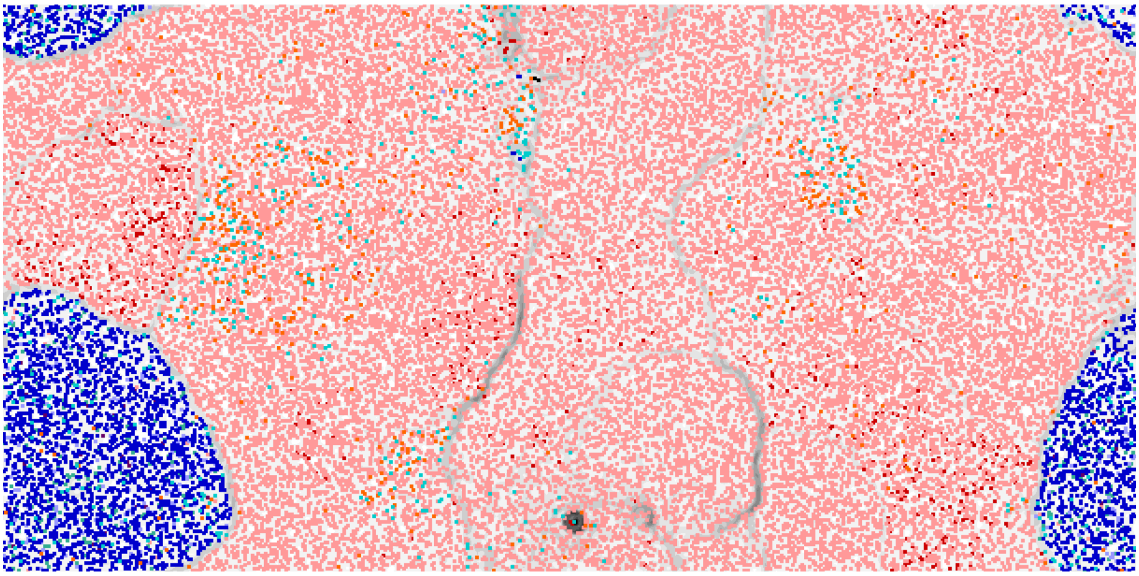

Supplementary Information 1-Fig. 9. ESOM map of the *P. atlantis* contigs from the first assembly. Each dot in the map correspond to one contig/contig window, which are colored according to the category to which was classified. Category color code: red for ‘16S’, black for ‘18S’, pink for ‘Bact profile’, dark purple for ‘BUSCO Automated Bact +’, turquoise for ‘BUSCO Automated Bact -’, light purple for ‘Euk profile Automated Bact +’, dark blue for ‘Euk profile Automated Bact -’, orange for ‘Automated Bact +’, and light blue for ‘No data’. Tetranucleotide frequency distances between neighbour contig/contig windows are represented with a white/black background gradient for smaller and larger distances, respectively.

As expected, *BUSCO contigs* and most of the ‘Euk profile’ contigs clustered together in the map (see dark blue dots regions in Supplementary Information 1-Fig. 9), and we hence considered that region as the *P. atlantis* genome (see dark green dots in Supplementary Information 1-Fig. 10). The ‘Non-contaminant’ set included all contigs in this region, as well as the two contigs previously found to contain mitochondrial sequences. A small fraction of ‘Euk profile’ contigs were found in a separate region in the north of the map, surrounded by contigs classified as ‘Bact profile’, ‘Automated Bact+’ and ‘No data’ (Supplementary Information 1-Fig. 9). We considered these contigs and their neighbours as uncertain (see yellow dots in Supplementary Information 1-Fig. 10). 12 of 53 uncertain contigs were included in the ‘Non-contaminant’ set because they corresponded to the mitochondrial or to the 18S contig or because being ‘Euk profile’. The other 41 uncertain contigs were included in the ‘Contaminant’ set together with the other contigs of the map that were not in the *P. atlantis* dataset.

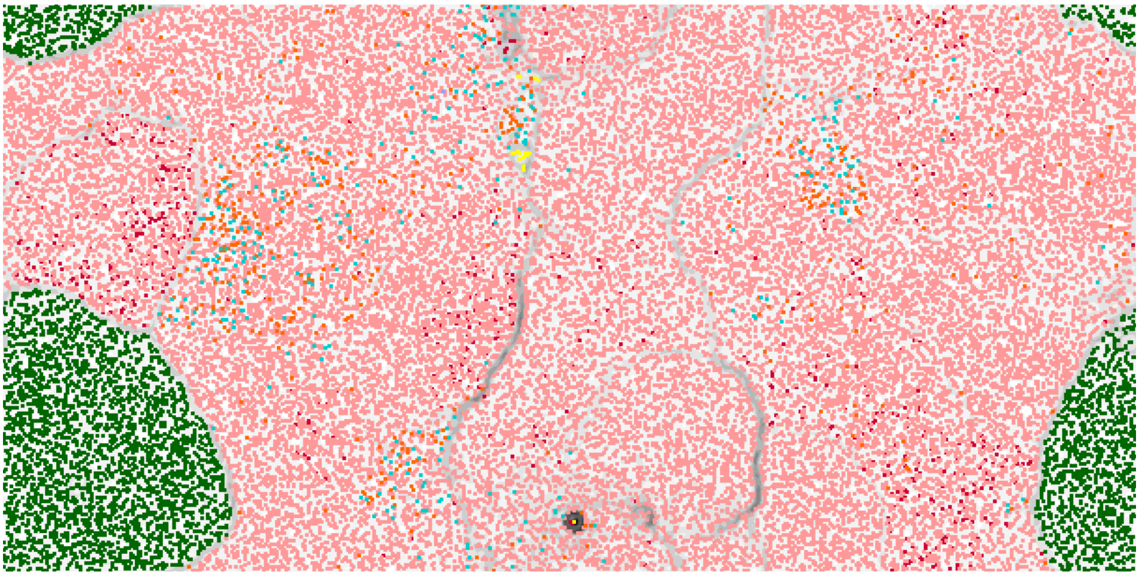

Supplementary Information 1-Fig. 10. ESOM map of the *P. atlantis* contigs from the first assembly, as in Supplementary Information 1-Fig. 9, but colored in green those contig/contig windows included in the *P. atlantis* dataset, and colored in yellow those contig/contig windows that further inspected to determine whether they should be included in the *P. atlantis* dataset, or in the 'Contaminant' set. Other contig/contig windows were included in the 'Contaminant' set.

2.4) Second round of read decontamination

We removed from the PE library the reads that aligned with the 'Contaminant' but not with the 'Non-contaminant' post-ESOM sets (as we did for *M. vibrans*, see 1.4). The surviving reads were assembled using *SPAdes* with the metagenome disabled, and using the following k-mers: 33, 55, 71, 89 [--careful --cov-cutoff auto]. The average coverage of the 89-mer assembly was 36.28. Then, as with the first assembly, we screened the assembled scaffolds for potential contamination of adapter/vector sequences. One scaffold that aligned with Illumina targets with >36.5 *BLASTn* score (see *M. vibrans* sections) was directly removed because of its short length and low coverage. We further removed other 7 scaffolds of short length (3470 bp) because they aligned with vector sequences along most of their sequence. After the adapter/vector decontamination step, scaffolds were taxonomically classified using the indirect approach and the automated direct approach (see 1.3.2).

All scaffolds > 2000 bp were submitted to ESOM analyses, splitting contigs larger than 7999 bp into contig windows of 4000 bp. Before ESOM analyses, contigs were classified into one of the following categories, and colored accordingly in the ESOM map (Supplementary Information 1-Fig. 11): 'Bact profile', 'Euk profile Automated Bact +' (contigs classified as eukaryotic according to the indirect approach, and as bacterial according to the direct approach), 'Euk profile Automated Bact -' (contigs classified as eukaryotic according to the indirect approach, and non-classified as bacterial according to the direct approach), 'Automated Bact +' (contigs only

classified as bacterial according to the direct approach) and ‘No data’. Finally, we also incorporated into ESOM analyses all contaminant contigs from the first assembly.

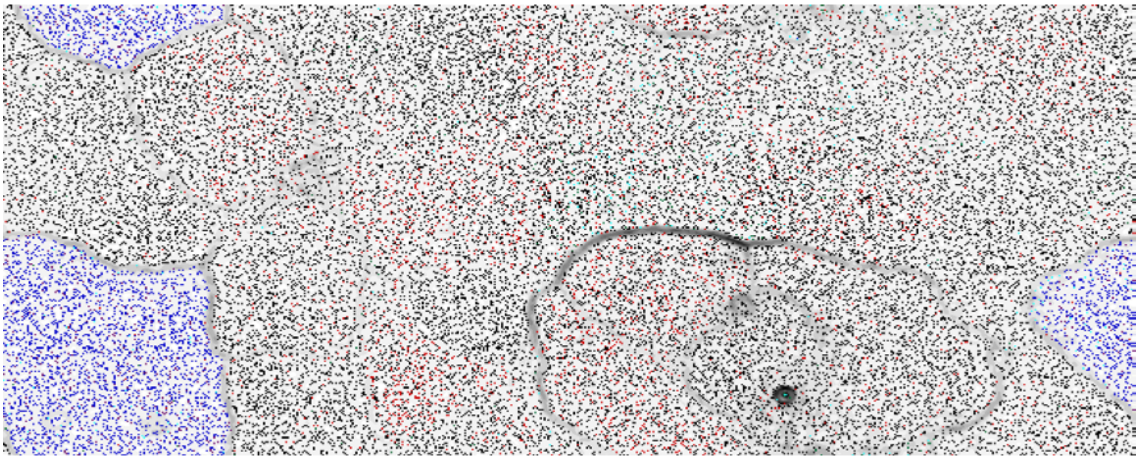

Supplementary Information 1-Fig. 11. ESOM map of the *P. atlantis* scaffolds from the second assembly. Each dot in the map correspond to one scaffold/scaffold window. Tetranucleotide frequency distances between neighbour scaffold/scaffold windows are represented with a white/black background gradient for smaller and larger distances, respectively. Color code: red for ‘Bact profile’, purple for ‘Euk profile Automated Bact +’, dark blue for ‘Euk profile Automated Bact -’, dark green for ‘Automated Bact +’, light blue ‘No data’ (see previous sections) purple for ‘Euk profile, Automated Bact+’ and black for contaminant contigs from the first assembly.

All except one ‘Euk profile’ scaffold windows clustered in the same region of the map (dark blue dots in Supplementary Information 1-Fig. 11), and hence all windows within that region were considered to belong to *P. atlantis* genome (light green dots Supplementary Information 1-Fig. 12). We inspected the taxonomic classification of the genes from the scaffolds within the *P. atlantis* region (colored in light green in Supplementary Information 1-Fig. 12) in order to detect potential misclassified contaminants. Five scaffolds that showed a suspicious pattern (few eukaryotic genes and some bacterial genes predicted) were finally kept because of their ratio of introns per gene (between 3-10.67 introns per gene). We consider the later a good marker of non-bacterial genes as the ratio of introns per gene in the contaminant contigs of the first assembly was 0.13 (10705 introns in 78682 predicted genes).

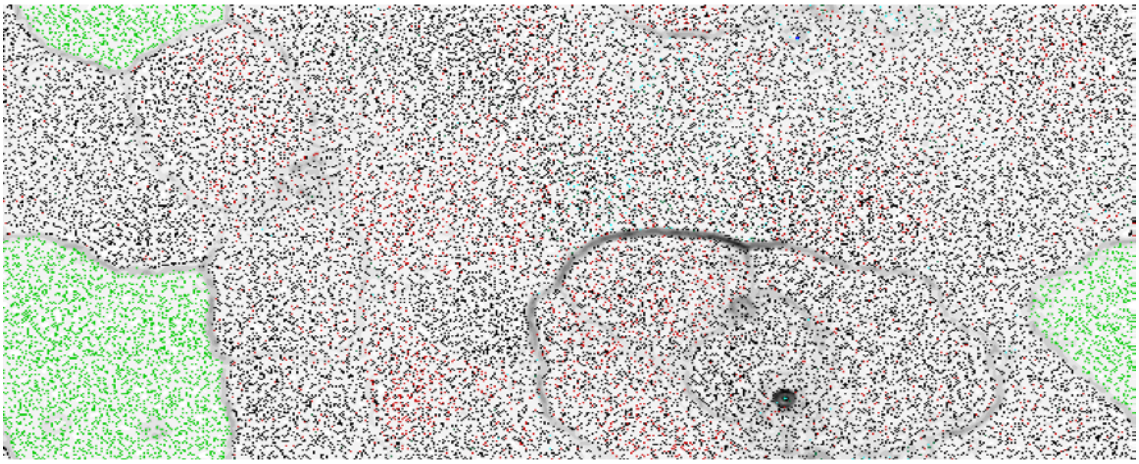

Supplementary Information 1-Fig. 12. ESOM map of the *P. atlantis* scaffolds from the second assembly, as in Supplementary Information 1-Fig. 11, but colored in light green the scaffold windows within the region of the map considered to correspond to *P. atlantis* genome.

To detect potential chimeric scaffolds, we inspected the ESOM class file for scaffolds with windows within the *P. atlantis* region and also outside this region. This revealed two potential chimeric scaffolds, a first one (24988 bp) was retained because it contained *bona fide* eukaryotic genes (verified by aligning them with NCBI nt) and the scaffold region corresponding to the window outside the *P. atlantis* region did not contain any bacterial gene predicted. The other potential chimera (8885 bp) was removed because of its short length and also because it did not include any *bona fide* eukaryotic gene.

Since scaffolds with less than 2000 of length were not considered in ESOM analyses, we excluded them from *Patl.gDNA.clean.v1.fasta*, with two exceptions. 1) We kept those aligning with >95% of identity to the set of *bona fide P. atlantis* transcripts (identified with the same approach as we did for *M. vibrans*), and 2) those containing annotations of eukaryotic genes. In both cases, scaffolds containing at least one bacterial or potential bacterial gene annotated were labeled as "_potentialcontaminant". As a final step to ensure that we did not include bacterial scaffolds in *Patl.gDNA.clean.v1.fasta*, we aligned them with NCBI nt [*BLASTn*: -task megablast; -evalue 1e-5] and the 4 scaffolds with less than 2000 of length that performed its best hit with a prokaryotic target (with >25% of average query coverage and > 50% of average identity) were also labeled as "_potentialcontaminant". In the end, 13 of 2555 scaffolds (9596 of 19259675 bp) were labeled as "_potentialcontaminant". We also labeled the scaffolds with putative mitochondrial and 18S ribosomal sequences as "_putative-mitochondrial" and "_ribosomal", respectively (3 putative mitochondrial scaffolds, 45473 bp in total).

We finally assessed completeness and contiguity of the decontaminated genome using a set of *de novo* assembled *bonafide* and non-redundant 5841 *P. atlantis* transcripts. 5461 and 5179 of these transcripts were complete and contiguous, respectively (5464 and 5147 in the case of the

first assembly contigs). We added complete and partial sequences from three contigs of the first assembly (2665 bp, labeled with the suffix "_fromdraftassembly") found to be incomplete in the second assembly.

3) *Pigoraptor* species (Filasterea, Opisthokonta)

3.1) Cultures, Cell cytometry, and DNA sequencing

As *M. vibrans* and *P. atlantis*, both *Pigoraptor* species grow in non-axenic condition with an undetermined diversity of contaminant bacteria but also with the eukaryotic contaminant *Parabodo caudatus*, used as prey. For both *Pigoraptor* species, we extracted DNA from a pooling of cells sorted by flow cytometry analyses (see *M. vibrans* section for FACS and DNA extraction protocols). Because the FACS protocol is designed to sort eukaryotic cells, we also expected contamination from *P. caudatus*. Hence, to *in silico* decontaminate both *Pigoraptor* libraries, we also obtained DNA from sorted cells from *P. caudatus* cultures. In total, we obtained 14.70 ng, 16.14 ng, and 11.73 ng for *P. vietnamica*, *P. chileana* and *P. caudatus* poolings, respectively. The PE libraries were prepared and sequenced each one in a 50% Illumina HiSeq 3000 lane using the sequencing kit HiSeq 3000/4000 chemistry (read insert sizes: 390, 410 and 410 bp for *P. vietnamica*, *P. chileana,* and *P. caudatus*, respectively; read lengths: 150 bp).

PE reads from both *Pigoraptor* species and from *P. caudatus* were preprocessed with *trimmomatic*, using the following parameters: *SLIDINGWINDOW:12:30 LEADING:30 ILLUMINACLIP:2:30:10 MINLEN:80*. As with *M. vibrans* and *P. atlantis*, TruSeq-PE related adapter sequences from *trimmomatic* were used as contaminant database for *ILLUMINACLIP*. The single and paired preprocessed reads were submitted to a read correction step using *SPAdes* [--only-error-correction].

3.2) Preliminary decontamination

The *P. caudatus* library was sequenced for decontamination purposes. Hence, we first assembled the *P. caudatus* reads using *SPAdes* with the metagenome mode. Then, we aligned the *P. vietnamica* and *P. chilena* reads with the *P. caudatus* scaffolds using *bowtie2*; in order to keep only those reads that did not align with *P. caudatus* scaffolds. In particular, we considered as potential *P. caudatus* contaminants all the unpaired reads (UP) that aligned, and all paired-end reads (PE) that aligned concordantly (i.e., both paired reads aligned to the same scaffold with an in-between distance close to the distance expected from the selected insert sizes). In order to discard false positive contaminant cases, we did not remove those reads that also aligned with the *C. owczarzaki* genome (the closest relative genome to *Pigoraptor* available), as they may correspond to highly conserved genomic regions. In total, excluding the reads that aligned with both genomes (0.16% UP and 0.016% PE of the reads that aligned with *P. caudatus* also aligned with *C. owczarzaki*), ~50% of *P. vietnamica* data (49.09% UP, 46.03% PE) aligned with *P.*

caudatus data (which includes *P. caudatus* and also uncertain Bacteria). In the case of *P. chileana*, the percentages of reads that aligned with *P. caudatus* but not with *C. owczarzaki* were 61.24% for UP, and 43.79% for PE (0.048% of UP and 0.0078% of PE reads that aligned with *P. caudatus* and also with *C. owczarzaki*).

3.3) First round of read decontamination (*P. vietnamica* and *P. chileana*)
For each *Pigoraptor* species, we next assembled the surviving reads using *metaSPAdes*, and the resulting contigs were evaluated for potential contamination as we did for *M. vibrans* and *P. atlantis* (i.e., adapters/vectors, 16S, 18S, and mitochondria; see 1.3.1).

In the case of *P. vietnamica*, we removed a total of 51 contigs (61268 bp) that aligned with > 50% of average query coverage and average identity with VecScreen sequences. We also found 72 contigs with potential 16S sequences. The contig with the highest assembly coverage (1980.97 cov) among those aligned by 18S sequences corresponded to the *P. vietnamica* 18S sequence. We also found a contig corresponding to the 18S of *P. caudatus*, but it had much less assembly coverage (57.45 cov) than the *P. vietnamica* 18S contig. This suggests that the read mapping-based decontamination approach reduced but did not fully removed all contaminant reads from *P. caudatus*. We also found one contig aligned by 18S sequences (633 bp, 9.30 cov) with 100% query coverage and identity to *H. sapiens* ribosomal sequences. The same contig aligned with lower identity to *C. owczarzaki* genome (77.40%) and with worse alignment metrics with fungal sequences on NCBI nt. Because we also found 1 contig with potential *H. sapiens* mitochondrial sequences (1429 bp, 6.05 cov), these altogether suggested the presence of low coverage *H. sapiens* contamination in *P. vietnamica* data.

In the case of *P. chileana*, we removed a total of 35 vector/adapter contigs (8140 bp), and we found 44 contigs with potential 16S sequences. As with *P. vietnamica*, we also found contigs corresponding to *P. caudatus* (679 bp, 343.19 cov; 230 bp, 26.92 cov) and possibly to *H. sapiens* (271 bp, 1.74 cov). The screening of mitochondrial sequences revealed two contigs likely containing the *P. chileana* mitochondria (12883 bp and 275.76 cov; 20715 bp and 273.77 cov), but also one potential contaminant from *P. caudatus* (20289 bp and 552.29 cov) and from *H. sapiens* (207 bp and 1.07 cov). These suggested as well the presence of potential *H. sapiens* low coverage contamination also in the *P. chileana* data.

In *M. vibrans* and *P. atlantis* data, the taxonomic classification of contigs into eukaryotic and bacterial allowed to distinguish between the regions of the ESOM map that corresponded to our organism of interest (the only eukaryote), and the regions that corresponded to bacterial contamination. This was not applicable for both *Pigoraptor* species given the presence of *P. caudatus* contamination. In both cases, we used an alternative approach that consisted in

identifying a set of *bona fide P. vietnamica, P. chileana* and *P. caudatus* scaffolds that were used to label the different regions of the map (see below).

For both *Pigoraptor* species, we aligned the proteins predicted from their transcriptomic data[28] with euk_prok_db [*BLASTp*: -evalue 1e-3 -task blastp-fast], and we kept those whose best hit was a *C. owczarzaki* protein (the closest relative to *Pigoraptor* in this database). We then aligned these proteins with the *P. caudatus* metagenome scaffolds and also with the contigs of the corresponding *Pigoraptor* species [*tBLASTn*: -evalue 1e-3]. Contigs encoding for those proteins that aligned with the *Pigoraptor* but not with the *P. caudatus* metagenome were considered as *bona fide Pigoraptor* contigs. We assumed the contig to which each protein aligned with the highest score as the encoding contig for that protein.

The set of *bona fide P. caudatus* scaffolds was defined in the following manner. We took the proteins from the transcriptomic data of *P. chileana* (from which *P. caudatus* contamination is expected) that did not align with prokaryotic proteins in the *BLASTp* search with euk_prok_db (see previous paragraph). We then aligned these likely eukaryotic proteins with the *P. caudatus* metagenome [*tBLASTn*: -evalue 1e-49], and we keep as *bona fide P. caudatus* scaffolds those in which at least one of these proteins performed aligned with the highest score.

For each *Pigoraptor* species, contigs > 2000 bp were submitted to ESOM analyses, splitting contigs larger than 7999 bp into contig windows of 4000 bp (SM Figs 13-14 and 15-16 correspond to the ESOM maps of *P. vietnamica* and *P. chileana*, respectively). Contigs were colored in ESOM maps according to the categories to which they belong: '16S', '18S', 'mitochondria', '*bona fide Pigoraptor*', 'Others'. In both ESOM maps we also incorporated the *bona fide P. caudatus* scaffolds (see above) in order to detect which regions of both *Pigoraptor* assemblies corresponded to *P. caudatus* contamination. In the case of *P. vietnamica* data, all *bona fide P. vietnamica* contigs (Supplementary Information 1-Fig. 13) were found within a region that did not include any *bona fide P. caudatus* contig (black contigs in Supplementary Information 1-Fig. 13). Accordingly, all contigs with a window within that region were included in the 'Non-contaminant' set (colored in orange in Supplementary Information 1-Fig. 14). In contrast, all contigs in the map without windows within this region were included in the 'Contaminant' set (excluding the putative mitochondrial and 18S contigs of *P. vietnamica*).

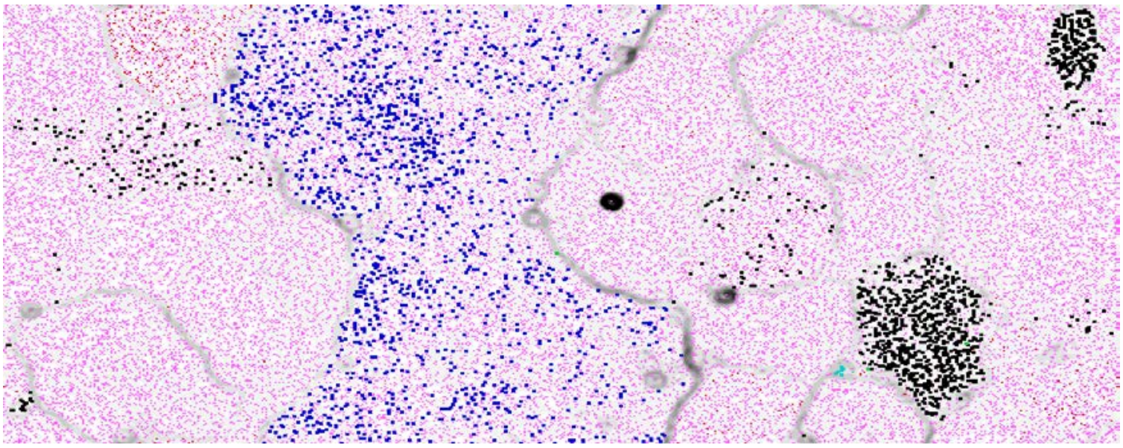

Supplementary Information 1-Fig. 13. ESOM map of the *P. vietnamica* contigs from the first assembly. Each dot in the map correspond to one contig/contig window, which are colored according to the category to which was classified. Category color code: red for ‘16S’, green for ‘18S’, light blue for ‘mitochondria’, dark blue for ‘*bona fide P. vietnamica*’, pink for ‘Others’. We also incorporated the *bona fide P. caudatus scaffolds* (from the *P. caudatus* metagenome, colored in black) in order to detect which regions of the *P. vietnamica* assembly correspond to the *P. caudatus* genome. Tetranucleotide frequency distances between neighbour contig/contig windows are represented with a white/black background gradient for smaller and larger distances, respectively.

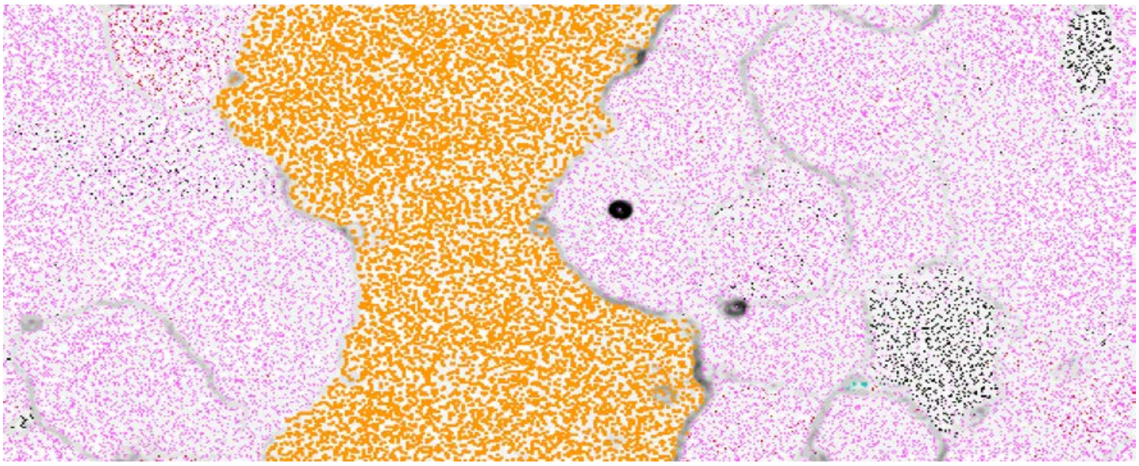

Supplementary Information 1-Fig. 14. ESOM map of the *P. vietnamica* contigs from the first assembly, as in Supplementary Information 1-Fig. 13, but colored in orange the contigs windows within the region of the map considered to correspond to *P. vietnamica* genome.

In the case of *P. chileana*, however, whereas most of the *bona fide P. chileana* contig windows were proximal in the map (dark blue dots in Supplementary Information 1-Fig. 15), others were in a different region (see top and bottom regions in the middle of the map), which also included *bona fide P. caudatus* scaffolds (black dots in Supplementary Information 1-Fig. 15). All these other windows were from the same contig, suggesting that is was a *P. caudatus* contig misclassified as *P. chileana*. Consequently, only the region of the map with the majority of dark blue dots was considered as non-contaminant, and hence all contigs with windows within or surrounding this

region were considered included in this set (colored in orange in Supplementary Information 1-Fig. 16). All contigs in the map without windows within the selected region were included in the 'Contaminant' set.

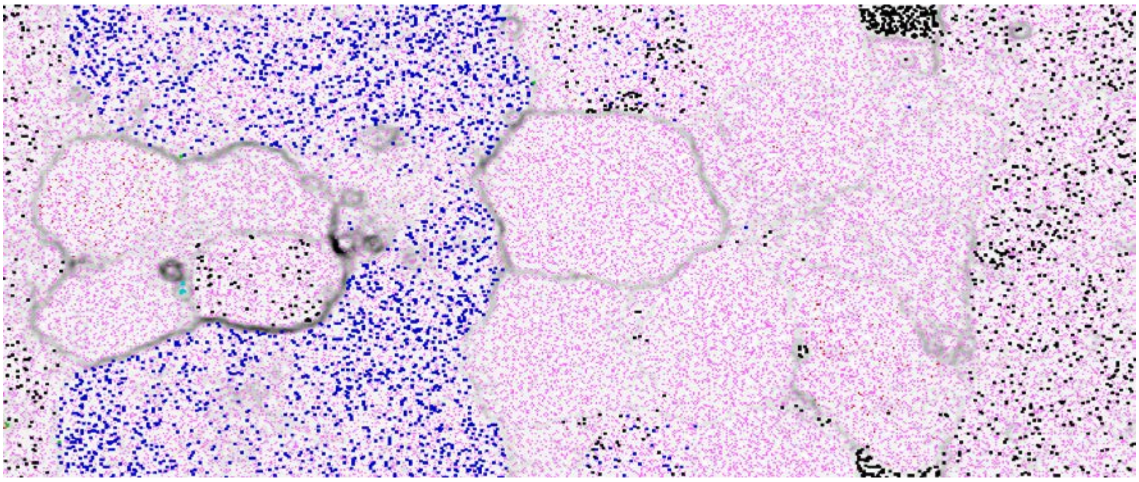

Supplementary Information 1-Fig. 15. ESOM map of the *P. chileana* contigs from the first assembly. Each dot in the map correspond to one contig/contig window, which are colored according to the category to which was classified. Category color code: red for '16S', green for '18S', light blue for 'mitochondria', dark blue for '*bona fide P. chileana*', pink for 'Others'. We also incorporated the *bona fide P. caudatus scaffolds* (from the *P. caudatus* metagenome, colored in black) in order to detect which regions of the *P. chileana* assembly correspond to the *P. caudatus* genome. Tetranucleotide frequency distances between neighbour contig/contig windows are represented with a white/black background gradient for smaller and larger distances, respectively.

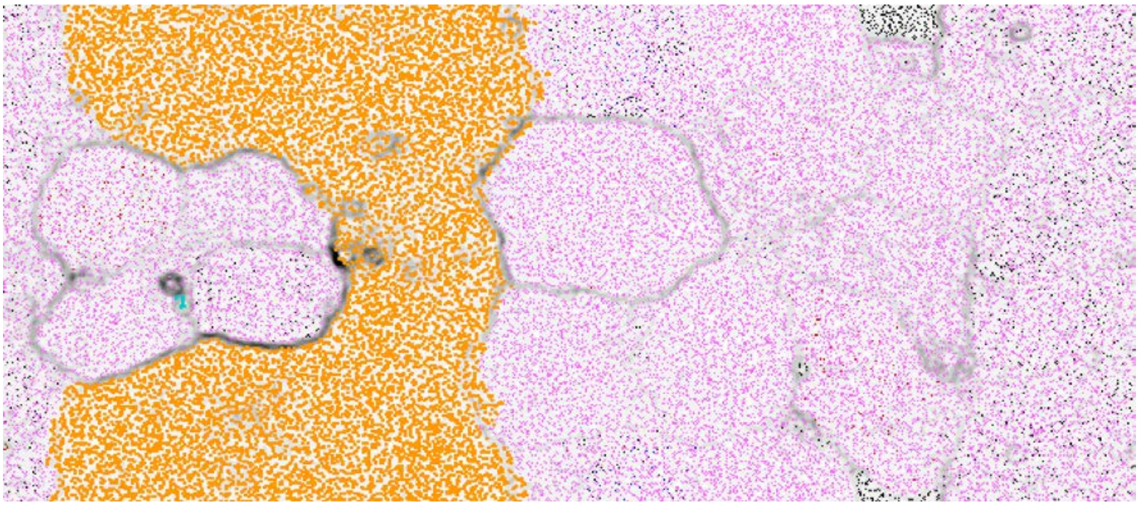

Supplementary Information 1-Fig. 16. ESOM map of the *P. chileana* contigs from the first assembly, as in Supplementary Information 1-Fig. 15, but colored in orange the contigs windows within the region of the map considered to correspond to *P. chileana* genome.

For *M. vibrans* and *P. atlantis*, we directly used the ‘Contaminant’ and ‘Non-contaminant’ contig sets to remove the contaminant reads and then do a second assembly. For both *Pigoraptor* species, before doing this, we first removed potential contaminant reads from *H. sapiens*, as contamination was suggested by the 18S and mitochondrial analyses. For that, we aligned the reads used in the first assembly with the *H. sapiens* genome [*bowtie2*]. As we did for the alignments with the *P. caudatus* metagenome (see 3.2), we did not consider the reads that also aligned with *C. owczarzaki* genome, as they may correspond to conserved regions. For *P. vietnamica*, the 1.80% of UP -unpaired- and 1.48% of PE -paired- reads aligned with *H. sapiens* but not with *C. owczarzaki* (1.71% of UP and 0.06% of PE of the reads that aligned to *H. sapiens* also aligned with *C. owczarzaki*). For *P. chileana*, the 0.20% of UP and 0.07% of PE reads aligned with *H. sapiens* but not with *C. owczarzaki* (3.03% of UP and 0.74% of PE of the reads that aligned with *H. sapiens* also aligned with *C. owczarzaki*). These results confirmed the hypothesized *H. sapiens* contamination, quantitatively lower in *P. chileana* than in *P. vietnamica*.

3.4) <u>Second round of read decontamination (*P. vietnamica*)</u>
We then also excluded the *P. vietnamica* reads that aligned with the ‘Contaminant’ and ‘Non-contaminant’ post-ESOM sets (see 1.4). 30.08% of the reads aligned with the ‘Contaminant’ but not with the *P. vietnamica* (i.e., non-contaminant) dataset. The second assembly of *P. vietnamica* was done with *SPAdes*, with the metagenome mode disabled, and using the following k-mers: 51, 71, 93, 115 [--careful --cov-cutoff auto]. The average coverage of the 115-mer assembly was 42.21.

Then, as with the first assembly, we screened the assembled scaffolds for potential contamination of adapter/vector sequences, and also for 18S, 16S, mitochondrial sequences and *bona fide P. vietnamica* scaffolds (see 3.3). We removed 7 scaffolds of short length (4143 bp) because they aligned with vector sequences along most of their sequence. We found 37 scaffolds potentially containing 16S ribosomal genes. We did not find any scaffold with *H. sapiens* 18S or mitochondrial sequences, suggesting that we successfully removed the *H. sapiens* contamination. We also found a scaffold with the putative *P. caudatus* 18S gene (13903 bp, 17.53 cov), but it had less assembly coverage than in the first assembly (57.45 cov).

Scaffolds with > 2000 bp were submitted to ESOM analyses, splitting scaffolds larger than 7999 bp into windows of 4000 bp. Scaffolds were colored in ESOM maps according to the categories to which they belong: ‘16S’, ‘18S’, ‘mitochondria’, ‘*bona fide P. vietnamica*’, ‘Others’. We also incorporated the *bona fide P. caudatus* fragments (see 3.3). Contrary to previous results, most of the map corresponds to the *bona fide P. vietnamica* contigs region (see dark blue dots in Supplementary Information 1-Fig. 17), indicating that we successfully removed a substantial fraction of contamination during the decontamination of the first assembly. However, the lack of a clear white/black background gradient separating the *P. vietnamica* and the *P. caudatus* regions

(see black dots) left uncertainty surrounding the scaffolds found in-between both regions. From ESOM results, we preliminary classified scaffolds into ‘Non-contaminant’, ‘Contaminant’ and ‘Uncertain’ (see orange dots, brown dots and other color dots, respectively, in Supplementary Information 1-Fig. 18). For those scaffolds with windows in more than one category, we used the following criteria: (i) Scaffolds with at least one ‘Uncertain’ window were classified as ‘Uncertain’. (ii) Scaffolds with windows in both ‘Non-contaminant’ and ‘Contaminant’ were classified as ‘Uncertain’. Finally, scaffolds not included in ESOM analyses (i.e., < 2000 bp) were classified as ‘<2000’ (a fourth category).

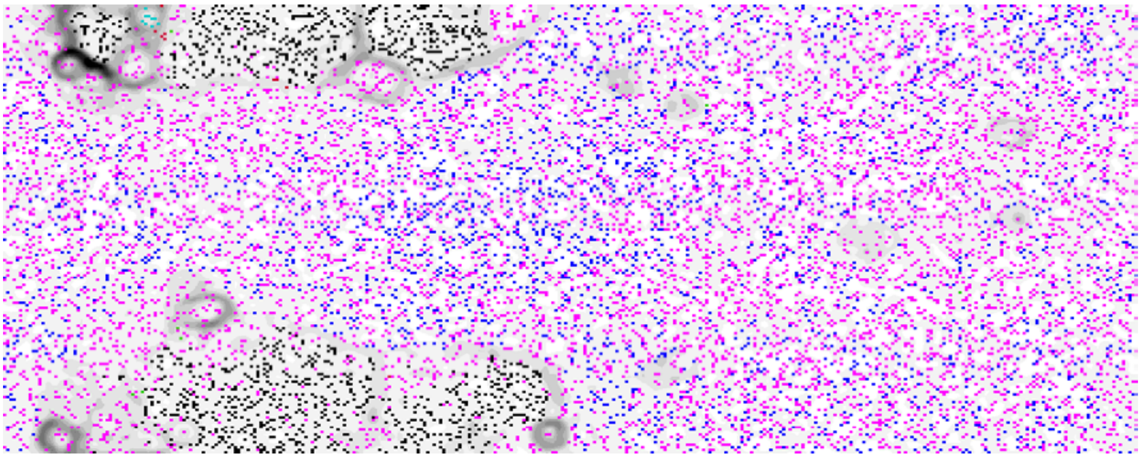

Supplementary Information 1-Fig. 17. ESOM map of the *P. vietnamica* scaffolds from the second assembly. Each dot in the map correspond to one scaffold/scaffold window, which are colored according to the category to which was classified. Category color code: red for ‘16S’, green for ‘18S’, light blue for ‘mitochondria’, dark blue for ‘*bona fide P. vietnamica*, pink for ‘Others’. We also incorporated the *bona fide P. caudatus* fragments (from the *P. caudatus* metagenome, colored in black) in order to detect which regions of the *P. vietnamica* assembly correspond to the *P. caudatus* genome. Tetranucleotide frequency distances between neighbour scaffold/scaffold windows are represented with a white/black background gradient for smaller and larger distances, respectively.

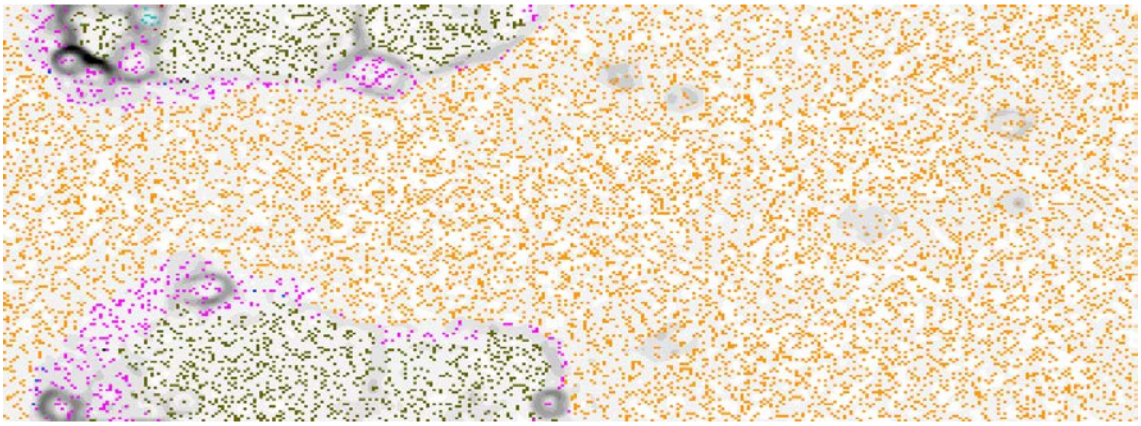

Supplementary Information 1-Fig. 18. ESOM map of the *P. vietnamica* scaffolds from the second assembly, as in Supplementary Information 1-Fig. 17, but colored in orange the scaffold windows within the

region of the map likely corresponding to *P. vietnamica* genome, in brown the region likely corresponding to contaminant fragments. Other scaffolds were considered as ‘Uncertain’.

With *M. vibrans* and *P. atlantis*, we determined which scaffolds corresponded to our genome of interest directly from ESOM results. In this case, given the lack of resolution in-between some *P. vietnamica* and *P. caudatus* regions, the preliminary classification from ESOM was redefined by taking into account the following scaffold features: (1) assembly coverage, (2) taxonomic profiling, (3) average number of introns per eukaryotic gene and per bacterial gene, (4) eukaryotic taxonomic profiling, (5) total query coverage in alignments with *P. caudatus* assembly and (6) total query coverage in alignments with NCBI nt.

(1) The assembly coverage was obtained from the scaffold names (computed by *SPAdes*).
(2) The taxonomic profiling was obtained using the indirect taxonomic classification approach (explained in *M. vibrans* section). First scaffolds were preliminary annotated with *BRAKER1*, and then we counted the number of eukaryotic genes (‘E’+‘PE’) and bacterial genes (‘B’+‘PB’) per scaffold. The RNA-seq reads required for the *BRAKER1* annotation pipeline were kindly provided by Elisabeth Hehenberger[28].
(3) The average number of introns per eukaryotic gene and per bacterial gene were computed from the *BRAKER1* GFF3 output file.
(4) The predicted eukaryotic protein sequences were aligned with a comprehensive local eukaryotic database [*BLASTp*: -evalue 1e-3], and the corresponding genes were later classified into five taxonomic categories: (i) ‘F’ if the best hit was from Filasterea, (ii) ‘E’ if the best hit was from Excavata, (iii) ‘M’ if the best hit was from Metazoa, (iv) ‘O’ if the best hit was from Opisthokonta but not from Filasterea and Metazoa and (v) ‘D’ if the best hit was from any other taxonomic group.
(5) Scaffolds were aligned with the *P. caudatus* metagenome [*BLASTn*: -evalue 1e-3], and then we computed the total query coverage of every scaffold by dividing the number of positions that aligned with a database sequence by the length of that scaffold.
(6) The same approach as (5), but using the NCBI nt database.

We expected from *P. vietnamica* scaffolds to show (1) higher assembly coverage values than *P. caudatus* or other contaminant scaffolds. The median assembly coverage values for ‘Non-contaminant’ ESOM contigs were 49.42, clearly higher than those for the ‘Contaminant’ ESOM contigs (3.02); (2) positive E+PE/B+PB ratios; (3) similar number of introns in eukaryotic and in bacterial genes (if any); (4) more F, M, O genes than E or D; (5) spurious query coverage values in the alignment with *P. caudatus* assembly and (6) with the NCBI nt database.

Compared to *P. vietnamica* scaffolds, we expected from *P. caudatus* scaffolds (1) low assembly coverage values; (4) more E or D genes than F, M, O and (5) high query coverage values in the alignments with *P. caudatus* assembly.

From bacterial contaminant scaffolds, we expected (1) low assembly coverage values; (2) negative E+PE/B+PB ratios; (3) none or very few introns in genes (false positives); (6) possibly high query coverage values in the alignment with NCBI nt (if the corresponding species is represented in the database).

From potential *H. sapiens* contaminant scaffolds (most likely excluded in previous decontaminations), we expected (1) low assembly coverage values; (4) more M than other genes, and few or none F genes; (6) possibly high query coverage values in the alignment with NCBI nt database (if the corresponding sequence is represented in the database).

Based on the above-mentioned expectations, scaffolds were finally classified into '*P. vietnamica*', 'Potential contaminant' and 'Contaminant' using a manual decision-tree approach (with the exception of the putative mitochondrial scaffolds, which were directly classified into *P. vietnamica*). We explored different combinations of nested conditional *If/Else* statements, which were subsequently improved based on the observed classification outcomes (i.e., after every decision-tree, we evaluated if the parameters of the scaffolds classified within each category disagreed with any of the above-mentioned expectations, and we modified the algorithm accordingly to overcome the observed disagreements). See below the custom-made final decision-tree for *P. vietnamica*.

Abbreviations:
cov: assembly coverage value
qcPCAU: total query coverage of a scaffold in the alignments with *P. caudatus* scaffolds.
qcNCBI: total query coverage of a scaffold in the alignments with NCBI nt.
pY: scaffolds preliminary classified as 'Non-contaminant' in ESOM analyses
pD: scaffolds preliminary classified as 'Uncertain' in ESOM analyses
pN: scaffolds preliminary classified as 'Contaminant' in ESOM analyses
less2000: scaffold preliminary classified as '<2000', not included in ESOM analyses
Y: scaffolds classified as '*P. vietnamica*'
D: scaffolds classified as 'Potential contaminant'
N: scaffolds classified as 'Contaminant'
Bact: B+PB
Euk: E+PE
B_introns: average number of introns in Bacterial genes
E_introns: average number of introns in Eukaryotic genes
Euk_F: eukaryotic genes taxonomically classified as F

Euk_M: eukaryotic genes taxonomically classified as M

Euk_O: eukaryotic genes taxonomically classified as O

Euk_E: eukaryotic genes taxonomically classified as E

Euk_D: eukaryotic genes taxonomically classified as D

Decision-tree for *P. vietnamica*:

```
If qcPCAU or If qcNCBI >= 50%: --> N
Else:
    If pY: --> by default Y, but:
        If cov <= 25:
            If Bact > Euk:
                If B_introns = 0: --> D
            If Euk = 0 --> D
            If Euk != 0:
                If Euk_F = 0: --> D
                If Euk_F > 0:
                    If Euk_F < Euk_M: --> D
                    If Euk_E > 0: --> D
                    If (Euk_E+Euk_D) > (Euk_O+Euk_M+Euk_F): --> D
        If cov > 25:
            If Bact > Euk:
                If B_introns = 0: --> D
            If E > 0:
                If Euk_E >= Euk_F:
                    If qcPCAU > 5%: --> D
            Else: --> Y
    If pN:
        If Euk > Bact:
            If (Euk_O+Euk_M+Euk_F+Euk_D) > Euk_E:
                If Bact > 0:
                    If B_introns > 0: --> D
                If Bact = 0: --> D
        Else: --> N

    If pD:
        If cov <= 25: --> by default N, but:
            If (Euk_O+Euk_F+Euk_M) >= (Euk_E+Euk_D) or If Euk_F > 0: --> D
            If Euk < Bact or If Euk = 0: --> N
        If cov > 25: --> by default Y, but:
            If Euk < Bact:
                If Euk_F > 0:
                    If B_introns > 0: --> Y
                    If B_introns < 0: --> D
```

```
            or If B_introns > 0: --> D
            Else: --> N
        If Euk >= Bact:
            If Euk_E > (Euk_O+Euk_F+Euk_M): --> D
            Else: --> Y

If less2000: --> by default N, but:
    If cov <= 25:
        If Euk_F > (Euk_O+Euk_M+Euk_E+Euk_D): --> D
    If cov > 25:
        If Euk < Bact:
            If (Euk_O+Euk_F+Euk_M) >= (Euk_E+Euk_D):
                If B_introns > 0: --> Y
                Else: --> D
        If Bact = 0:
            If (Euk_O+Euk_F+Euk_M) >= (Euk_E+Euk_D): --> Y
            Else: --> D
        If Bact > 0 and Bact < Euk:
            If (Euk_O+Euk_F+Euk_M) >= (Euk_E+Euk_D):
                If B_introns > 0: --> Y
                Else: --> D
```

We observed that scaffolds with > 25 assembly coverage tend to be larger, have many eukaryotic and few bacterial genes, and introns in bacterial genes. Not all genes from the scaffolds classified as '*P. vietnamica*' performed best hit with *C. owczarzaki* or *M. vibrans* proteins, which could be expected given the sequence divergence shown from other filastreans respect to the Pigoraptor clade[28]. As expected, many scaffolds classified as '*P. vietnamica*' and 'Contaminant' were already preliminary classified into 'Non-contaminant' and 'Contaminant' after ESOM analyses, respectively. For the scaffolds preliminary classified as 'Uncertain' and '<2000', the most determinant parameters were eukaryotic taxonomic profiling and assembly coverage.

Scaffolds classified as '*P. vietnamica*' (3390 scaffolds, 42153970 bp) and as 'Uncertain' (601 scaffolds, 879351) by the decision tree were included in the *Pvie.gDNA.clean.v1.fasta* file, with the name of the 'Uncertain' scaffolds being labeled with the suffix '_potentialcontaminant'. The scaffolds with 18S and mitochondrial sequences (1 mitochondrial scaffold, 39208 bp) were labeled as 'ribosomal' and '_mitochondrial', respectively.

3.5) <u>Second round of read decontamination (*P. chileana*)</u>

After removing *H. sapiens* contamination, we excluded the reads that aligned with the 'Contaminant' and 'Non-contaminant' post-ESOM sets (see 3.3.3). 66.63% of the reads aligned with the 'Contaminant' but not with the *P. chileana* ('Non-contaminant') dataset, more than twice

than in *P. vietnamica* (30.08%). Because the remaining contaminant reads did not allow an assembly with enough coverage, we sequenced an extra Illumina HiSeq 2500 lane using the sequencing kit HiSeq v4 chemistry (insert size: 410 bp, read length: 125 bp). Reads from this extra library were preprocessed, corrected and also decontaminated as with the first library (i.e., we removed those reads aligning either with *P. caudatus* scaffolds or with *H. sapiens* but not with *C. owczarzaki*; as well as those reads aligning to the 'Contaminant' but not to the 'Non-contaminant' sets).

The second assembly of *P. chileana* was performed with both libraries, first using *SPAdes* without the metagenome mode. However, the assembly ended with the warning "Failed to determine erroneous kmer threshold", which suggested uneven coverage problems occurred because of substantial amounts of persistent contamination. We found that the coverage problem lied on the reads corresponding to the first assembly contigs with <2000 bp length, which were not included in the ESOM decontamination analyses. To avoid potential pitfalls in the assembly related to uneven coverage, the second assembly was finally performed using the metagenome mode, with the following k-mers: 21, 33, 45, 57. The average coverage of the 57-mer assembly was 38.40.

Then, as with the first assembly, we checked for potential remaining adapter/vector sequences, and also for 18S, 16S, mitochondrial sequences and *bona fide P. chileana* scaffolds. We removed other 7 scaffolds of short length (21112 bp) because they aligned against vector sequences along most of their sequence. We found 64 scaffolds potentially containing 16S ribosomal genes. We did not find any scaffold with *H. sapiens* 18S or mitochondrial sequences, suggesting that we successfully removed the *H. sapiens* contamination. Despite we found putative *P. caudatus* 18S and mitochondrial scaffolds, they had very short lengths (<637 bp) and lower assembly coverage values than the 18S and mitochondrial *P. chileana* scaffolds.

Scaffolds with >2000 bp were submitted to ESOM analyses, splitting scaffolds larger than 7999 bp into windows of 4000 bp. Scaffolds were colored in ESOM maps according to the categories to which they belong: '16S', '18S', 'mitochondria', '*bona fide P. chileana*, 'Others'. We also incorporated the *bona fide P. caudatus* scaffolds (from the *P. caudatus* metagenome). The *bona fide P. chileana* contigs covered an extensive region, approximately half of the map (see dark blue dots in Supplementary Information 1-Fig. 19). However, as with the ESOM from *P. vietnamica* second assembly, a clear white/black background gradient separating the *P. vietnamica* and the *P. caudatus* regions (see black dots) was missing. Moreover, the bottom left subregion of the putative *P. vietnamica* region had less blue dots compared to the other parts of the region, which suggested the presence of contaminant scaffolds within. Because of this, we did as for *P. chileana*, and we used again ESOM results to preliminary classify scaffolds into 'Non-contaminant', Uncertain' and 'Contaminant' (see orange dots, brown dots and other color dots, respectively, in Supplementary Information 1-Fig. 20).

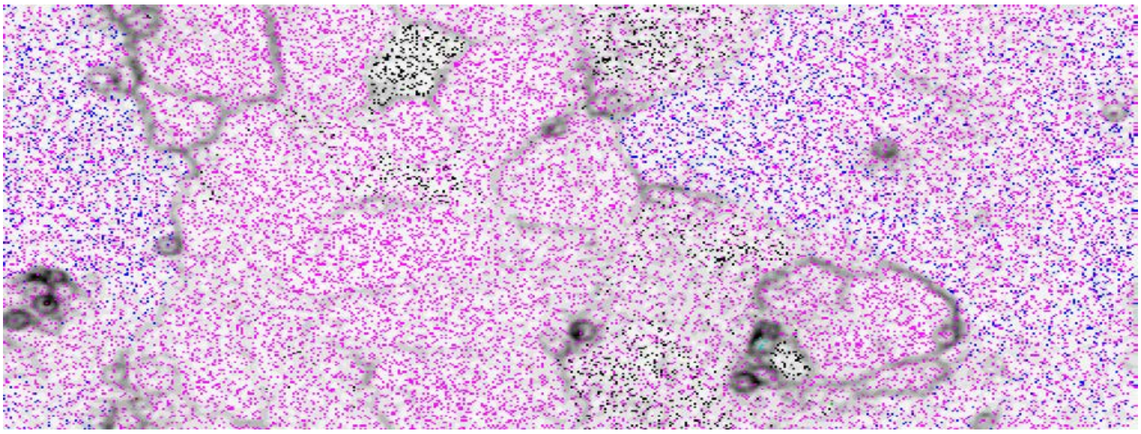

Supplementary Information 1-Fig. 19. ESOM map of the *P. chileana* scaffolds from the second assembly. Each dot in the map correspond to one scaffold/scaffold window, which are colored according to the category to which was classified. Category color code: red for '16S', green for '18S', light blue for 'mitochondria', dark blue for '*bona fide P. chileana*', pink for 'Others'. We also incorporated the *bona fide P. caudatus scaffolds* (from the *P. caudatus* metagenome, colored in black) in order to detect which regions of the *P. chileana* assembly correspond to the *P. caudatus* genome. Tetranucleotide frequency distances between neighbour scaffold/scaffold windows are represented with a white/black background gradient for smaller and larger distances, respectively.

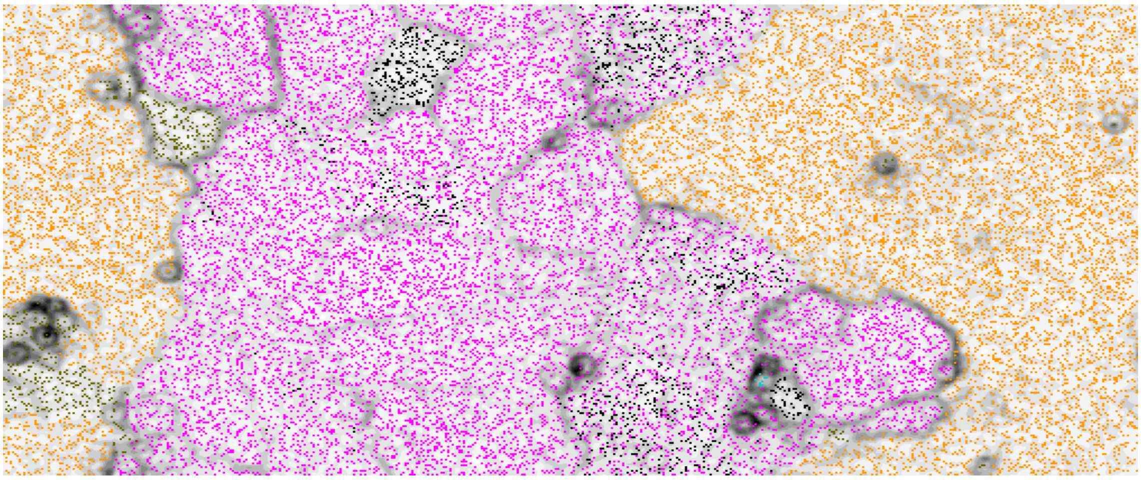

Supplementary Information 1-Fig. 20. ESOM map of the *P. chileana* scaffolds from the second assembly, as in Supplementary Information 1-Fig. 19, but colored in orange the scaffold windows within the region of the map likely corresponding to *P. chileana* genome ('Non-contaminant' set), and in brown the region likely corresponding to uncertain fragments.

As with *P. vietnamica*, 'Non-contaminant' scaffolds showed clearly higher assembly coverage values than 'Contaminant' scaffolds (median values were 57.02 and 4.79, respectively). For a final classification of scaffolds into '*P. chileana*, 'Potential contaminant' and 'Contaminant' we used a similar custom-made decision-tree approach as we did for *P. vietnamica* (see below).

Abbreviations:

cov: assembly coverage value

qcPCAU: total query coverage of a scaffold in the alignments with *P. caudatus* scaffolds.

qcNCBI: total query coverage of a scaffold in the alignments with NCBI nt.

pY: scaffolds preliminary classified as 'Non-contaminant' in ESOM analyses

pD: scaffolds preliminary classified as 'Uncertain' in ESOM analyses

pN: scaffolds preliminary classified as 'Contaminant' in ESOM analyses

less2000: scaffold preliminary classified as '<2000', not included in ESOM analyses

Y: scaffolds classified as '*P. chileana*'

D: scaffolds classified as 'Potential contaminant'

N: scaffolds classified as 'Contaminant'

Bact: B+PB; Euk: E+PE

B_introns: average number of introns in Bacterial genes

E_introns: average number of introns in Eukaryotic genes

Euk_F: eukaryotic genes taxonomically classified as F

Euk_M: eukaryotic genes taxonomically classified as M

Euk_O: eukaryotic genes taxonomically classified as O

Euk_E: eukaryotic genes taxonomically classified as E

Euk_D: eukaryotic genes taxonomically classified as D

Decision-tree for *P. chileana*, second round of decontamination:

If qcPCAU or If qcNCBI >= 50%: --> N

Else:

- If pY: --> by default Y, but:
  - If cov <= 25:
    - If Bact > Euk:
      - If B_introns = 0: --> D
      - If B_introns > 0: --> Y
    - If Euk = 0: --> D
    - If Euk > 0:
      - If Euk_F = 0: --> D
      - If Euk_F < Euk_M: --> D
      - If Euk_E > 0:
        - If (Euk_E+Euk_D) >= (Euk_F+Euk_M+Euk_O): --> D
  - If cov > 25:
    - If Bact > Euk:
      - If B_introns = 0: --> D
    - If Euk_E > 0:
      - If Euk_E >= Euk_F:
        - If qcPCAU >= 5%: --> D

- If pN: --> by default N, but:

```
        If Euk > Bact:
                If (Euk_F+Euk_M+Euk_O+Euk_D) > Euk_E:
                        If B_introns > 0: --> D

If pD: --> by default N, but:
        If cov <= 25:
                If Euk > 0:
                        If (Euk_M+Euk_F+Euk_O) >= (Euk_D+Euk_E): --> D
                        If Euk_F > 1: --> D
                If Euk < Bact: --> N
                If Euk = 0: --> N
        If cov > 25:
                If Euk < Bact:
                        If Euk_F > 0:
                                If B_introns > 0: --> Y
                                Else: --> D
                        Else: --> N
                If Euk >= Bact:
                        If Euk > 0:
                                If Euk_E > (Euk_F+Euk_M+Euk_O): --> D
                                Else: --> Y

If less2000:
        If cov <= 25:
                If Euk_F > (Euk_M+Euk_O+Euk_D+Euk_E): --> D
        If cov > 25:
                If Bact = 0:
                        If (Euk_M+Euk_O+Euk_F) > (Euk_D+Euk_E): --> Y
                        Else: --> D
                If Euk < Bact:
                        If (Euk_M+Euk_O+Euk_F) > (Euk_D+Euk_E):
                                If B_introns > 0: --> Y
                                Else: --> D
                If Bact > 1 and Euk >= Bact:
                        If (Euk_M+Euk_O+Euk_F) > (Euk_D+Euk_E): --> D
                                If B_introns > 0: --> Y
                                Else: --> D
```

With *P. vietnamica*, the post-decision tree classification of the second assembly was used to determine which scaffolds were included in the *Pvie.gDNA.clean.v1.fasta* file. However, with *P. chileana*, the uneven coverage problems limited the quality of the second assembly. We hence

used the classification results from this second assembly to perform a third read decontamination step to allow a third assembly without uneven coverage problems.

3.6) Third round of read decontamination (*P. chileana*)

We discarded the reads that aligned with the ‘Contaminant’ but not with the ‘Uncertain’ or the ‘*bona fide P. chileana*’ scaffolds, this corresponding to the 56.69% of the reads [*bowtie2*]. For PE reads, we only removed them if both paired reads satisfied this criteria. We assembled the de-contaminated reads of *P. chileana* using *SPAdes* without the metagenome mode [-k 35,49,63,75 --careful --cov-cutoff auto]. In contrast with the second assembly, this third assembly ended without warning uneven coverage problems, suggesting that our decision tree classification approach removed substantial contamination issues. The average coverage of the 75-mer assembly was 40.55. We found 21 scaffolds with potential 16S sequences, and 3 scaffolds corresponding to vector/adapter sequences were removed (957 bp). We did not find any potential 18S or mitochondrial *H. sapiens* sequence. We also identified a set of *bona fide P. chileana* scaffolds (see 3.3).

Scaffolds with >2000 bp were submitted to ESOM analyses, splitting scaffolds larger than 7999 bp into windows of 4000 bp. Scaffolds were colored in ESOM maps according to the categories to which they belong: ‘16S’, ‘18S’, ‘mitochondria’, ‘*bona fide P. chileana*, ‘Others’, ‘*bona fide P. caudatus* scaffolds’ (from the *P. caudatus* metagenome). We also incorporated the ‘Contaminant’ scaffolds from the second assembly. As occurred in the ESOM analyses of the second decontamination rounds of both *Pigoraptor* species, there were some regions without a clear white/black background gradient separating the *P. vietnamica* (see blue dots in Supplementary Information 1-Fig. 21) and the *P. caudatus* and contaminant regions regions (see black and red dots).

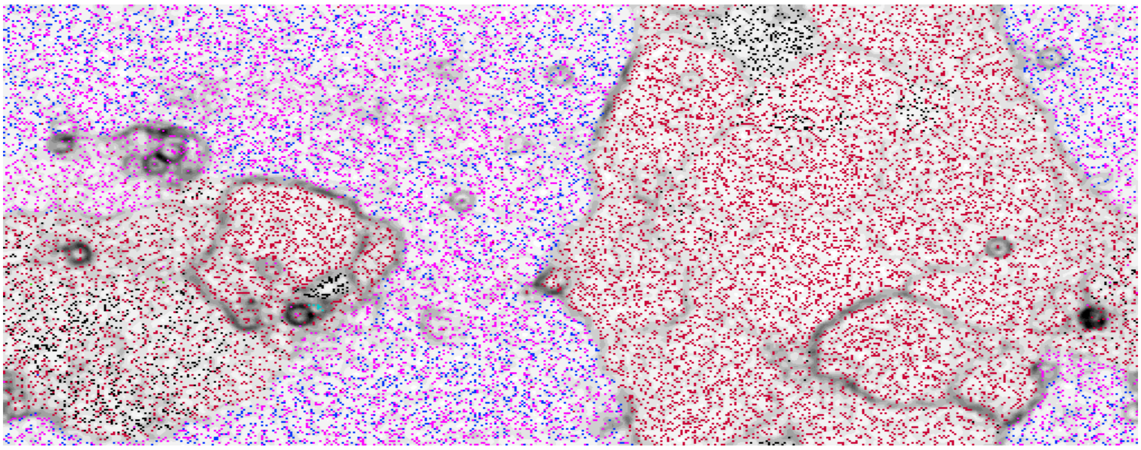

Supplementary Information 1-Fig. 21.ESOM map of the *P. chileana* scaffolds from the second assembly. Each dot in the map correspond to one scaffold/scaffold window, which are colored according to the category to which was classified. Category color code: red for ‘16S’, green for ‘18S’, light blue for ‘mitochondria’, dark

blue for ‘*bona fide P. chileana*’, pink for ‘Others’. We also incorporated the ‘Contaminant’ scaffolds from the second assembly and the *bona fide P. caudatus scaffolds* from the *P. caudatus* metagenome (colored in red in black, respectively). Tetranucleotide frequency distances between neighbour scaffold/scaffold windows are represented with a white/black background gradient for smaller and larger distances, respectively.

We hence used again ESOM results to preliminary classify scaffolds into ‘Non-contaminant’, ‘Contaminant’ and ‘Uncertain’ (see orange dots, brown dots and other color dots, respectively, in Supplementary Information 1-Fig. 22).

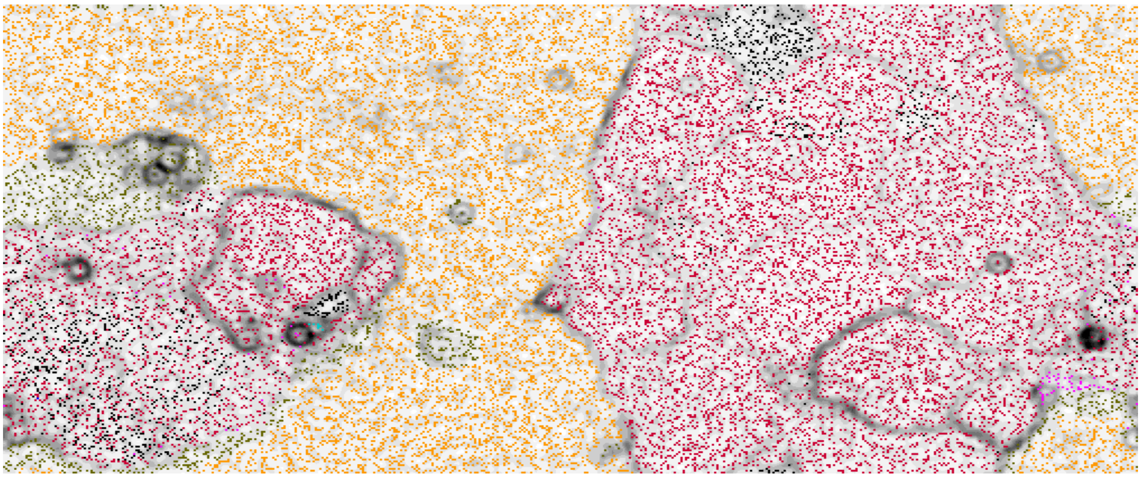

Supplementary Information 1-Fig. 22. ESOM map of the *P. chileana* scaffolds from the third assembly, as in Supplementary Information 1-Fig. 21, but colored in orange the scaffold windows within the region of the map likely corresponding to *P. chileana* genome, and in brown the region likely corresponding to uncertain fragments.

For a final classification of scaffolds into ‘*P. chileana*’, ‘Potential contaminant’ and ‘Contaminant’, we used a custom-made decision-tree approach as in the second assembly.

Abbreviations:
cov: assembly coverage value
qcPCAU: total query coverage of a scaffold in the alignments with *P. caudatus* scaffolds.
qcNCBI: total query coverage of a scaffold in the alignments with NCBI nt.
pY: scaffolds preliminary classified as 'Non-contaminant' in ESOM analyses
pD: scaffolds preliminary classified as 'Uncertain' in ESOM analyses
pN: scaffolds preliminary classified as 'Contaminant' in ESOM analyses
less2000: scaffold preliminary classified as '<2000', not included in ESOM analyses
Y: scaffolds classified as '*P. chileana*'
D: scaffolds classified as 'Potential contaminant'
N: scaffolds classified as 'Contaminant'
Bact: B+PB
Euk: E+PE
B_introns: average number of introns in Bacterial genes

E_introns: average number of introns in Eukaryotic genes
Euk_F: eukaryotic genes taxonomically classified as F
Euk_M: eukaryotic genes taxonomically classified as M
Euk_O: eukaryotic genes taxonomically classified as O
Euk_E: eukaryotic genes taxonomically classified as E
Euk_D: eukaryotic genes taxonomically classified as D

Decision-tree for *P. chileana*, third round of decontamination:

```
If qcPCAU or If qcNCBI >= 50%: --> N

Else:
  If pY: --> by default Y, but:
        If cov <= 25:
              If (Bact + Euk) > 1:
                    If Bact > Euk:
                          If Euk = 0: --> N
                          If Euk > 1:
                                If B_introns > 0: --> Y
                                Else: --> D
                    or If:
                          Euk_E > (Euk_F+Euk_M+Euk_O+Euk_D):
              Else: --> D
        If cov > 25:
              If (Bact + Euk) > 1:
                    If Euk < Bact:
                          If Euk = 0:
                                If B_introns > 0:
                                      If Euk_F < Euk_E: --> N
                                      Else: --> D
                                Else: --> N
                          Else:
                                If B_introns > 0: --> Y
                                Else: --> D
                                If Euk_F < Euk_E: --> D

  If pN: --> by default N, but:
        If cov > 25:
              If Euk > Bact:
                    If E_introns > 0:
                          If Euk_E < (Euk_F+Euk_M+Euk_O+Euk_D):
                                If Bact > 0:
                                      If B_introns > 0: --> D
                                Else: --> D
```

```
If pD: --> by default N, but:
    If cov <= 25:
        If Euk > 0:
            If (Euk_M+Euk_F+Euk_O+Euk_D) >= Euk_E: --> D
            If Euk_F > 0: --> D
        If Euk + Bact > 0:
            If Euk < Bact:
                If B_introns > 0: --> D
                Else: --> N
            If Euk = 0: --> N
    If cov > 25: --> by default D, but:
        If Euk + Bact > 0:
            If Euk < Bact: --> by default N, but:
                If Euk_F > 0: --> D
                If B_introns > 0:
                    If Euk > 0: --> D
                    Else:
                        If B_introns > 2: --> D
            or If Euk > 0:
                If (Euk_M+Euk_F+Euk_O) > (Euk_D+Euk_E): --> D
                Else: --> Y

If less2000: --> by default N, but:
    If cov <= 25:
        If Euk > 0:
            If Euk_F > (Euk_M+Euk_O+Euk_D+Euk_E): --> D
    If cov > 25:
        If Euk > 0:
            If Euk < Bact:
                If (Euk_F+Euk_M+Euk_O) > (Euk_D+Euk_E):
                    If B_introns > 0: --> Y
                    Else: --> D
            or If Bact = 0: --> by default D, but:
                If (Euk_M+Euk_F+Euk_O) > (Euk_D+Euk_E): --> Y
                Else: --> D
            Else:
                If (Euk_F+Euk_M+Euk_O) > (Euk_D+Euk_E):
                    If B_introns > 0: --> Y
                    Else --> D
```

Scaffolds classified as ‘*P. chileana*’ (4119 scaffolds, 43905177 bp) and as ‘Uncertain’ (774 scaffolds, 3157320 bp) by the decision-tree were included in the *Pchi.gDNA.clean.v1.fasta*, with

the suffix '_potentialcontaminant' added to 'Uncertain' scaffolds. 18S and mitochondrial scaffolds (2 putative mitochondrial scaffolds, 38854 bp) were labeled as 'ribosomal' and '_mitochondrial', respectively.

4) Genome annotation and masking of repetitive regions

The genomes were annotated using *BRAKER1*, as we did for all preliminary annotations performed during the decontamination steps (explained in *M. vibrans* section). However, in this case, we first estimated the maximum intron length of every species for the --max-segment-intron and --max-intron-length *TopHat* parameters. For that, we aligned the RNA-seq *de novo* assembled transcripts of each species with its genome [*BLASTn*], and inferred potential intron positions and lengths from the discontinuities in the alignments between every query transcript and its best targeting genomic scaffold (only alignments of transcripts showing > 95% of average query coverage and identity with its best targeting scaffold were considered). The values for both *TopHat* parameters were set to 17500, 7500, 2500 and 6000 for *M. vibrans, P. atlantis, P. vietnamica,* and *P. chileana*, respectively.

We used *PASA*[29] *v2.0.2* to refine the *BRAKER1* annotations. *PASA* was ran using as input the transcripts from both *de novo* and genome-guided assemblies, but only those that aligned with its best targeting genomic scaffold with >90% of average query coverage and identity [*BLASTn*]. *De novo* transcriptome assemblies were done with *Trinity*, as explained in *M. vibrans* section. Genome-guided transcriptome assemblies were also done with *Trinity* [--normalize_reads, --jaccard_clip], using the same *accepted_hits.bam* file as the used for *BRAKER1* annotations, and also limiting the maximum intron lengths as with *TopHat* alignments. *PASA* annotations were obtained using both *blat*[30] *v35x1* and *GMAP*[31] *v2015-12-31* aligners, with the suggested --stringent_alignment_overlap and --gene_overlap parameters, and after having been preprocessed the transcripts from adapter/vector sequences using *seqclean* (https://sourceforge.net/projects/seqclean/). PASA was run again to add UTR information and correct some *BRAKER1* annotations by means of two rounds of annotation comparison (as recommended by the manual). Protein sequences were finally retrieved from corrected *BRAKER1* annotations, keeping only the longest isoform per gene. Protein sequences corresponding to genes predicted on scaffolds labeled as "_potentialcontaminant" were equally labeled.

We masked the repetitive regions of the genomes with Ns using *RepeatMasker*[32] *version open-4.0.6* (e.g. *Mvib.gDNA.v1.fasta.masked*). For that, we first created for every genome a specific library of repetitive sequences using *RepeatModeler v1.0.4* (www.repeatmasker.org).

5) Correction of false gene fusion/fission events

Gene fusion and domain rearrangement are important sources of protein innovation in eukaryotic evolution[33]. These events lead to the emergence of composite gene families, which emerge from the merging of distinct component families[34]. In a composite gene, the regions corresponding to the distinct components show similarities at sequence level to distinct sets of proteins (component families), a pattern that can be detected using sequence-similarity tools (e.g., *BLAST*)[35]. While the finding of composite genes may well correspond to true fusion events, they can also occur by errors during the annotation process (e.g., the software may confuse an intergenic region with an intron). A preliminary analysis of the *M. vibrans* annotations revealed a *bona fide* example of false composite (Mvib_g619), a protein of 891 aa with an unexpected Pfam domain architecture (i.e., not found in other eukaryotes) that was not supported by the transcriptomic data.

We evaluated the assembled genomes to detect and split those genes showing strong evidences of being false composites. To do that, we developed a Python script to detect and split the components (i.e., the true separate genes) within the false composites by analysing results from *BLAST* alignments of the predicted proteins with two databases: (1) a nucleotide database with the RNA-seq *de novo* assembled transcripts of the corresponding species; and (2) a taxonomically-rich database of eukaryotic proteins (euk_db). *BLAST* alignments were performed separately with each database, using *tBLASTn* and *BLASTP* for (1) and (2), respectively [-evalue 1e-3]. In an alignment of a novel composite (either true or false), we expect to observe distinct regions of the composite sequence to differentially align with distinct protein sets from euk_db, each set corresponding to a component family[35]. However, to be considered as a *bona fide* composite, the existence of the entire coding sequence must be supported by the transcriptomic data. In other words, a composite is likely to be false if the discontinuities observed in the alignments with euk_db proteins are also observed in the alignments with the transcripts.

The criteria to detect false composites and split them into different components (true genes) consisted in finding those positions (split positions) where all the targets (RNA-seq transcripts and eukaryotic proteins) that aligned with any upstream position did not align neither with the current position nor with any downstream position. However, we adapted this criteria to overcome the limitations of inferring the exact positions that delimit the homologous regions between proteins from *BLAST* alignments[36]. In particular, we found cases where a manual inspection of the alignments strongly suggested the existence of different components, with different regions of the false composite aligning with differentiated sets of targets. However, a little overlap occurred between the ending and starting positions of the alignments corresponding to the different components, most likely because of imprecisions in the alignments. Hence, an algorithm screening for absolute discontinuities between each pair of consecutive positions will fail to detect a split position when these overlaps occur. Instead, to skip these misleading contiguity regions, we evaluated discontinuities between all pairs of positions separated by distances of 15 aa. Thus, a split position is inferred when the targets that aligned from the 0 to n positions do not align with

any position from n+15 to the end of the sequence; with two exceptions: (1) we discarded split positions that would lead to the inference of a component shorter than 30 aa; and (2) when the region delimited by the fifteen upstream and downstream positions to the split position is within the same predicted Pfam[37] domain [*PfamScan*].

Using this script, the false composite Mvib_g619 was separated into two components (true genes): Mvib_g8200_1-297 and Mvib_g8200_298-891. This division solved the unexpected and not supported domain architecture shown by Mvib_g619, with the region corresponding to the two 'Uricase' domains and the regions corresponding to the 'ArfGap' domain being now separate in two distinct genes. Overall, a total of 39, 19, 8 and 2 unexpected domain architectures not supported by the RNA-seq data were corrected from the genomes of *M. vibrans*, *P. atlantis*, *P. vietnamica* and *P. chileana*; respectively, this representing the 5.63%, 5.48%, 1.50% and 0.45% of all the unexpected domain architectures found, respectively. The remaining unexpected domain architectures that were not corrected may correspond either to true or to non-corrected false composites. The usage of more stringent alignment conditions would have increased the discontinuities found within transcripts, and hence the number of composites that would have been split. However, because we used a standard genome-annotation pipeline, we expected an overall good performance for this automatic annotation approach (a consideration that is supported by the results from *BUSCO* analyses on protein predictions, see Supplementary Information 1-Fig. 23). Thus, we decided to use a conservative approach and correct only those strong candidates of being false composites (the only constraint in the alignments was an E-value threshold of 1e-3).

In total, we detected 881, 284, 102, and 120 false composites for *M. vibrans*, *P. atlantis*, *P. vietnamica,* and *P. chileana*; respectively, this representing the 7.27%, 3.15%, 0.69%, and 0.83% of all the genes predicted, respectively. The false composite ratios found for *M. vibrans* and *P. atlantis* are clearly higher than those found for both *Pigoraptor* species. These differences may be explained by variances in the contiguity of the assemblies, with the genomes of *M. vibrans* and *P. atlantis*, especially the former, showing better N50 and L75 metrics than the two *Pigoraptor* genomes (Supplementary Information 1-Fig. 23). The reason is that because only neighbor genes can be mispredicted as a false composite, the number of potential false composites predictions should increase proportionally to the contiguity of the genome.

We evaluated the performance of our methodology by counting the differences in the number of reciprocal best hits (RBH) retrieved between *C. owczarzaki* (Filasterea) and each of the four species, when using the pre- and the post-corrected protein annotations, based on the following argument. If the protein A from *C. owczarzaki* (Ca) share orthology with the protein A from *M. vibrans* (Ma), we may expect Ma likely to be the best hit of Ca when aligning all *C. owczarzaki* proteins with *M. vibrans* proteins; and Ca of Ma when aligning all *M. vibrans* proteins with *C.*

owczarzaki proteins. If we erroneously split the protein Ma into two proteins (Ma1 and Ma2), the protein Ca will still share a RBH relationship with a *M. vibrans* protein, either with Ma1 or Ma2. However, if we only consider those alignments covering a high percentage of the total query length (e.g. 75%), it is possible that we did not find any hit between Ca and Ma1 or Ma2 because both *M. vibrans* proteins could be shorter than Ca. Hence, an erroneous split of a gene may imply a decrease in the number of RBH recovered. We thus expect the finding and the correction of false composites to potentially increase the number of RBH recovered. For example, if the proteins A and B from *M. vibrans* (Ma and Mb) are respective orthologs of the proteins A and B from *C. owczarzaki* (Ca and Cb), but Ma and Mb were mispredicted as a false composite (Mab), we would only recover one RBH relation (Ca or Cb to Mab). The correction and split of Mab into Ma and Mb would increase by one the number of RBH relations (Ca to Ma and Cb to Mb).

We aligned all *C. owczarzarki* proteins with the pre- and post-corrected protein annotations of the four species and vice versa, and we counted only those RBH relations in which the corresponding alignments cover at least the 75% of both aligned sequences, with a minimal average identity of 25%. With the post-corrected annotations, we always recovered more RBH relations between *C. owczarzaki* and our target species than with the pre-corrected annotations (19, 9, 2 and 7 for *M. vibrans*, *P. atlantis*, *P. vietnamita* and *P. chileana*; respectively). Moreover, all the *C. owczarzaki* proteins involved in a RBH relation with the pre-corrected proteins were also involved in a RBH relation with the post-corrected proteins. These results altogether suggest that our approach modestly improved the quality of our annotations by detecting and splitting at least some of the mispredicted composites, with no evidence of false-positive splits.

Beyond false composites, a miss-prediction in the annotation process can also lead to erroneous splits of true genes into separate genes (false gene fissions). We also screened the predicted proteins to find and correct clear cases of false fissions in our annotations. We used *Transdecoder* (https://github.com/TransDecoder) to translate the RNA-seq *de novo* assembled transcripts into proteins (transcript proteins), and we kept only those transcript proteins that aligned with at least 95% of query coverage and average identity with the same genomic scaffold (*bona fide* transcript proteins for our organism of interest; to discard potential biases introduced by contaminant transcripts). *Bona fide* transcript proteins were then aligned with the predicted proteins from the genomes [*BLASTp*: -evalue 1e-3]; and we screened for transcript proteins with at least two consecutive regions of its sequence having the best scoring hit with neighbor genes in the genome. We consider the finding of this alignment pattern as strong evidence of false fission, given that polycistronic transcripts are rare in eukaryotes[38] (the few occurrences of this alignment pattern corroborate that this assumption is valid for our genomes, see below). Protein sequences from the false fissioned neighbor genes were joined and aligned with *bona fide* transcript proteins and with euk_db [*BLASTp*: -evalue 1e-3]. After manual inspection of the alignments, we corrected

2 clear false fissions for *P. chileana* and 1 for *M. vibrans*, *P. atlantis* and *P. vietnamita*. All FASTA files produced will be available online as soon as we publish the corresponding manuscript.

6) Assessment of genome quality

We used *QUAST v4.2* [25] to quantify L75 and N50 metrics for all the genomes of the species represented in Supplementary Information 1-Fig. 23A. We also ran *BUSCO v1.22* [24] (*all Eukaryota* dataset) on the genomes and proteomes of these species; and in the case of *M. vibrans*, *P. atlantis*, *P. vietnamica* and *P. chileana*, also on proteomes predicted from the *de novo* assembled previously available transcriptomes[28,39] using *TransDecoder.LongOrfs v3.0.1* (https://github.com/TransDecoder). 'BUSCO C (%)' metric corresponds to the sum of '*Complete BUSCOs*', '*Complete and single-copy BUSCOs*', '*Complete and duplicated BUSCOs*' and '*Fragmented BUSCOs*' metrics (in percentage); whereas 'BUSCO D (%)' and 'BUSCO F (%)' metrics correspond to the percentages of '*Missing BUSCOs*' and '*Complete and duplicated BUSCOs*', respectively (Supplementary Information 1-Fig. 23).

A

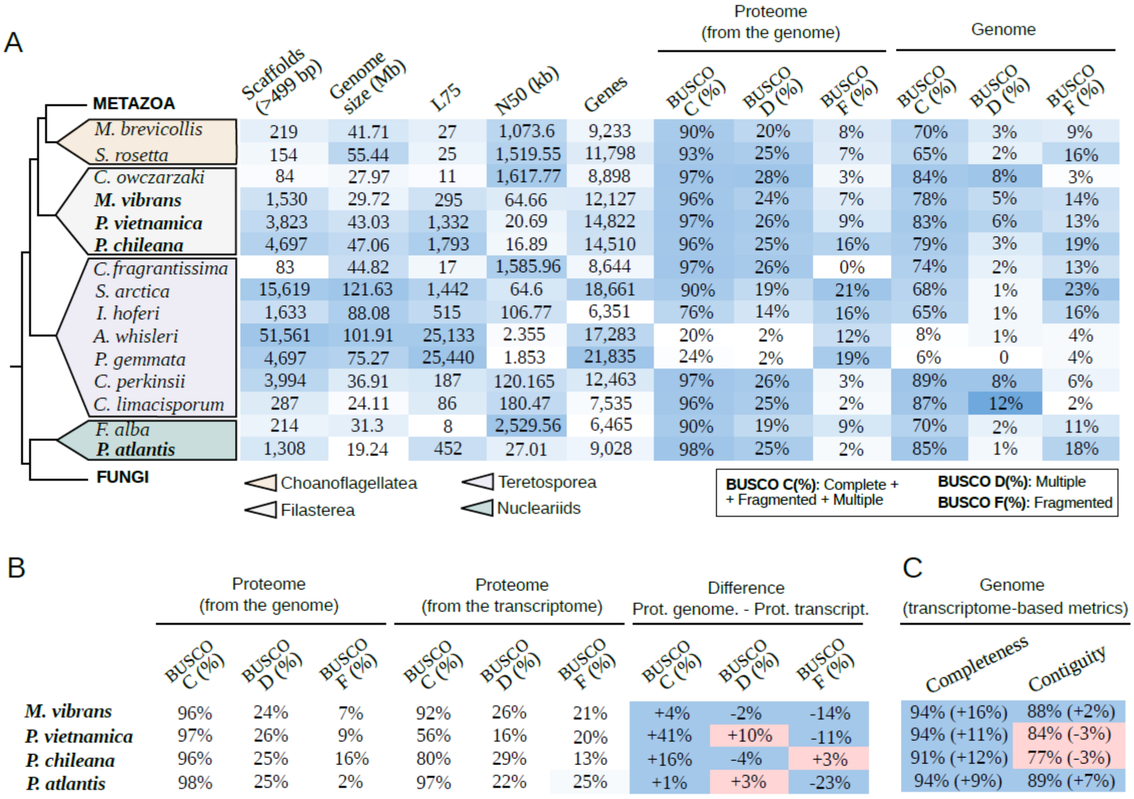

Species	Scaffolds (>499 bp)	Genome size (Mb)	L75	N50 (kb)	Genes	Proteome (from the genome) BUSCO C (%)	BUSCO D (%)	BUSCO F (%)	Genome BUSCO C (%)	BUSCO D (%)	BUSCO F (%)
M. brevicollis	219	41.71	27	1,073.6	9,233	90%	20%	8%	70%	3%	9%
S. rosetta	154	55.44	25	1,519.55	11,798	93%	25%	7%	65%	2%	16%
C. owczarzaki	84	27.97	11	1,617.77	8,898	97%	28%	3%	84%	8%	3%
M. vibrans	1,530	29.72	295	64.66	12,127	96%	24%	7%	78%	5%	14%
P. vietnamica	3,823	43.03	1,332	20.69	14,822	97%	26%	9%	83%	6%	13%
P. chileana	4,697	47.06	1,793	16.89	14,510	96%	25%	16%	79%	3%	19%
C. fragrantissima	83	44.82	17	1,585.96	8,644	97%	26%	0%	74%	2%	13%
S. arctica	15,619	121.63	1,442	64.6	18,661	90%	19%	21%	68%	1%	23%
I. hoferi	1,633	88.08	515	106.77	6,351	76%	14%	16%	65%	1%	16%
A. whisleri	51,561	101.91	25,133	2.355	17,283	20%	2%	12%	8%	1%	4%
P. gemmata	4,697	75.27	25,440	1.853	21,835	24%	2%	19%	6%	0	4%
C. perkinsii	3,994	36.91	187	120.165	12,463	97%	26%	3%	89%	8%	6%
C. limacisporum	287	24.11	86	180.47	7,535	96%	25%	2%	87%	12%	2%
F. alba	214	31.3	8	2,529.56	6,465	90%	19%	9%	70%	2%	11%
P. atlantis	1,308	19.24	452	27.01	9,028	98%	25%	2%	85%	1%	18%

BUSCO C(%): Complete + + Fragmented + Multiple **BUSCO D(%)**: Multiple **BUSCO F(%)**: Fragmented

B

	Proteome (from the genome) BUSCO C (%)	BUSCO D (%)	BUSCO F (%)	Proteome (from the transcriptome) BUSCO C (%)	BUSCO D (%)	BUSCO F (%)	Difference Prot. genome. - Prot. transcript. BUSCO C (%)	BUSCO D (%)	BUSCO F (%)
M. vibrans	96%	24%	7%	92%	26%	21%	+4%	-2%	-14%
P. vietnamica	97%	26%	9%	56%	16%	20%	+41%	+10%	-11%
P. chileana	96%	25%	16%	80%	29%	13%	+16%	-4%	+3%
P. atlantis	98%	25%	2%	97%	22%	25%	+1%	+3%	-23%

C

Genome (transcriptome-based metrics)	Completeness	Contiguity
M. vibrans	94% (+16%)	88% (+2%)
P. vietnamica	94% (+11%)	84% (-3%)
P. chileana	91% (+12%)	77% (-3%)
P. atlantis	94% (+9%)	89% (+7%)

Supplementary Information 1-Fig. 23. Completeness and contiguity metrics of the genomes from the four species sequenced (highlighted in bold font) and also from other unicellular relatives of Metazoa and Fungi with publicly available genome data. BUSCO software metrics are expressed in % (429 BUSCO markers), and were computed for the genomic protein predictions and for the genomic scaffolds, and (**B**) also for the protein predictions obtained from the previously available transcriptomic data for *M. vibrans*[39], *P. vietnamica*[28], *P. chileana*[28] and *P. atlantis*[39] using Transdecoder software (https://github.com/TransDecoder). The white/blue gradients in (**A**) are column-specific and represent differences between metric values (from the lowest to the highest). The blue/red code in (**B**) illustrates when

the metrics are better or worse for the protein predictions coming from the genome than for those predictions coming from the transcriptome, respectively. (**C**) Genome completeness and contiguity metrics estimated from alignments of *bona fide* transcripts of each species to the corresponding genome. In particular, we estimated completeness by counting how many *bona fide* transcripts of the species aligned with the genome with an average identity of >95% and with a total query coverage of >95%. Contiguity was estimated as completeness, but only those hits involving the genomic fragment to which each transcript aligned with the highest score were considered (e.g., a transcript which sequence is complete but split into distinct genomic fragments will sum for completeness but not for contiguity). Differences between this transcriptome-based metrics and those found by BUSCO are indicated within the parenthesis, with the square being colored in blue or red according to whether the transcriptome-based metrics indicated a greater or a worse quality for the genome than the BUSCO metrics. Note that transcriptome-based completeness and contiguity metrics should be compared to 'BUSCO C(%)' and [100 - 'BUSCO F(%)'], respectively. Number of *bona fide* transcript sequences (i.e., markers) per species: *M. vibrans* 10,056; *P. vietnamica*: 746; *P. chileana*: 2,200; *P. atlantis*: 5,841

An alternative transcriptome-based approach to assess completeness and contiguity (Supplementary Information 1-Fig. 23C) was applied to *M. vibrans*, *P. atlantis*, *P. vietnamica* and *P. chileana* genomes. It consists of aligning [*BLASTn* [8]: -evalue 1e-3] a set of *bona fide* transcripts from these species to the corresponding genome, and computing the completeness and contiguity according to alignment results. In particular, we estimated completeness by counting how many transcripts aligned with the genome with an average identity of >95% and with a total query coverage of >95%. Contiguity was estimated as completeness, but only hits with the best scoring target genomic fragment were considered (e.g., a transcript in which the sequence is complete but split into distinct genomic fragments will sum for completeness but not for contiguity). For *M. vibrans* and *P. atlantis*, we used the set of *bona fide* transcripts defined during the decontamination process (see Supplementary Material 1). For both *Pigoraptor* species, the set was constructed in the following manner: we first performed a *de novo* transcriptome assembly of RNA-seq reads of these species using *Trinity v2.2.0* [40] [--jaccard_clip, --normalize_reads], previously preprocessed using *trimmomatic v0.36* [4] [TruSeq3-PE-2.fa:2:30:10, SLIDINGWINDOW:4:5, LEADING:5, TRAILING:5, MINLEN:25]. The raw RNA-seq reads previously used to produce the transcriptomic data used in [28] were kindly provided by Elisabeth Hehenberger. We then used *TransDecoder.LongOrfs* to keep only transcripts corresponding to complete coding sequences, which were aligned to the metagenomes of *Parabodo caudatus* and of the corresponding *Pigoraptor* species [*BLASTn*: -evalue 1e-3]. Transcripts that aligned to *Pigoraptor* but not to the *P. caudatus* metagenome were kept and aligned to euk_prok_db [*BLASTx*: -task blastx-fast, -evalue 1e-3] (see Supplementary Material 1), and only those whose best scoring hit was a protein from *Capsaspora owczarzaki* (the only filasterean in the dataset) were considered as the *bona fide* transcripts, which were lastly processed for redundancy removal using *CD-HIT v4.6* [18].

References

1. Tong, S. M. Heterotrophic flagellates and other protists from Southampton Water, U.K. *Ophelia* **47**, 71–131 (1997).
2. Marron, A. O., Akam, M. & Walker, G. A Duplex PCR-Based Assay for Measuring the Amount of Bacterial Contamination in a Nucleic Acid Extract from a Culture of Free-Living Protists. *PLoS One* **8**, e61732 (2013).
3. Torruella, G., de Mendoza, A., Grau-Bové, X. & Ruiz-Trillo, I. Phylogenomics Reveals Convergent Evolution of Lifestyles in Close Relatives of Animals and Fungi. *Curr. Biol.* **25**, 2404–2410 (2015).
4. Bolger, A. M., Lohse, M. & Usadel, B. Trimmomatic: a flexible trimmer for Illumina sequence data. *Bioinformatics* **30**, 2114–2120 (2014).
5. O'Connell, J. *et al.* NxTrim: Optimized trimming of Illumina mate pair reads. *Bioinformatics* **31**, 2035–2037 (2015).
6. Bankevich, A. *et al.* SPAdes: A New Genome Assembly Algorithm and Its Applications to Single-Cell Sequencing. *J. Comput. Biol.* **19**, 455–477 (2012).
7. Grau-Bové, X. *et al.* Dynamics of genomic innovation in the unicellular ancestry of animals. *Elife* **6**, e26036 (2017).
8. Altschul, S. F., Gish, W., Miller, W., Myers, E. W. & Lipman, D. J. Basic local alignment search tool. *J. Mol. Biol.* **215**, 403–410 (1990).
9. Burger, G., Gray, M. W., Forget, L. & Lang, B. F. Strikingly Bacteria-Like and Gene-Rich Mitochondrial Genomes throughout Jakobid Protists. *Genome Biol. Evol.* **5**, 418–438 (2013).
10. Hoff, K. J., Lange, S., Lomsadze, A., Borodovsky, M. & Stanke, M. BRAKER1: Unsupervised RNA-Seq-based genome annotation with GeneMark-ET and AUGUSTUS. *Bioinformatics* **32**, 767–769 (2015).
11. Besemer, J. & Borodovsky, M. GeneMark: web software for gene finding in prokaryotes, eukaryotes and viruses. *Nucleic Acids Res.* **33**, W451–W454 (2005).
12. Stanke, M. *et al.* AUGUSTUS: ab initio prediction of alternative transcripts. *Nucleic Acids Res.* **34**, W435-439 (2006).
13. Le, H. S., Schulz, M. H., Mccauley, B. M., Hinman, V. F. & Bar-Joseph, Z. Probabilistic error correction for RNA sequencing. *Nucleic Acids Res.* **41**, e109 (2013).
14. Kim, D. *et al.* TopHat2: accurate alignment of transcriptomes in the presence of insertions, deletions and gene fusions. *Genome Biol.* **14**, R36 (2013).
15. Adl, S. M. *et al.* The Revised Classification of Eukaryotes. *J. Eukaryot. Microbiol.* **59**, 429–514 (2012).
16. Dick, G. J. *et al.* Community-wide analysis of microbial genome sequence signatures. *Genome Biol.* **10**, R85 (2009).
17. Langmead, B. & Salzberg, S. L. Fast gapped-read alignment with Bowtie 2. *Nat. Methods* **9**, 357–359 (2012).
18. Fu, L., Niu, B., Zhu, Z., Wu, S. & Li, W. CD-HIT: accelerated for clustering the next-generation sequencing data. *Bioinformatics* **28**, 3150–3152 (2012).
19. Alneberg, J. *et al.* Binning metagenomic contigs by coverage and composition. *Nat. Methods* **11**, 1144–1146 (2014).
20. Zaremba-Niedzwiedzka, K. *et al.* Asgard archaea illuminate the origin of eukaryotic cellular complexity. *Nature* **541**, 353–358 (2017).

21. López-Escardó, D., López-García, P., Moreira, D., Ruiz-Trillo, I. & Torruella, G. Parvularia atlantis gen. et sp. nov., a Nucleariid Filose Amoeba (Holomycota, Opisthokonta). *J. Eukaryot. Microbiol.* **65**, 170–179 (2018).
22. Peng, Y., Leung, H. C. M., Yiu, S. M. & Chin, F. Y. L. IDBA-UD: A de novo assembler for single-cell and metagenomic sequencing data with highly uneven depth. *Bioinformatics* **28**, 1420–1428 (2012).
23. Boisvert, S., Laviolette, F. & Corbeil, J. Ray: Simultaneous Assembly of Reads from a Mix of High-Throughput Sequencing Technologies. *J. Comput. Biol.* **17**, 1519 (2010).
24. Simao, F. A., Waterhouse, R. M., Ioannidis, P., Kriventseva, E. V. & Zdobnov, E. M. BUSCO: Assessing genome assembly and annotation completeness with single-copy orthologs. *Bioinformatics* **31**, 3210–3212 (2015).
25. Gurevich, A., Saveliev, V., Vyahhi, N. & Tesler, G. QUAST: Quality assessment tool for genome assemblies. *Bioinformatics* **29**, 1072–1075 (2013).
26. Vollmers, J., Wiegand, S. & Kaster, A. K. *Comparing and evaluating metagenome assembly tools from a microbiologist's perspective - Not only size matters! PLoS ONE* **12**, (2017).
27. Li, H. *et al.* The Sequence Alignment/Map format and SAMtools. *Bioinformatics* **25**, 2078–9 (2009).
28. Hehenberger, E. *et al.* Novel Predators Reshape Holozoan Phylogeny and Reveal the Presence of a Two-Component Signaling System in the Ancestor of Animals. *Curr. Biol.* **27**, 2043–2050 (2017).
29. Haas, B. J. *et al.* Improving the Arabidopsis genome annotation using maximal transcript alignment assemblies. *Nucleic Acids Res.* **31**, 5654–5666 (2003).
30. Kent, W. J. BLAT--the BLAST-like alignment tool. *Genome Res.* **12**, 656–64 (2002).
31. Wu, T. D. & Watanabe, C. K. GMAP: a genomic mapping and alignment program for mRNA and EST sequences. *Bioinformatics* **21**, 1859–1875 (2005).
32. Chen, N. Using RepeatMasker to Identify Repetitive Elements in Genomic Sequences. *Curr. Protoc. Bioinforma.* **5**, 4.10.1-4.10.14 (2004).
33. Basu, M. K., Carmel, L., Rogozin, I. B. & Koonin, E. V. Evolution of protein domain promiscuity in eukaryotes. *Genome Res.* **18**, 449–461 (2008).
34. Haggerty, L. S. *et al.* A pluralistic account of homology: Adapting the models to the data. *Mol. Biol. Evol.* **31**, 501–516 (2014).
35. Pathmanathan, J. S., Lopez, P., Lapointe, F.-J. & Bapteste, E. CompositeSearch: A Generalized Network Approach for Composite Gene Families Detection. *Mol. Biol. Evol.* **35**, 252–255 (2017).
36. Slater, G. & Birney, E. Automated generation of heuristics for biological sequence comparison. *BMC Bioinformatics* **6**, 31 (2005).
37. Finn, R. D. *et al.* Pfam: The protein families database. *Nucleic Acids Res.* **42**, D222–D230 (2014).
38. Blumenthal, T. Gene clusters and polycistronic transcription in eukaryotes. *BioEssays* **20**, 480–487 (1998).
39. Torruella, G. *et al.* Phylogenomics Reveals Convergent Evolution of Lifestyles in Close Relatives of Animals and Fungi. *Curr. Biol.* **25**, 2404–2410 (2015).
40. Haas, B. J. *et al.* De novo transcript sequence reconstruction from RNA-seq using the Trinity platform for reference generation and analysis. *Nat. Protoc.* **8**, 1494–1512 (2013).
