# Supplementary material for: Divergent genomic trajectories predate the origin of animals and fungi
Source: Nature. 2022 Aug 24;609(7928):747–53. doi: 10.1038/s41586-022-05110-4 (PMC9492541; doi:10.1038/s41586-022-05110-4)
Supplement: Supplementary file 4 — Explanation of the MAPBOS pipeline. [file 41586_2022_5110_MOESM4_ESM.pdf]

Supplementary Information 2

1) Exploring distinct sequence similarity-based clustering algorithms

Defining a set of reliable gene families is typically done through algorithms that group sequences into clusters whose members share more similarity between them than to sequences outside the group[1], with clusters being treated as proxies of gene families. What is complicated in this process is to determine the boundaries between clusters, and to determine the appropriate metric to measure similarity. The most currently used software for this task is probably *OrthoFinder*[2], which relies on the MCL clustering algorithm[1] and was designed with the purpose of correcting biases introduced by the heterogeneity in protein length and by the differential phylogenetic distance between taxa. We explored the behavior of three distinct approaches: (1) *OrthoFinder* (*OF*); (2) using the MCL directly as done in [3] (this approach will be hereafter referred to as *MCL*); and (3) *FamilyDetector* (*FD*), which is the clustering algorithm used by default by *CompositeSearch*[4] (the software that we chose to find composite gene families in our dataset).

These three approaches share two common features: on the one hand, they construct a sequence similarity network (SSN) from an all-against-all alignment of a given sequence dataset, in which every sequence is a node in the SSN, and every pair of nodes is connected through an edge if the represented sequences aligned upon a given E-value or any other threshold considered. On the other hand, a clustering algorithm explores the connected components of the SSN (groups of nodes all of which are directly connected between them or indirectly connected through other nodes) and splits them into clusters according to the metrics shown by the edges. In particular, the second approach is similar to the first, as *OF* also relies on MCL for the clustering step, but the difference is that *OF* preprocesses the alignment results by applying a relaxed reciprocal best hit[5] filtering criteria and by normalizing scores according to protein length and phylogenetic distance[2]. The second approach thus consisted in loading the draft alignment results into MCL without being preprocessed, using alignment E-values to weight the edges in the SSN. In contrast, *OF* (the first approach) loads the preprocessed alignment results into MCL and uses normalized alignment scores instead of E-values to weight the SSN edges[2]. Finally, the third approach (*FD*) is substantially different from the former two. *FD* uses a distinct clustering algorithm as well as distinct similarity metrics[4,6]. First, alignment hits lower than a certain identity threshold are discarded. Then, a SSN is constructed from the non-discarded hits, in which edges are weighted according to whether every pair of aligned sequences overcome or not the mutual coverage (mcov) metric. This metric corresponds, for every pair of aligned sequences, to the sum of the lengths covered by the aligned regions of the query and target sequences with respect to the total length of both sequences[4]. Therefore, in *FD*, the output clusters include only nodes whose shared similarity extends to most of their sequence lengths. This contrasts with *OF* and *MCL* approaches, in which the algorithm explores the SSN and splits the connected components into clusters

according to score/E-value metrics, not explicitly taking into account the mutual coverage information[1,2]. We measured how these algorithmic differences affect the behavior of these clustering approaches by evaluating the distribution of sequences by cluster size (Supplementary Information 2-Fig. 1) and the heterogeneity of protein domain architecture within the clusters (Supplementary Information 2-Fig. 2). In particular, we explored two distinct values for the inflation parameter (-I) for *OF* and *MCL* approaches (-I 1.5 and -I 2), and two distinct mcov values for *FD* (mcov 80% and mcov 40%, both with 20% identity threshold).

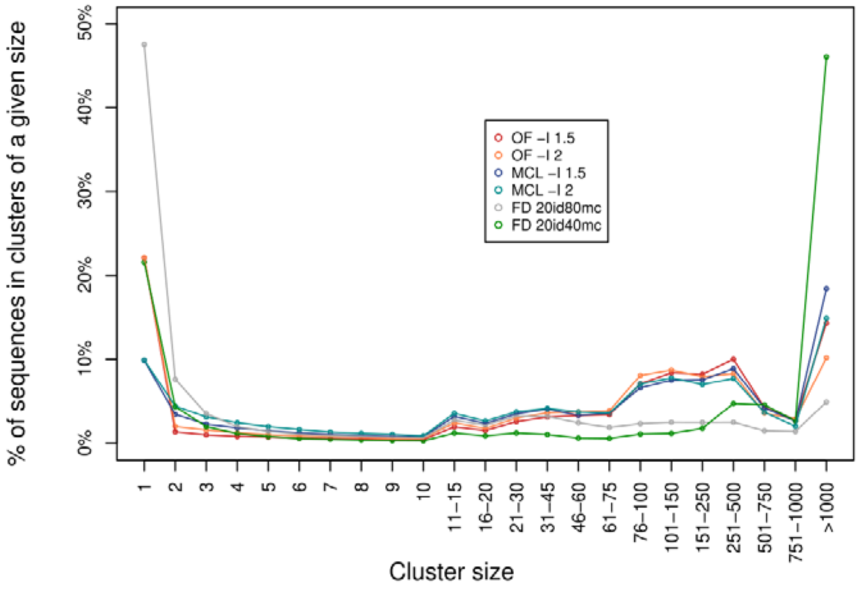

Supplementary Information 2-Fig. 1. Distribution of sequences by cluster size in draft_euk_db according to distinct clustering methods. For example, the ~20% value of 'OF -I2' cluster size 1 indicates that 20% of the sequences within draft_euk_db dataset are in clusters of one single member (i.e., 20% of the sequences are singletons). 'OF -I 1.5': *OrthoFinder* with inflation parameter of 1.5; 'OF -I 2': *OrthoFinder* with inflation parameter of 2; 'MCL -I 1.5': direct *MCL* with inflation parameter of 1.5; 'MCL -I2': direct *MCL* with inflation parameter of 2; 'FD20id80mc': *FamilyDetector*, 20 % percent identity threshold and 80% of mutual coverage; 'FD20id40mc': *FamilyDetector*, 20 % percent identity threshold and 40% of mutual coverage.

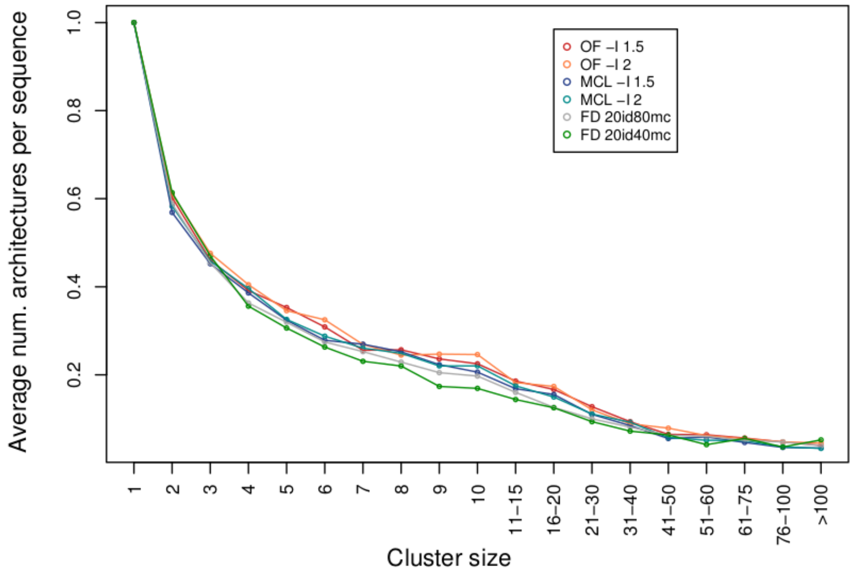

Supplementary Information 2-Fig. 2. Average domain architecture heterogeneity by cluster size in draft_euk_db dataset according to distinct clustering methods. For each cluster, the number of distinct domain architectures was counted and divided by the number of members within the cluster. These values were averaged between all clusters of a given size. For example, the value of 'OF -I 2' 0.40 in cluster size 4 in indicates that, on average, the clusters of 4 members show 0.40 architectures per member (i.e., the clusters of size 4 shown on average 1.6 distinct domain architectures). Only clusters in which all members have a Pfam domain architecture annotated were considered, as for example, a cluster with two members without domain annotations could correspond either to proteins with distinct architectures or to proteins with a same architecture not represented in Pfam database. 'OF -I 1.5': *OrthoFinder* with inflation parameter of 1.5; 'OF -I 2': *OrthoFinder* with inflation parameter of 2; 'MCL -I 1.5': direct *MCL* with inflation parameter of 1.5; 'MCL -I2': direct *MCL* with inflation parameter of 2; 'FD 20id80mc': *FamilyDetector*, 20 % percent identity threshold and 80% of mutual coverage; 'FD 20id40mc': *FamilyDetector*, 20 % percent identity threshold and 40% of mutual coverage.

The distribution of draft_euk_db sequences by cluster size (Supplementary Information 2-Fig. 1) indicates similar tendencies for *OF* and *MCL* approaches, as could be expected given that both rely on the same clustering algorithm. However, some differences are observed between them. In particular, *OF* leads to ~10% more sequences distributed as singletons (i.e., clusters of one single member) than *MCL* and also to more sequences distributed in clusters of 76-500 members (medium clusters), whereas *MCL* shows more sequences in clusters of 2-60 members (small clusters), and ~5% more sequences distributed in large clusters (>1000 members). These differences are probably explained because the SSN in *OF* is more disconnected than in *MCL* due to the reciprocal best hit filter, and hence some sequences in small and large *MCL* clusters could be in singleton and medium clusters in *OF*. A higher inflation value is expected to increase the granularity of the clusters (i.e., to lead to more and smaller clusters) [1]. Accordingly, -I 2 led to

more sequences distributed in 2-150 size clusters, and -I 1.5 to more sequences distributed in larger clusters (250-1000).

FD shows a very different behavior than *OF* and *MCL*, with ~50% sequences distributed in singletons when a mutual coverage (mcov) value of 80% was chosen. A more relaxed mcov (40%) decreased the number of sequences distributed in singletons by ~30% and led to ~50% of sequences distributed in >1000 size clusters. With independence of the mcov value chosen, *FD* presented less sequences in intermediate size clusters than *MCL* and *OF*, either because sequences were more distributed into singletons (80% mcov) or into >1000 size clusters (40% mcov). The distribution of sequences in a wider range of cluster sizes shown by *OF* and *MCL* is probably explained because the clustering step relies on quantitative information (score/E-value), whereas *FD* considers that all sequences that aligned with a mutual coverage upon a given threshold should belong to the same cluster, independently of the degree of similarity shown between them. Because of this, one may expect *OF* and *MCL* to be better in clustering separately sequences belonging to distinct gene families that may still be sharing some degree of similarity because of having arisen by gene duplication from the same ancestral family. On the other hand, we may expect *FD* to be better in removing spurious connections from proteins that aligned only because of having in common a partial fraction of their domain architectures, as they would align with low mutual coverage values. Accordingly, *FD* led to clusters with less heterogeneity at protein architecture level than *MCL* and *OF* (Supplementary Information 2-Fig. 2).

We next benchmarked the sensitivity and the precision of the algorithms in properly classifying the members of a series of gene families that were chosen given our experience on them: NRT2, EUKNR, CS-pNR, NAD(P)H-NIR and Fd-NIR; the nitrate assimilation protein families[7], and ORC2, ORC3, ORC4 and ORC5; the Origin of Recognition Complex subunit families[8]. The *bona fide* members of these families were identified in our dataset from resources generated in previous studies. For each clustering method, we identified the clusters that include more members of the target families. Then, the sensitivity of the method for each family was measured by counting the percentage of *bona fide* family members grouped within the expected cluster. Precision was measured by counting the percentage of *bona fide* family members among all sequences in the cluster that are from species in which the family was detected. Sequences from species from which the presence of the family is uncertain (unsampled in previous studies) were not considered. The average sensitivity and precision of each clustering method is shown in Supplementary Information 2-Table 1.

Supplementary Information 2-Table 1. Average sensitivity and precision shown by distinct clustering methods.

Method	Sensitivity	Precision	(S+P) / 2

OF -I1.5	0.983	0.64	0.8115
OF -I2	0.983	0.772	0.8775
OF -I2.5	0.967	0.775	0.871
OF -I3	0.922	0.75	0.836
MCL -I1.5	0.985	0.649	0.817
MCL -I2	0.983	0.768	0.8755
FD -id30 mcov80	0.464	0.973	0.7185
FD -id20 mcov80	0.654	0.965	0.8095
FD -id10 mcov80	0.661	0.974	0.8175
FD -id20 mcov70	0.684	0.719	0.7015
FD -id20 mcov60	0.736	0.693	0.7145
FD -id20 mcov50	0.814	0.644	0.729
FD -id20 mcov40	0.88	0.24	0.56

‘*OF* -I1.5’: *OrthoFinder* with inflation parameter of 1.5; ‘*MCL* -I 1.5’: direct *MCL* approach with inflation parameter of 1.5; ‘*FD* -id30 mcov80’: *FamilyDetector* ran with 30 % percent identity threshold and 80% of mutual coverage.

Three classes of clustering methods could be considered according to benchmarking results (Supplementary Information 2-Table 1). The first class would be represented by *OF* and *MCL* methods, which performed with high sensitivity (i.e., members belonging to the same families tend to cluster together) but with moderate precision (i.e., the clusters also include members from other families). The second class would be represented by *FD* with 80% of mutual coverage, which performed with high precision but with poor sensitivity, suggesting that 80% is too stringent as mcov threshold. The third class would be represented by *FD* with less than 80% of mutual coverage, which show worse precision and sensitivity than the first class and worse precision than the second class. Overall, *OF* and *MCL* performed substantially better than *FD* approaches when considering both metrics together, suggesting that score/E-value is a better clustering criteria than mutual coverage. In particular, *OF* -I2 and *MCL* -I 2 have the highest values in the combined metric of sensitivity and precision, although *OF* -I2 was minimally better at precision than *MCL* -I 2. Still, *OF* -I 2 was substantially worse at the level of precision than *FD* with 80%

mcov. We went deep into this and explored whether *OF* -I 2 performed with a homogeneous precision or if there were specific problems with some families that could reveal methodological limitations. We found EUKNR and CS-pNR to be clearly more problematic than the other families, with *OF* -I 2 performing with 0.2 and 0.5 of precision on them, respectively. EUKNR corresponds to the family of canonical eukaryotic nitrate reductases, which originated from the fusion of three distinct proteins: sulfite reductase, cytochrome monodomain protein and FAD/NAD reductase[7]. EUKNR is relatively widely distributed across eukaryotes. In contrast, CS-pNR has only been found in *Creolimax fragrantissima* and *Sphaeroforma arctica*, and corresponds to a putative nitrate reductase originated from the fusion of the N-terminal region of EUKNR (which includes the nitrate reducing module) with the N-terminal region of the nitrite reductase NAD(P)H-NIR[7]. Therefore, both EUKNR and CS-pNR are composite gene families, as they originated by the merging of distinct component families[4].

In a sequence similarity network (SSN), the nodes corresponding to members of a composite family (e.g., EUKNR) are directly connected to the nodes corresponding to their component families. Although the nodes from the distinct component families are not necessarily connected between them through a direct edge, they are at least indirectly connected because of being connected to the nodes from the composite family (see Figure 4 of the reference [7]). Thus, the composite family and all their component families will belong to the same connected component, and the clustering algorithm should split them into distinct clusters as they belong to distinct gene families. However, *OF* and *MCL* -I 2 failed in splitting the sulfite oxidases (SUOX) and the FAD/NAD reductases (FAD/NAD-red) from nitrate reductases (EUKNR), as shown in Supplementary Information 2-Fig. 3. Therefore, the same cluster not only included sequences sharing partial homology relationships (e.g., SUOX and the N-terminal region of EUKNR, or FAD/NAD-red and the C-terminal region of EUKNR), but even included sequences that do not share any homologous region (SUOX and FAD/NAD-red). This is problematic at three levels. First, a single cluster includes distinct families that are functionally distinct, which would lead to erroneous inferences of ancestral gene content. Second, non-homologous sequences cannot be in the same phylogenetic tree[9], and our purpose is to generate reliable clusters from which to reconstruct phylogenies for the reconciliation analyses. Third, cases in which the composite family (EUKNR) appear clustered together with their components (SUOX and FAD/NAD-red) would lead *CompositeSearch* software to miss composite origination events, as composite families are detected by inspecting connections in-between clusters. In the other case in which *OF* performed with poor precision, CS-pNR, the behavior of the algorithm was partially different from that of EUKNR. Instead of being clustered together with the two component families from which CS-pNR originated, CS-pNR was only clustered with NAD(P)H-NIR but not with EUKNR (Supplementary Information 2-Fig. 3). This means that CS-pNR, a nitrate reductase, is not clustered with the EUKNR nitrate reductase, from which the catalytic region of CS-pNR originated, but is clustered

with the nitrite reductases, from which the region involved in the electron transfer originated (FAD/NAD domains) [7].

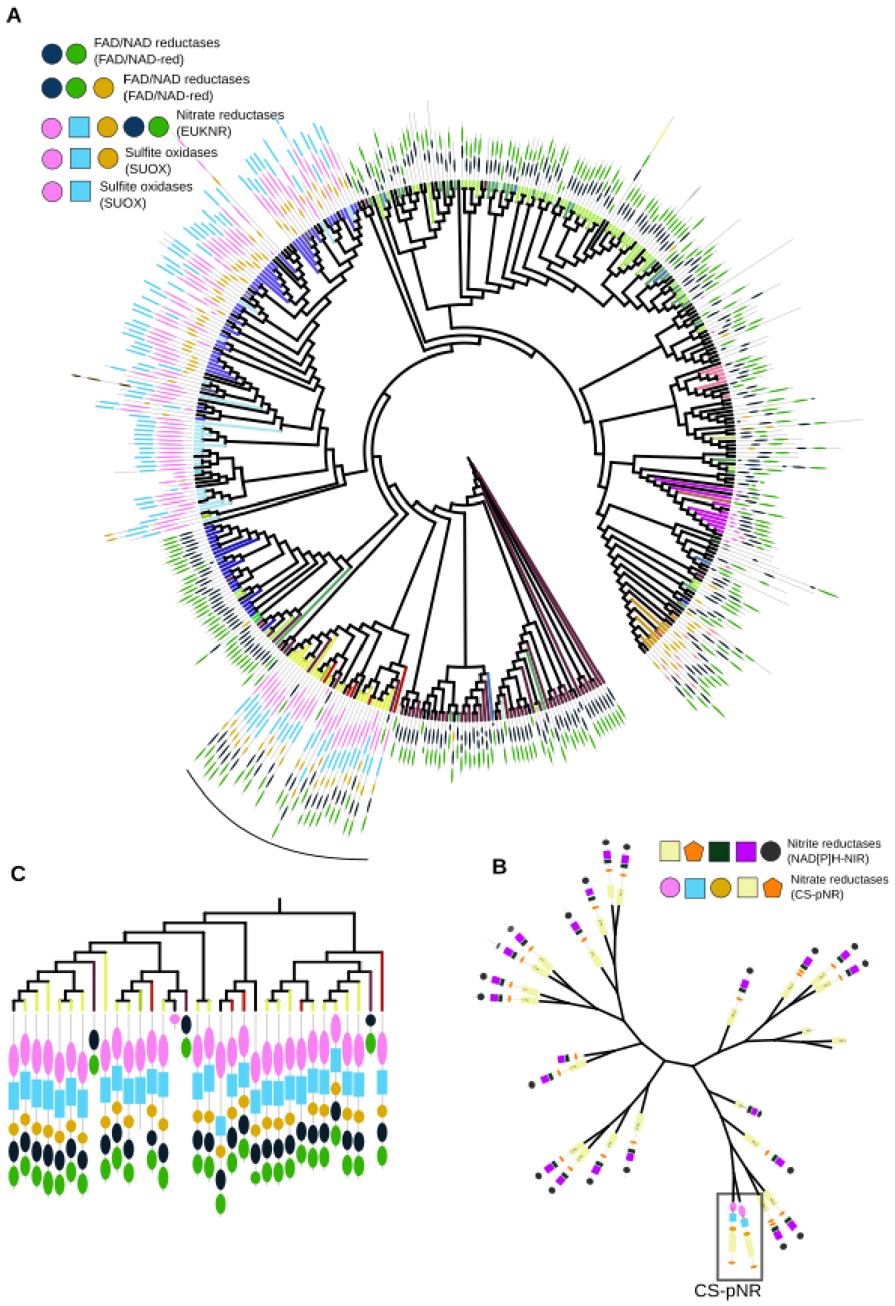

Supplementary Information 2-Fig. 3. (A) Phylogenetic tree of the output cluster from OrthoFinder -I 2 that included the eukaryotic nitrate reductases (EUKNR). This cluster also included component families from which the composite family EUKNR originated: sulfite oxidases (SUOX) and FAD/NAD reductases (FAD/NAD-red). The Pfam protein domains of each sequence is represented. Terminal branch colors correspond to the distinct clusters in which these sequences were split when the clustering algorithm was run on a BLASTp dataset in which hits with <50% of mutual coverage were discarded. (B) A zoom into the region of the tree in (A) that includes the nitrate reductases. (C) Is the same as (A) but for the OrthoFinder -I 2 cluster that included the cadherins. (D) A zoom into a non-specific region of the tree in (B) with the

purpose of showing the heterogeneity at the level of sequence length and protein domain architecture between sequences that branched in a same clade of the tree.

In order to correct the aforementioned undesired *OrthoFinder* behavior, we first explored the possibility of combining the sensitivity shown by *OrthoFinder* (*OF*) with a relaxed mutual coverage (mcov) threshold that could break undesired connections between both composite and component families, since proteins that only share a partial fraction of their domain architecture are expected to align with a lesser mcov than proteins sharing its entire architecture. In particular, 50% appeared as the mcov threshold that leads to the highest average between sensitivity and precision metrics (see Supplementary Information 2-Table 2 in the following section). Despite running *OF* after having discarded *BLASTp* hits below 50% of mcov (*OF* -I2 mcov 50) leads to clusters in which EUKNR is separated from SUOX and FAD/NAD-red (Supplementary Information 2-Fig. 4A), some sequences that appear to branch within the EUKNR clade (Supplementary Information 2-Fig. 4B) clustered with FAD/NAD instead of EUKNR (these could be cases of partial domain losses or misannotated sequences, although errors in the phylogeny shown in the figure are also possible). Most importantly, we found the mcov threshold to be problematic for those families that are rich in repetitive domains, since this lead to differences in length and domain content that do not follow a phylogenetic pattern (see cadherins example in Supplementary Information 2-Fig. 4C-D). The oversplit of a family based on homoplasy reasons (e.g., convergence in sequence length) could lead to missing data and to clusters not defined by

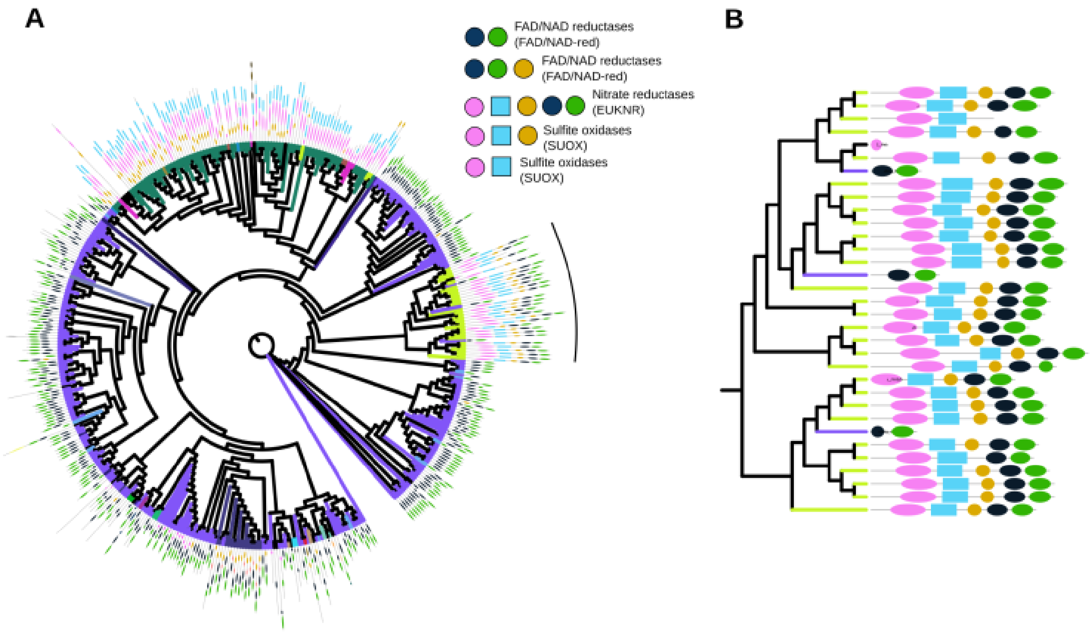

phylogenetic criteria, potentially leading to erroneous inferences in the reconciliation analyses.

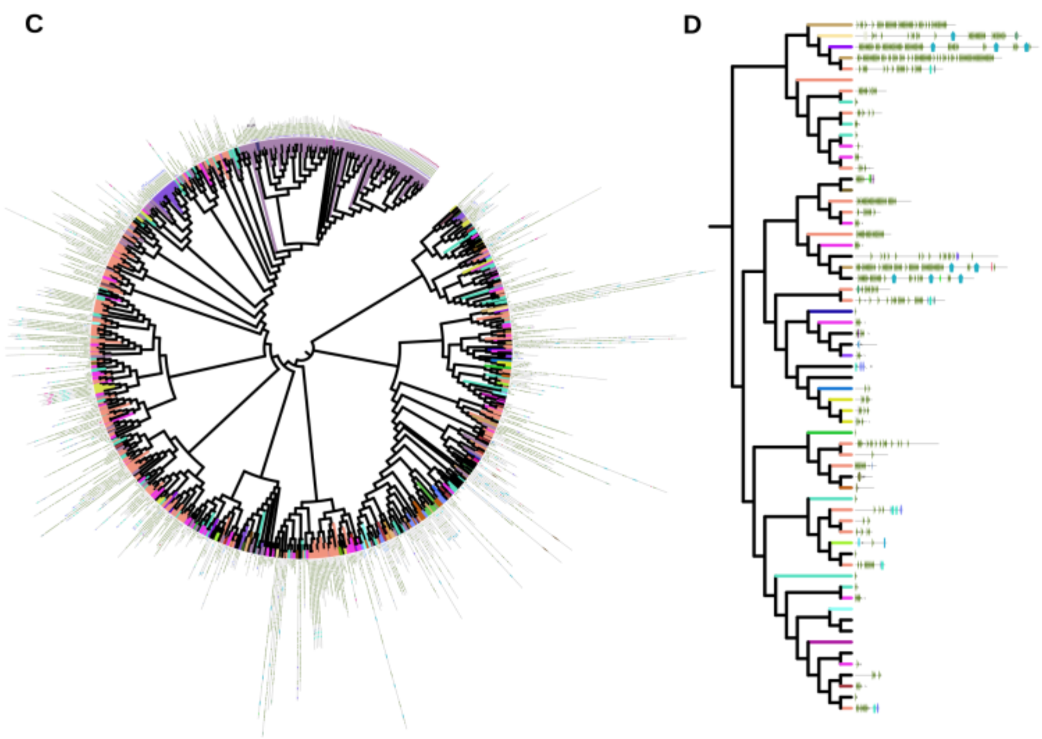

Supplementary Information 2-Fig. 4. (A) Phylogenetic tree of the output cluster from *OrthoFinder* -I 2 that included the eukaryotic nitrate reductases (EUKNR). This cluster also included component families from which the composite family EUKNR originated: sulfite oxidases (SUOX) and FAD/NAD reductases (FAD/NAD-red). The Pfam protein domains of each sequence is represented. Terminal branch colors correspond to the distinct clusters in which these sequences were split when the clustering algorithm was run on a *BLASTp* dataset in which hits with <50% of mutual coverage were discarded. (B) A zoom into the region of the tree in (A) that includes the nitrate reductases. (C) Same as (A) but for the *OrthoFinder* -I 2 cluster that included the cadherins. (D) A zoom into a non-specific region of the tree in (B) with the purpose of showing the heterogeneity at the level of sequence length and protein domain architecture between sequences that branched in a same clade of the tree.

2) Explanation of the *MAPBOS* pipeline

For this study, we consider a definition of gene family in which any reticulation between distinct gene lineages (e.g., domain shuffling or gene fusion) leads to a novel composite gene family, which is expected to show a distinct domain architecture than the component families involved in the reticulation. The objective of the *Multidomain-Aware Pfam-and-Blast-based Orthogroup-Splitter* (*MAPBOS*) pipeline is to fix cases in which *OrthoFinder* mixed into a same orthogroup (OG) sequences belonging to a composite family with sequences belonging to the component families from which the composite family originated (see the example of nitrate reductases explained before). For that, *MAPBOS* splits every OG into sub-orthogroups (sOG), each sOG including the representatives of a domain architecture found in the original OG. This task is accomplished by using distinct split algorithms depending on the characteristics shown by every

OG. *MAPBOS* code is available in the figshare repository (MAPBOS.py, see 'Code availability' statement in the main text).

Schematic representation of the *MAPBOS* algorithm

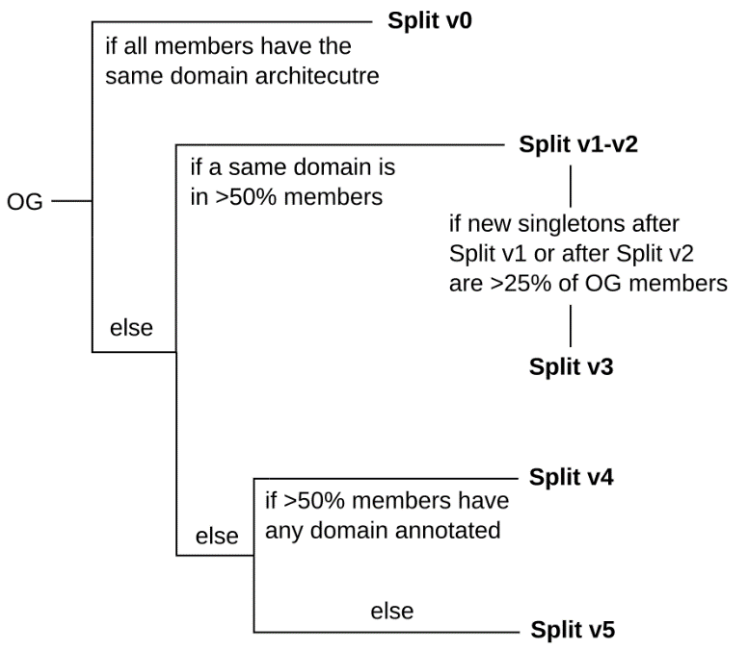

Explanation of the split algorithms

-Split v0: OGs in which the number of distinct architectures is between 0 and 1 are not split.

-Split v1: OG members are grouped according to their Pfam protein domain architectures. Members are classified as MDP (Main Domain architecture Proteins) if they have the most represented Pfam architecture in the OG, and as putative non-MDP if they present an alternative domain architecture according to *PfamScan* results. The algorithm then checks, for every putative non-MDP sequence, if that sequence really has a distinct domain architecture than the MDP (i.e., if the sequence is a *bona fide* non-MDP, or should be instead considered as a MDP). On the one hand, if the only difference in architecture between the non-MDP sequence and MDP is that a domain found in MDP is present in a higher/lower copy number than in the non-MDP, we consider the non-MDP sequence as MDP, as the difference in architecture could be explained because a duplication of an internal domain sequence and not because the acquisition of an additional domain through a reticulation event (e.g., domain shuffling or gene fusion). On the other hand, the algorithm inspects the results from sequence alignments in order to confirm the existence of domains present in the non-MDP sequence and absent in the MDP, and vice versa. For that, every non-MDP (query) is compared to 10 randomly selected MDP (targets). Then, for every domain present in the non-MDP query and putatively absent in MDP (according to *PfamScan* results), the algorithm checks if >75% of the region corresponding to that domain in the non-MPD query has been aligned by at least >3 of the 10 randomly picked MDP targets. If not, the domain

is considered to be absent in MDP, confirming the different domain architecture between the non-MDP query and MDP, and hence the query is considered a *bona fide* non-MDP. If yes, the domain is considered to be present in MDP. Then, the same is done for domains in MDP that are putatively absent in the non-MDP query: the presence of the domain is assumed if >75% of the region corresponding to that domain in >3 of the 10 randomly selected MDP (targets) have been aligned by the non-MDP. Otherwise, the domain is considered to be absent in the non-MDP query, and hence it is considered as *bona fide* non-MDP. Cases of domains whose absence from the non-MDP sequence was discarded according to alignments results could be explained by different reasons. For example, *PfamScan* may not have detected the domain in the non-MDP sequence because of a lack of sensitivity of the corresponding Pfam *Hidden Markov Model*; or because the same region in the non-MDP was annotated as a distinct domain than in MDP (e.g., Rieske_2 and Rieske, respectively). Once each putative non-MDP query have been evaluated, the algorithm excludes from the OG all MDP members, which are clustered apart in an independent sub-OG (sOG), leaving in the original OG all *bona fide* non-MDP query members. The whole process is repeated until each domain architecture represented in the original OG have been split into distinct sOG. See the following illustrations for a graphical representation of Split v1 (Supplementary Information 2-Fig. 5).

Split v1

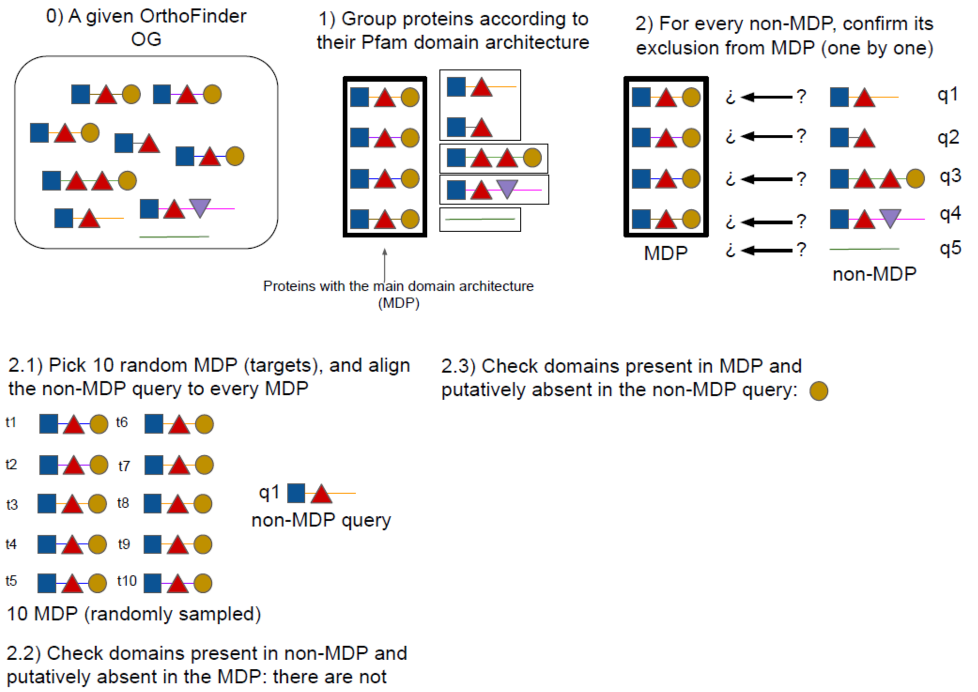

Supplementary Information 2-Fig. 5A

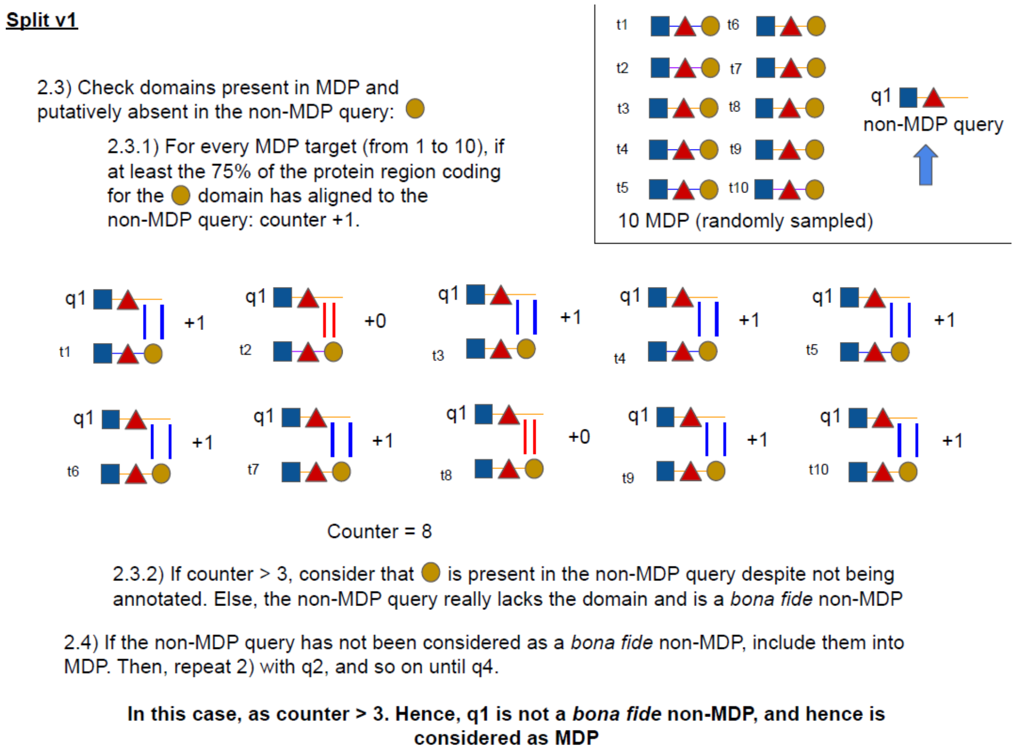

Supplementary Information 2-Fig. 5B

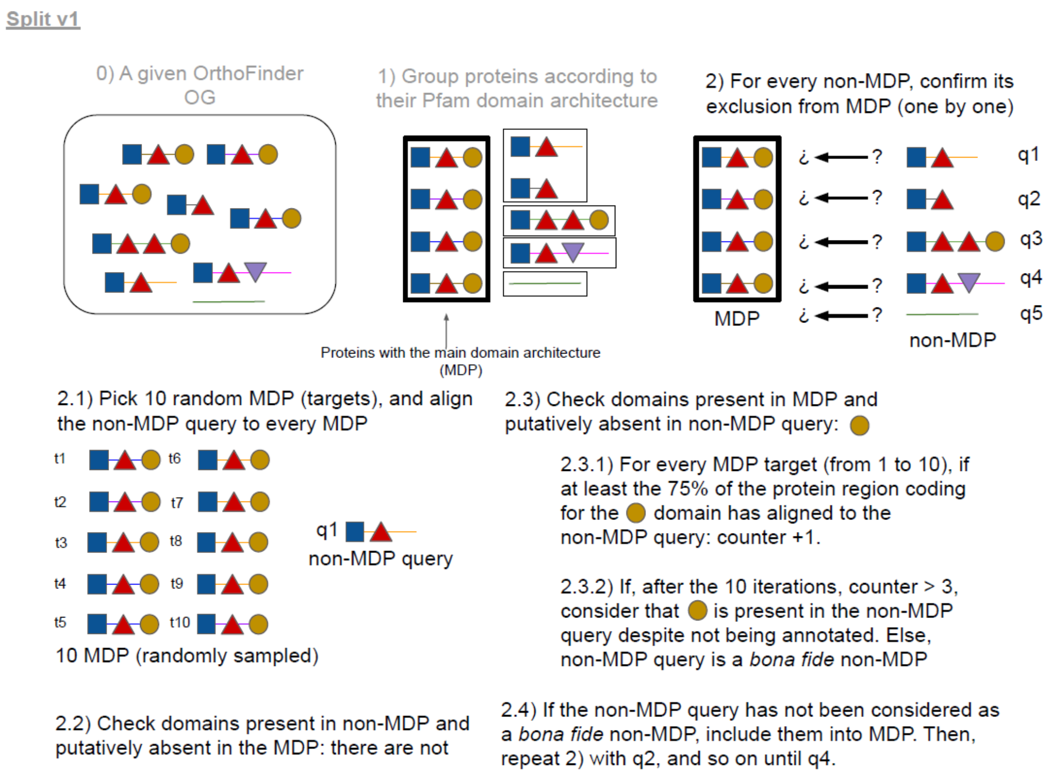

Supplementary Information 2-Fig. 5C

<u>Split v1</u>

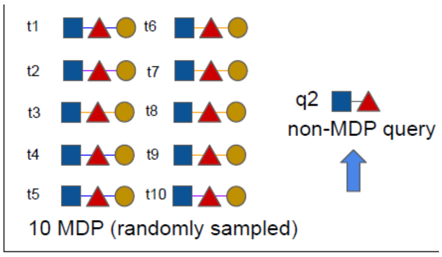

2.2) Check domains present in non-MDP and putatively absent in MDP: there are not

2.3) Check domains present in MDP and putatively absent in the non-MDP query:

2.3.1) For every MDP target (from 1 to 10), if at least the 75% of the protein region coding for the domain has aligned to the non-MDP query: counter +1.

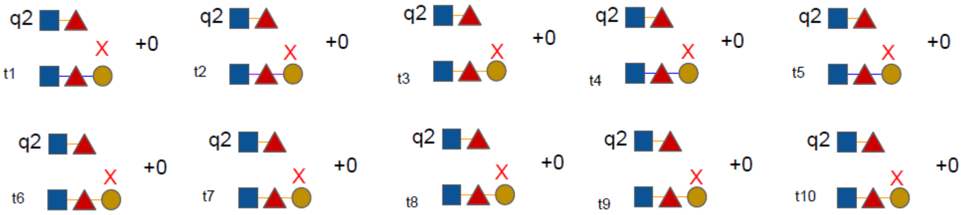

Counter = 0

In this case, as counter is not > 3; q2 is a *bona fide* non-MDP, and hence is <u>not</u> considered as MDP

Supplementary Information 2-Fig. 5D

<u>Split v1</u>

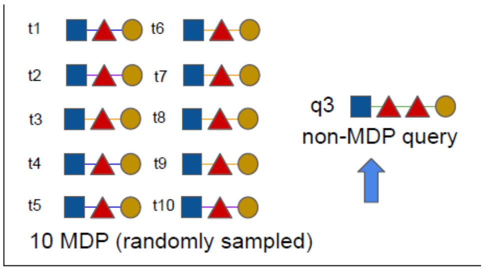

q3 is directly included within MDP, as the only difference with MDP is the presence of an extra copy (could be an internal duplication or a PfamScan missprediction)

Supplementary Information 2-Fig. 5E

Split v1

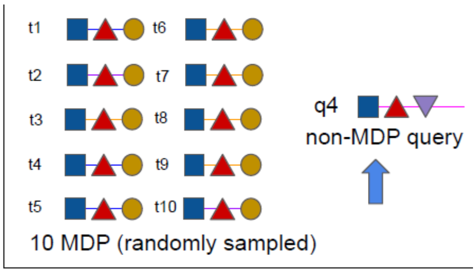

2.2) Check domains present in non-MDP and putatively absent in MDP: ▼

2.2.1) For every MDP target (from 1 to 10), if this protein has aligned to at least the 75% of the non-MDP query region corresponding to the ▼ : counter +1.

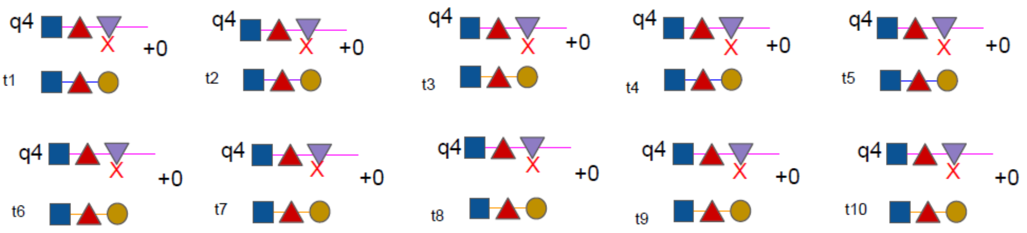

Counter = 0

2.2.2) If, after the 10 iterations, counter > 3, consider that ▼ is present in the non-MDP query despite not being annotated. Else, non-MDP query is **not** a *bona fide* non-MDP

In this case, as counter = 0, the non-MPD is considered as a *bona fide* non-MDP

Supplementary Information 2-Fig. 5F

Split v1

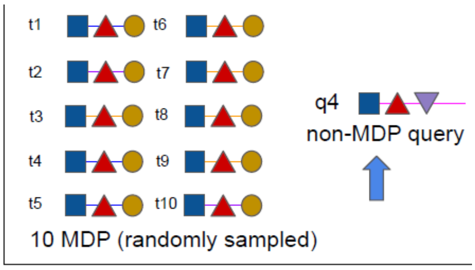

2.3) Check domains present in MDP and putatively absent in non-MDP query: ●

2.3.1) For every MDP target (from 1 to 10), if at least the 75% of the protein region coding for the ● domain has aligned to the non-MDP query: counter +1.

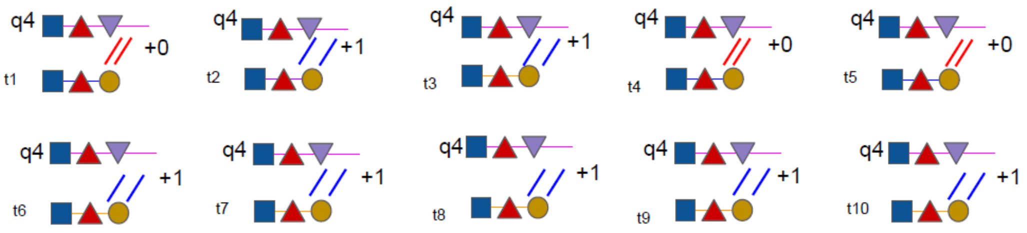

This iteration is skipped by the algorithm, as we already classified q4 as *bona fide* non-MDP (2.2.2 counter was < 4)

Supplementary Information 2-Fig. 5G

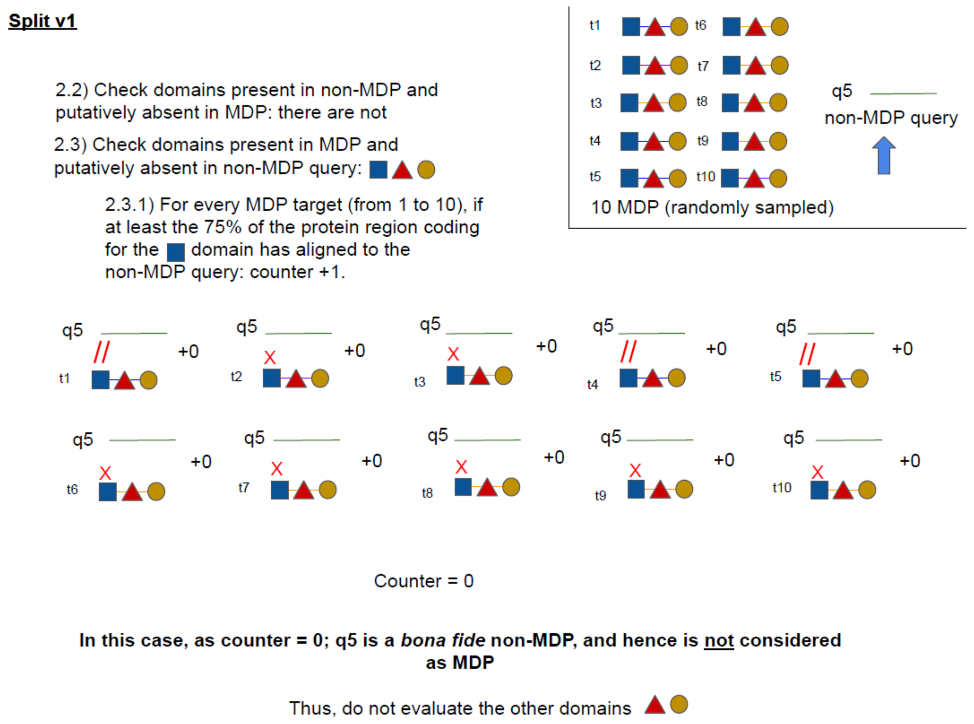

Supplementary Information 2-Fig. 5H

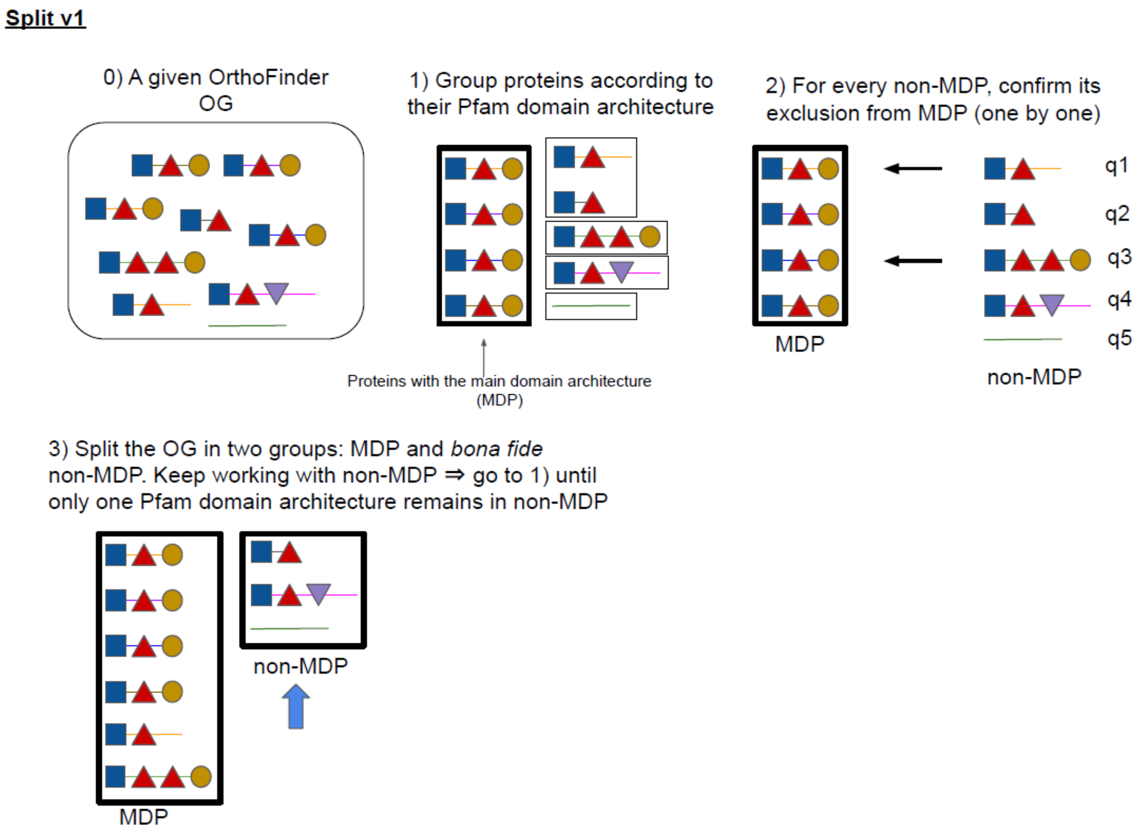

Supplementary Information 2-Fig. 5I

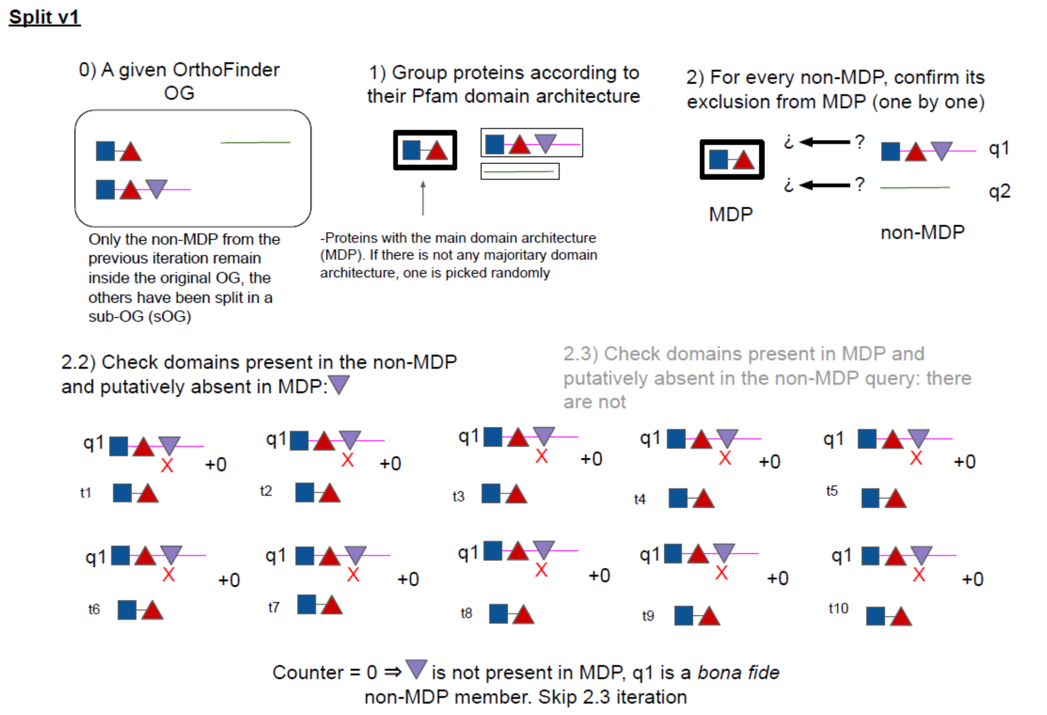

Supplementary Information 2-Fig. 5J

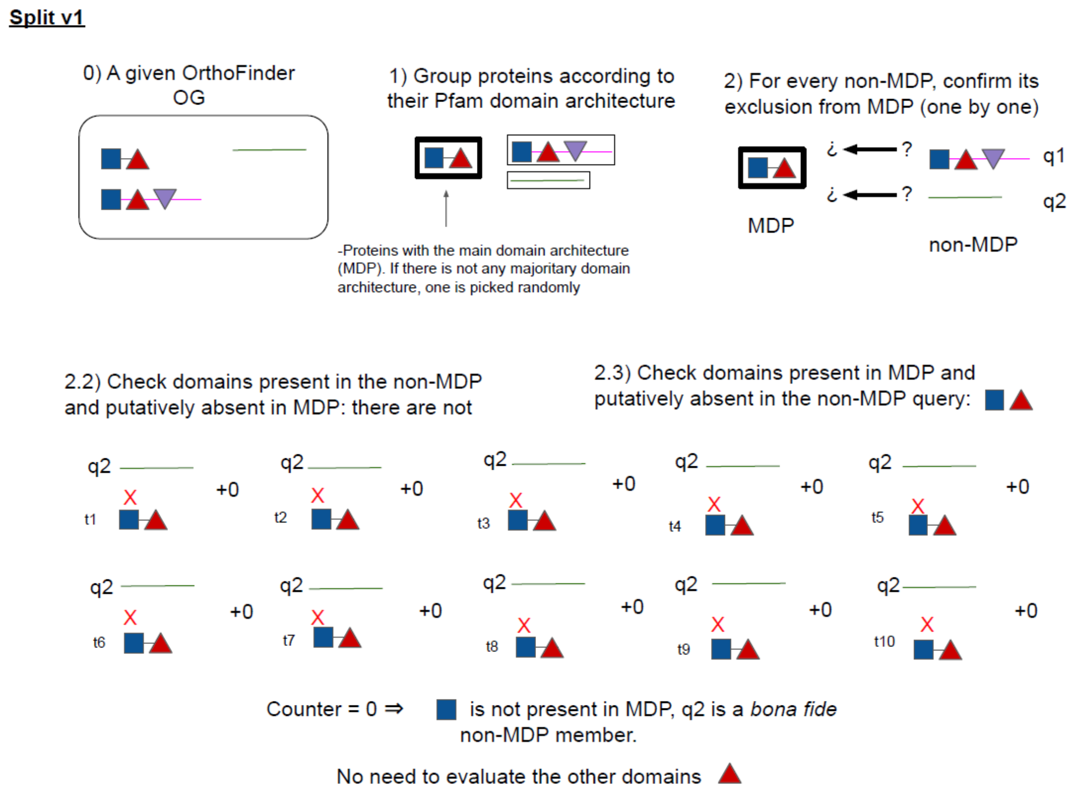

Supplementary Information 2-Fig. 5K

Split v1

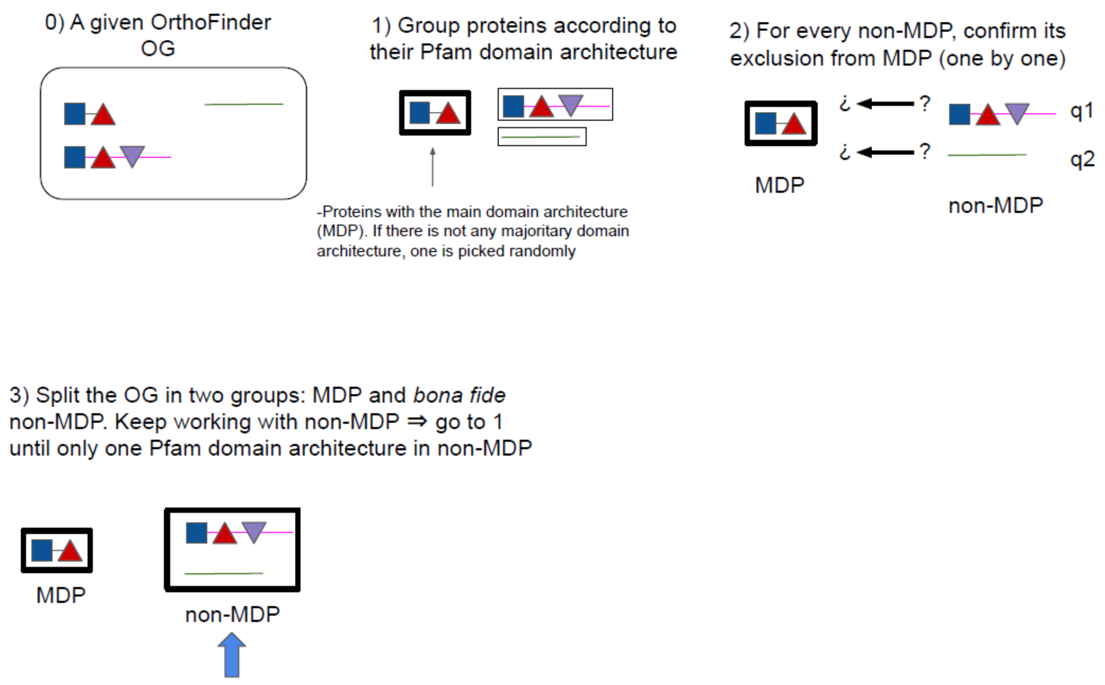

Supplementary Information 2-Fig. 5L

Split v1

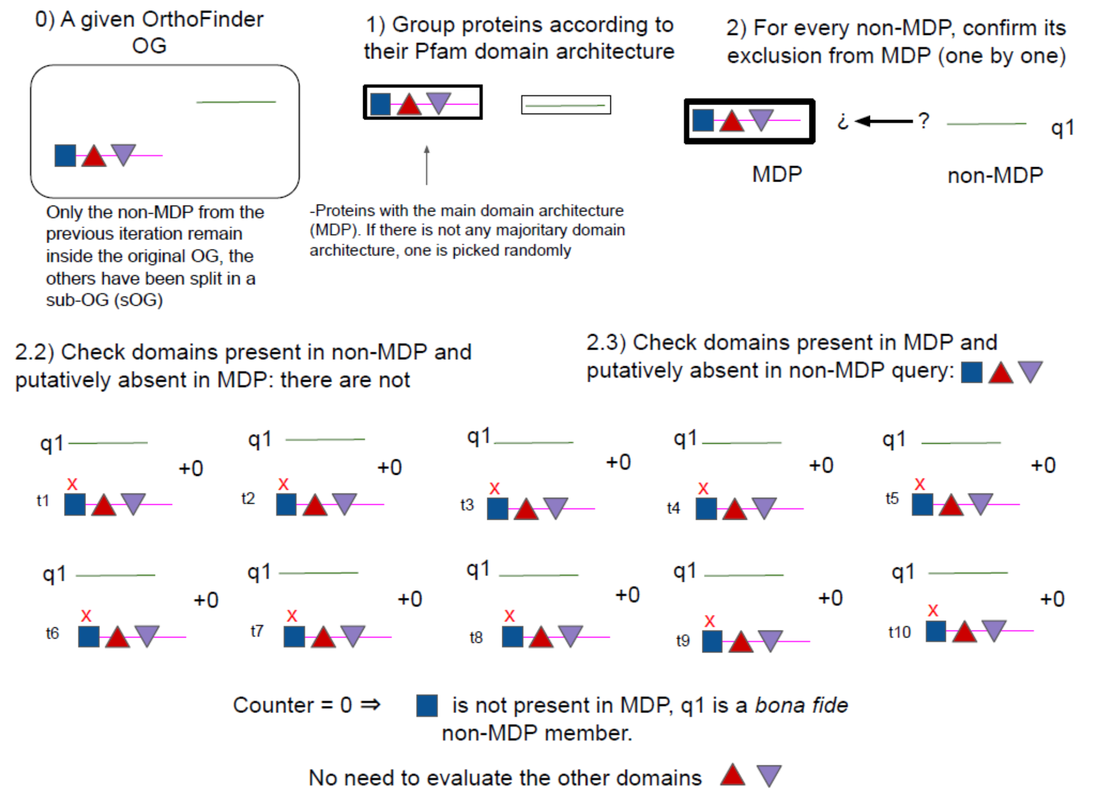

Supplementary Information 2-Fig. 5M

Split v1

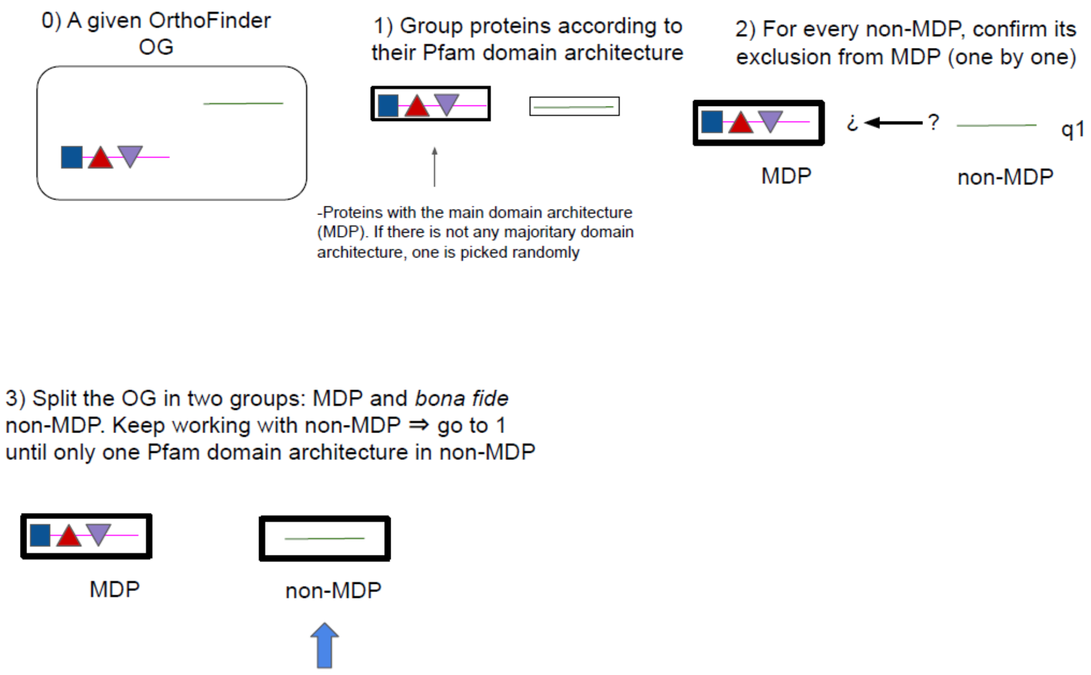

Supplementary Information 2-Fig. 5N

Split v1

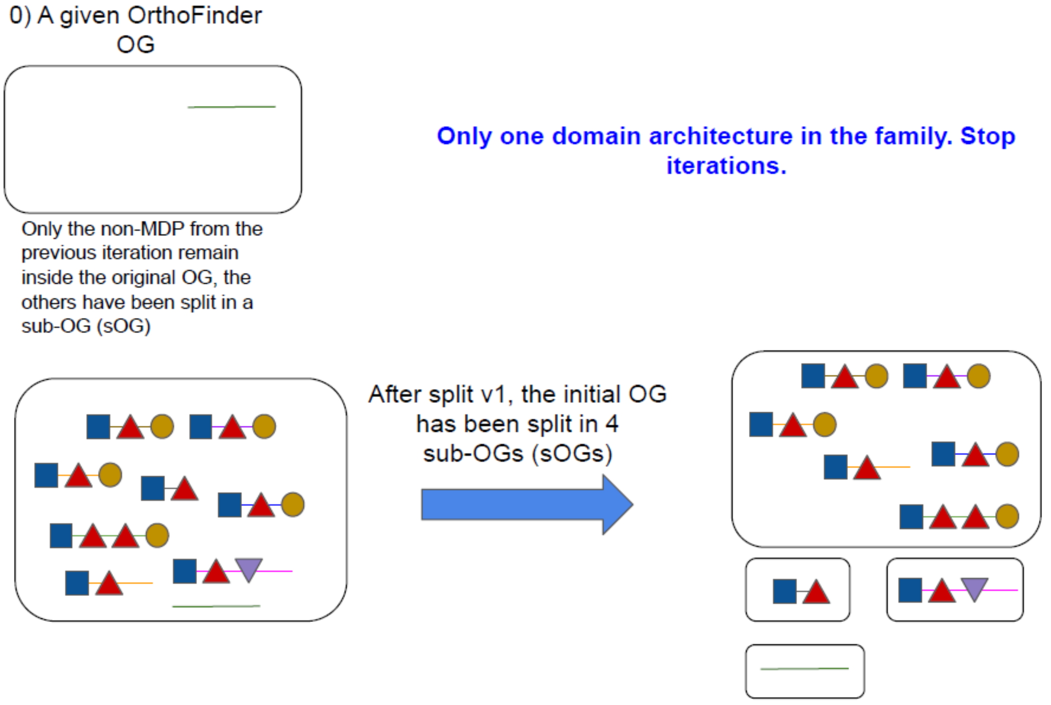

Supplementary Information 2-Fig. 5O

Supplementary Information 2-Fig. 5A-O. Graphical representation of the Split v1 algorithm of *MAPBOS* pipeline.

-Split v2: We found Split v1 to show some limitations with cases of homoplasy at domain architecture level. For example, two proteins from a same family that convergently acquired a given protein domain would be -wrongly- grouped together in a separate sOG even if they are not monophyletic. In order to correct this, Split v1 is always complemented with Split v2. Split v2 checks, for every protein within a given sOG, the protein with which that protein aligned with the best score, and excludes from the sOG those proteins whose best scoring hit is not a protein from the same sOG. Excluded sequences are considered as singletons. The combination of Split v1 and Split v2 will be hereafter referred to as Split v1-2.

-Split v3: We found Split v1-2 to perform well with OGs showing low levels of protein domain heterogeneity (see examples in the following section). However, in OGs with a large diversity of protein architectures, Split v1-2 tends to create an excessive number of singletons. We thus designed an alternative algorithm (Split v3) for these OGs. In particular, Split v3 identifies the protein domain that spans the largest fraction of the sequences within the OG among those domains found in >50% of the OG members, and excludes from the OG those members without the domain. Excluded members are considered as singletons. As with Split v1, the putative absence of the main domain in some members (*PfamScan* results) is validated with the results from *BLASTp* alignments. We created two metrics to decide when Split v3 should be applied instead of Split v1-2. First, Split v3 is applied if more than 25% of members within the original OG have been excluded as singletons after Split v1, as this would suggest either high domain shuffling rates within the family or that many families with distinct architectures have been clustered together (singletons created by Split v1 correspond to sequences showing exclusive architectures within the OG). Second, Split v3 is applied if the number of singletons produced by Split v2 is larger than the 25% of members within the original OG, as this would suggest also high domain shuffling rates or multiple secondary losses of domains (singletons created by Split v2 correspond to sequences that align with a higher score to proteins with a distinct architecture than to proteins with the same architecture). For these heterogeneous OGs, we consider that the usage of Split v1-2 would not be appropriate as these would lead to high levels of singletons and to clusters not correlated with phylogeny (because of homoplasy in domain architecture). Therefore, in these cases, we limited our correction in excluding those members that do not present the main domain in the OG (Split v3). The reason behind the original presence within the OG of the excluded members by Split v3 could be that despite they do not present the main domain in the OG, they could present domains that are co-present with the main domain in the protein architectures of some OG representatives. We found reasonable to exclude them in order to produce OGs in which all members share at least a common homologous region, as this is a requirement of any phylogenetic analyses.

-Split v4 and Split v5: These two alternative split algorithms were created to deal with cases of OG in which there are some proteins with Pfam domains annotated, but none of the domains is found at least in 50% of members. On the one hand, Split v4 is used with OGs in which most of the members have at least one domain annotated. In such a case, the OG is assumed to be a complex assembly of partial homology relationships between subsets of OG members, as otherwise we would expect at least one domain to be present in >50% of the members. The whole OG becomes disassembled and all members are considered to be singletons. On the other hand, Split v5 is used when >50% of the OG members do not have any domain annotated. The absence of domain annotations from most OG members could indicate that the main domain of the OG is not detected by *PfamScan*. In the absence of evidence to determine whether each OG member has the main domain or not, all are considered to have it. In such a case, the members that present some -additional- domains annotated (which correspond to the minority of the OG) are excluded from the OG, as these represent distinct architectures than the rest of the members.

3) Examples of OGs processed by *MAPBOS*

This section includes examples of the behavior of the *MAPBOS* pipeline with 10 non-randomly chosen OGs as well as with 10 OGs that were randomly chosen to represent distinct OG sizes. For each OG, a phylogenetic tree was constructed for all the members included in the original OG (i.e., the output OGs from *OrthoFinder*). Terminal branches of the trees are colored according to the sub-OGs to which each sequence have been classified after the *MAPBOS* pipeline. In particular, each original OG has been divided in as many sub-OGs as colors appear in the represented tree, with the exception of black branches, which represent sequences that the algorithm classified as singletons (i.e., each black branch correspond to a single OG that only includes the represented sequence). Protein domain architectures are also shown (see Material and methods in main text). Figures are available with a higher resolution at https://doi.org/10.6084/m9.figshare.13140191.v1.

a. **10 non-randomly chosen orthogroups**

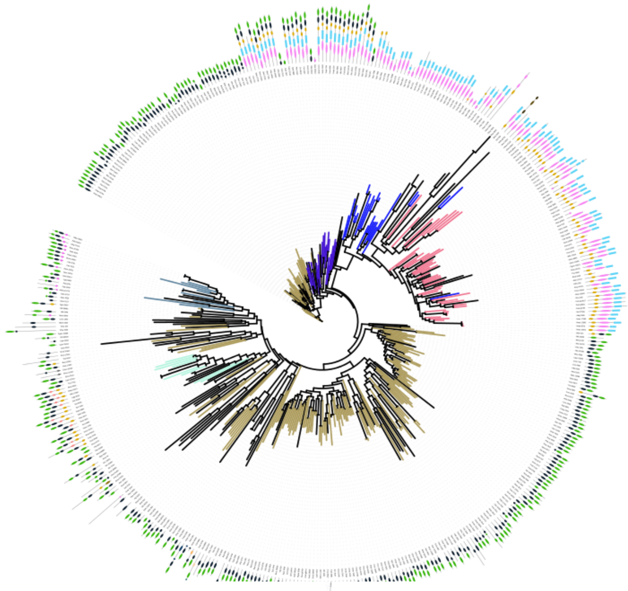

Orthogroup name: OG0000250 (includes the nitrate reductases EUKNR).

Splitting algorithm: Split v1-v2 (because singletons created by Split v1 and by Split v2 were < 25% of the members).

The nitrate reductases (purple, see main text) have been separated from the sulfite oxidases and the FAD/NAD reductases (other colors). The sulfite oxidases and the FAD/NAD reductases also have been split into distinct sub-orthogroups according to differences in domain architecture.

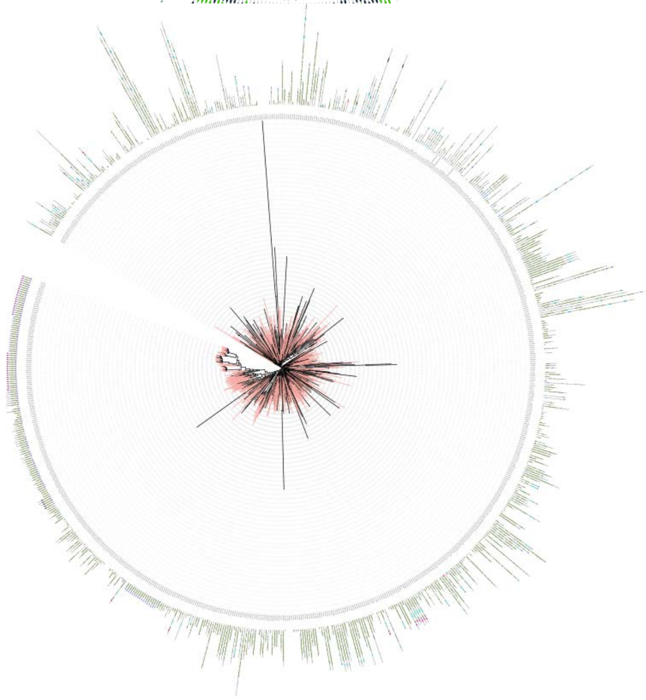

Orthogroup name: OG0000112 (includes cadherins).

Splitting algorithm: Split v3 (because singletons created by Split v2 were > 25% of the members).

According to Split v3 algorithm, all proteins that do not show similarity to the most abundant domain (Cadherin) have been excluded from the orthogroup and are considered as singletons (black branches).

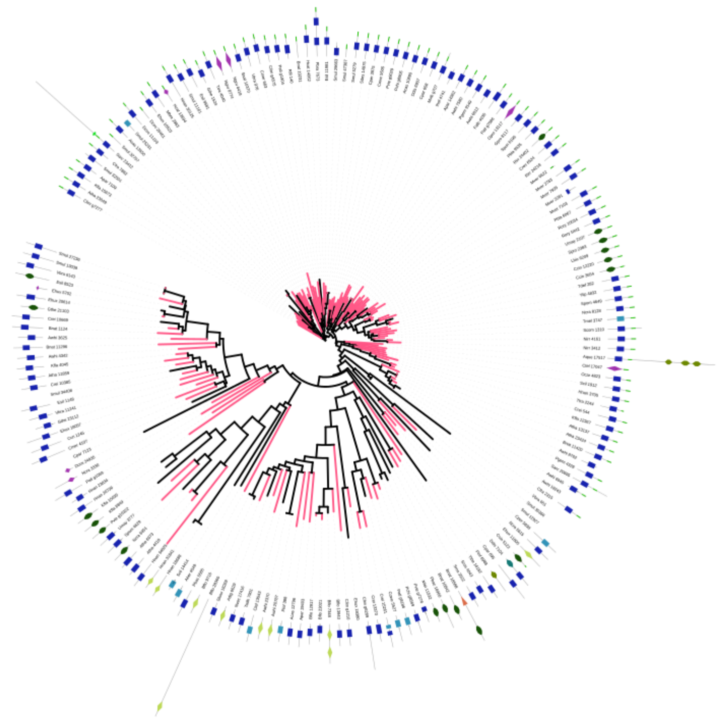

Orthogroup name: OG0000913 (includes sterol methyl transferases).

Splitting algorithm: Split v3 (because singletons created by Split v2 were > 25% of the members).

According to Split v3 algorithm, all proteins that do not show similarity to the most abundant domain (Methyltransf_11, dark blue square) have been excluded from the orthogroup and are considered as singletons (black branches).

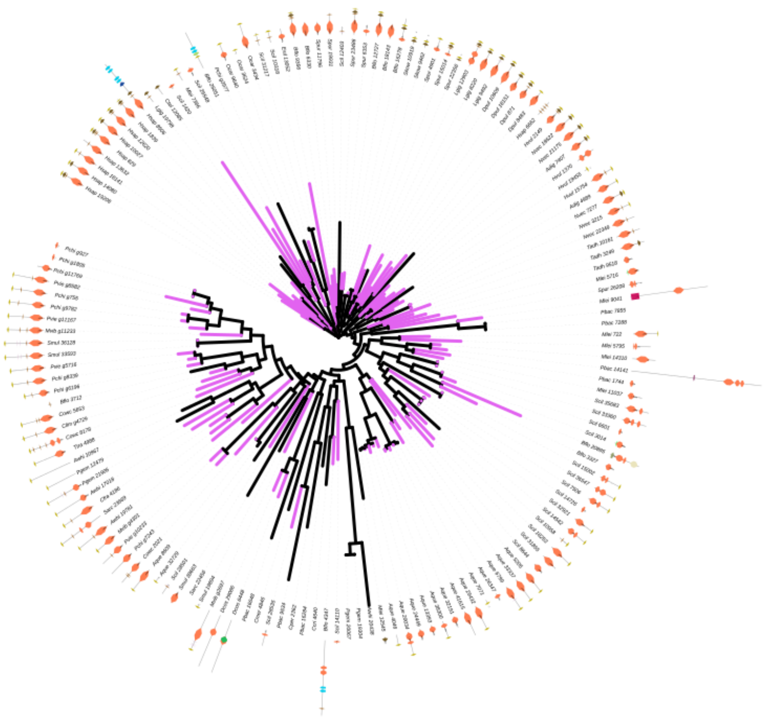

Orthogroup name: OG0001192 (includes integrins).

Splitting algorithm: Split v3 (because singletons created by Split v2 were > 25% of the members).

According to Split v3 algorithm, all proteins that do not show similarity to the most abundant domain (Integrin_beta, orange) have been excluded from the orthogroup and are considered as singletons (black branches).

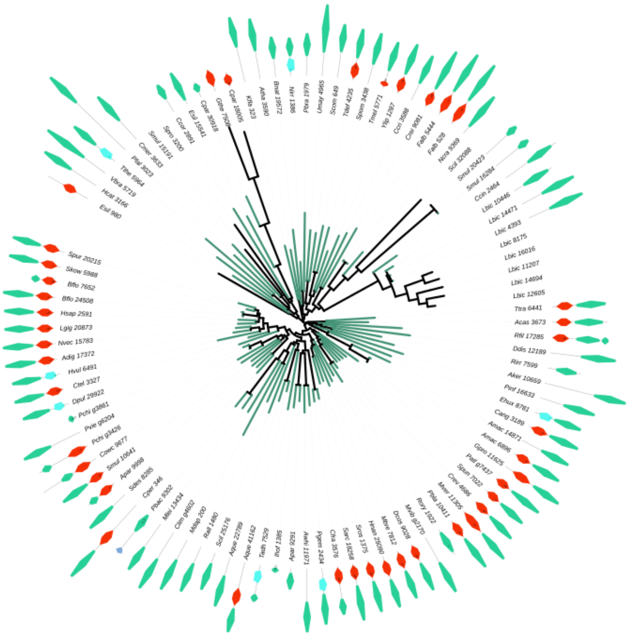

Orthogroup name: OG0002366 (includes ORC5).

Splitting algorithm: Split v3 (because singletons created by Split v2 were > 25% of the members).

According to Split v3 algorithm, all proteins that do not show similarity to the most abundant domain (ORC5_C, green) have been excluded from the orthogroup and are considered as singletons (black branches).

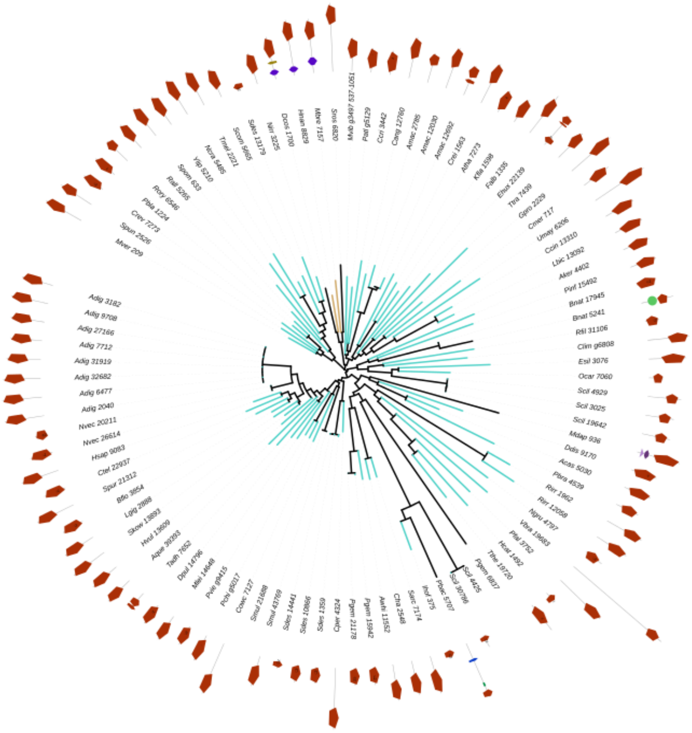

Orthogroup name: OG0002420 (includes ORC2).

Splitting algorithm: Split v1-2 (because singletons created by Split v2 were > 25% of the members).

Most proteins have the same domain architecture (ORC2 monodomain), and hence were put into a same orthogroup. ORC2 proteins from choanoflagellates that incorporated a BAH domain (purple) were clustered together, separate from other ORC2. Other proteins with alternative architectures were considered to be singletons (black branches).

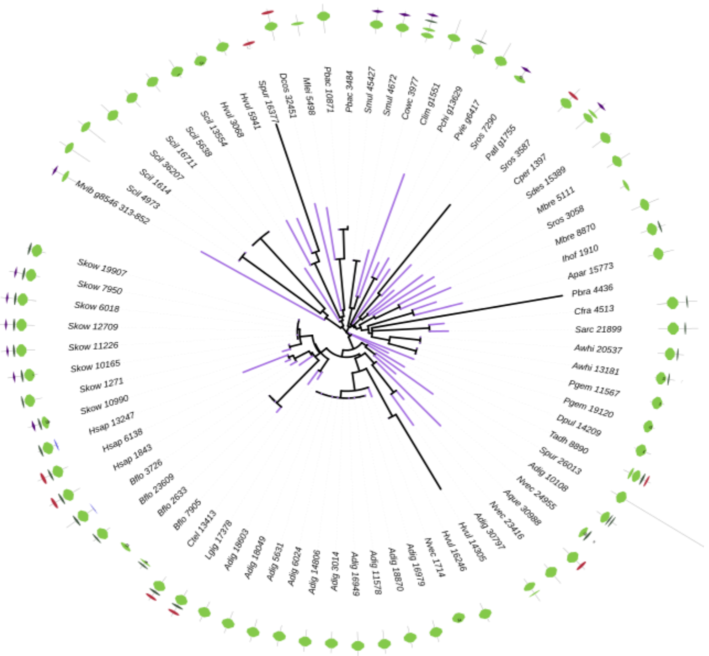

Orthogroup name: OG0003636 (includes P53).

Splitting algorithm: Split v3 (because singletons created by Split v2 were > 25% of the members).

According to Split v3 algorithm, all proteins that do not show similarity to the most abundant domain (P53, green) have been excluded from the orthogroup and are considered as singletons (black branches).

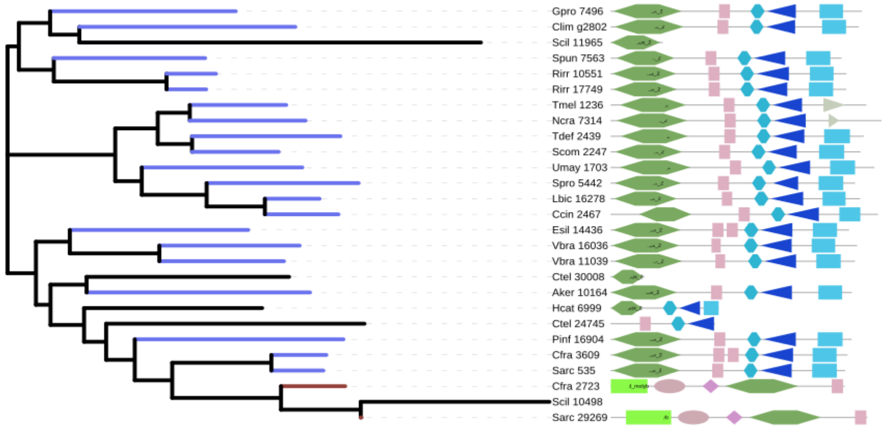

Orthogroup name: OG0007410 (includes NAD[P]H-NIR).

Splitting algorithm: Split v1-2 (because singletons created by Split v1 and by Split v2 were < 25% of the members).

Most of proteins have the same domain architecture and hence were put into a same orthogroup. Two proteins (from *Creolimax fragrantissima* and *Sphaeroforma arctica*) were clustered together in a distinct orthogroup, in agreement with its distinct domain architecture (indeed, they correspond to the CS-pNR sequences that were erroneously clustered together with NAD[P]H-NIR by *OrthoFinder*, see main text). The rest of sequences were considered to be singletons (black branches).

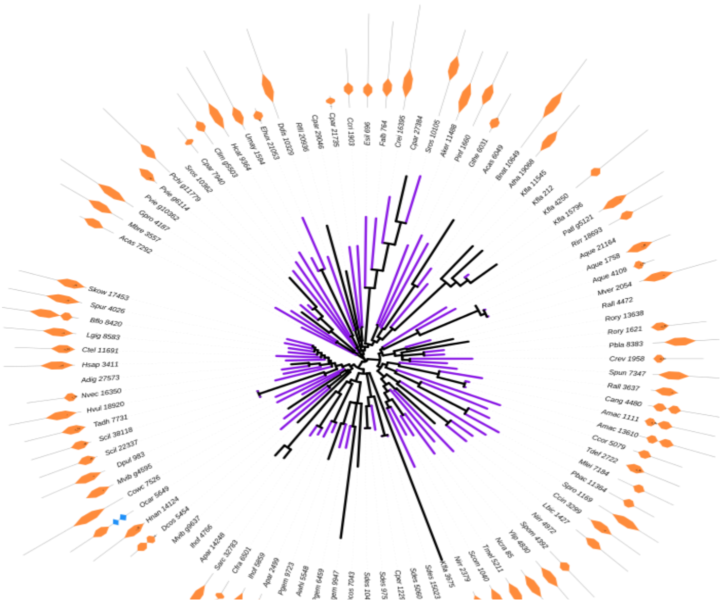

Orthogroup name: OG0002375 (includes ORC3).

Splitting algorithm: Split v1-2 (because singletons created by Split v1 and by Split v2 were < 25% of the members).

Most of proteins have the same domain architecture (ORC3_N monodomain) and hence were included in a same sub-OG. Black branches correspond to singletons, most of which without any domain annotated.

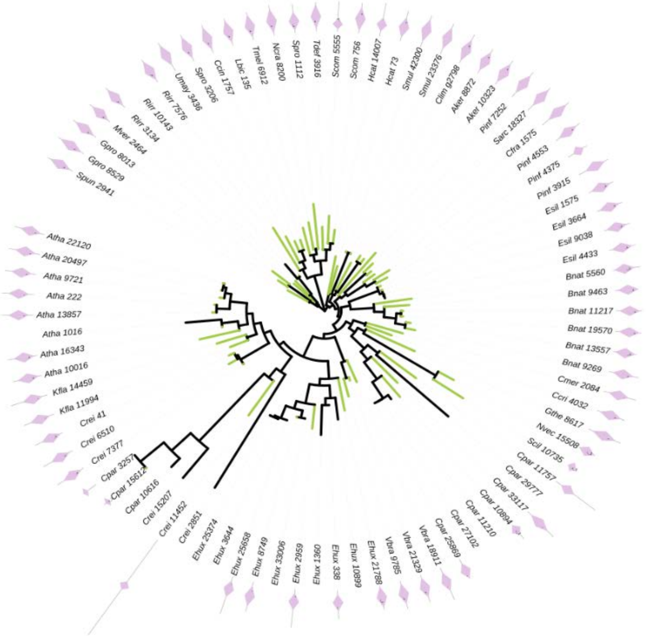

Orthogroup name: OG0003199 (includes NRT2).

Splitting algorithm: Split v1-2 (because singletons created by Split v1 and by Split v2 were < 25% of the members).

Most of proteins have the same domain architecture (MFS_1 monodomain) and hence were included in a same sub-OG. Black branches correspond to singletons, all of which are sequences without any domain annotated.

b. 10 randomly chosen orthogroups

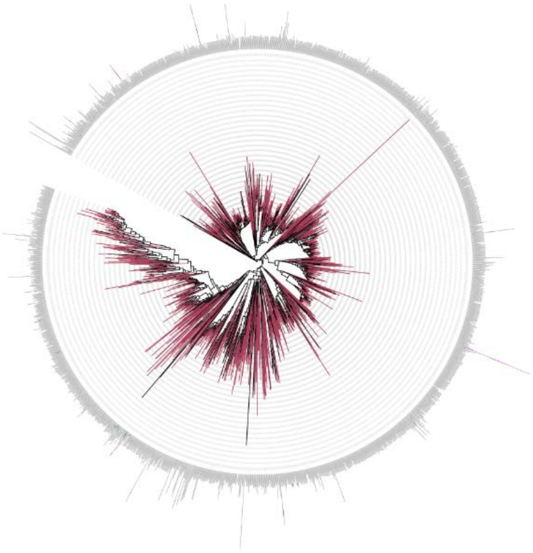

Orthogroup name: OG0000026 (includes Ras sequences). 1701 members.

Splitting algorithm: Split v1-2 (because singletons after Split v1 & after Split v2 < 25% of members).

After Split v2, 39 singletons have been created, with most members still belonging to the same OG.

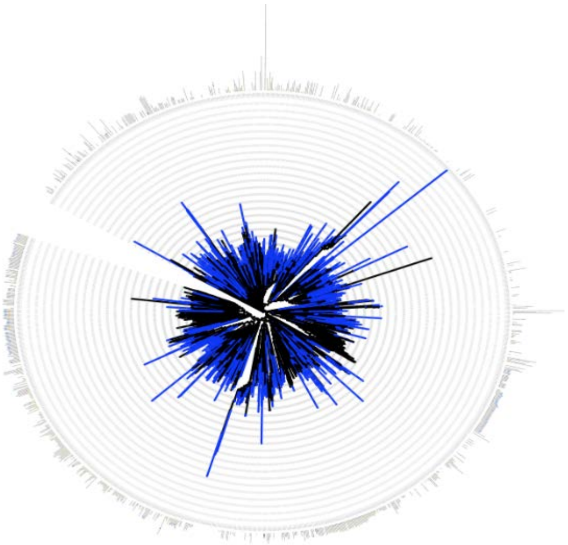

Orthogroup name: OG0000045. 1339 members.

Splitting algorithm: Split v3 (because singletons after Split v2 were 45.18% of members).

Proteins without showing similarity to the most represented domain (LRR_8) were excluded and considered to be singletons (616 singletons).

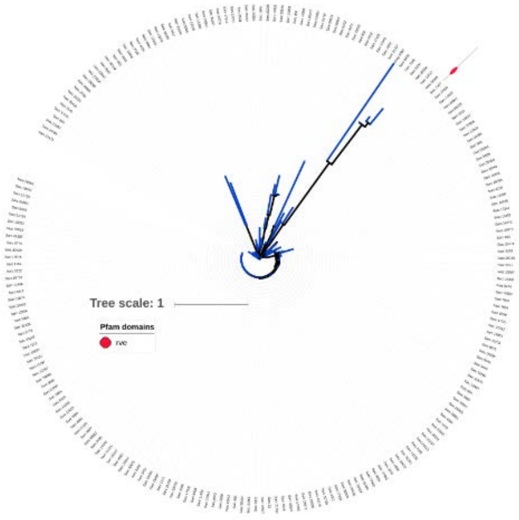

Orthogroup name: OG0000694. 215 members.

Splitting algorithm: Split v5 (because >50% of members do not have any domain annotated).

Only one member has a Pfam domain (rve). This member was excluded from the cluster and considered as singleton. All sequences belong to *Sphaeroforma arctica*.

Orthogroup name: OG0000807. 195 members.

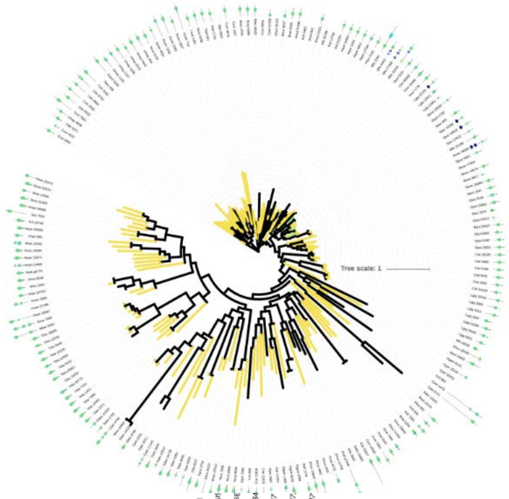

Splitting algorithm: Split v1-2 (because singletons after Split v1 & after Split v2 were < 25% of members).

Apart of singletons, the original OG was split into two sub-OG after Split v1-v2. One includes members with the Glyco_hydro_16 domain (widespread, yellow branches), the other includes members only with the CBM39 and Glyco_hydro_16 domain domains (restricted to 4 sequences from 3 bilaterean taxa, green branches).

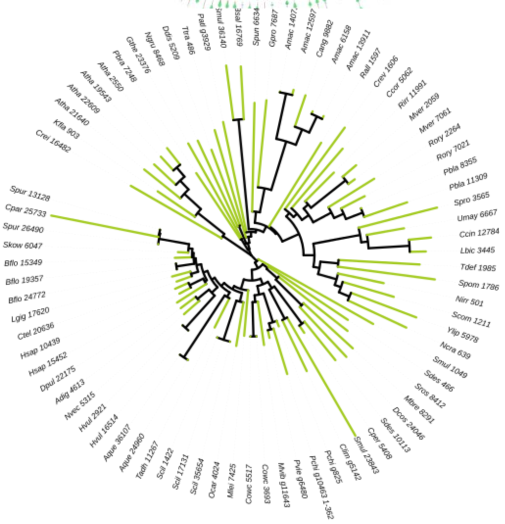

Orthogroup name: OG0003394. 80 members.

Splitting algorithm: Split v0 (because no proteins has a domain annotated).

The orthogroup was not modified because the sequences do not have domains predicted.

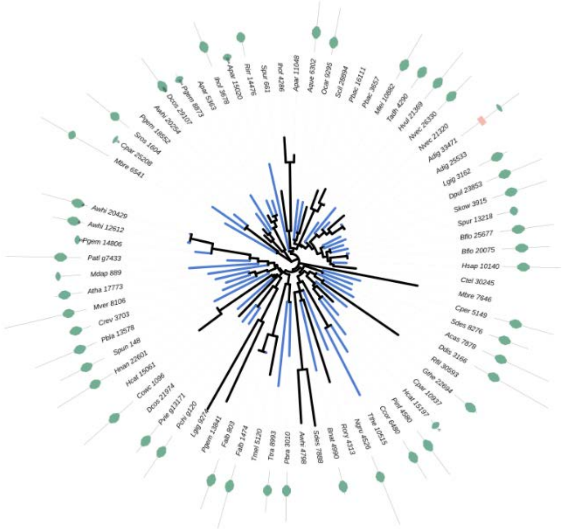

Orthogroup name: OG0003678. 74 members.

Splitting algorithm: Split v3 (because singletons after Split v1 were 31.17% of members).

Most members have the DUF2356 domain (main domain). Proteins not showing similarity to this domain were excluded from the OG.

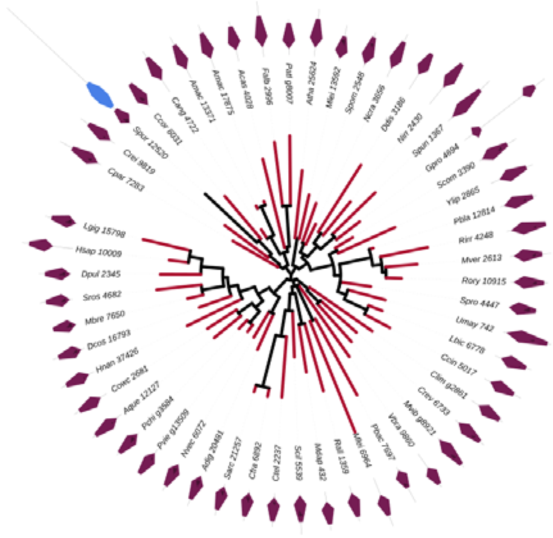

Orthogroup name: OG0004898. 53 members.

Splitting algorithm: Split v1-2 (because singletons after Split v1 & after Split v2 were < 25% of members).

All sequences include the COX6B (purple) domain and were included into the same sub-OG except the one with the WSC domain (blue), which was put in a singleton orthogroup.

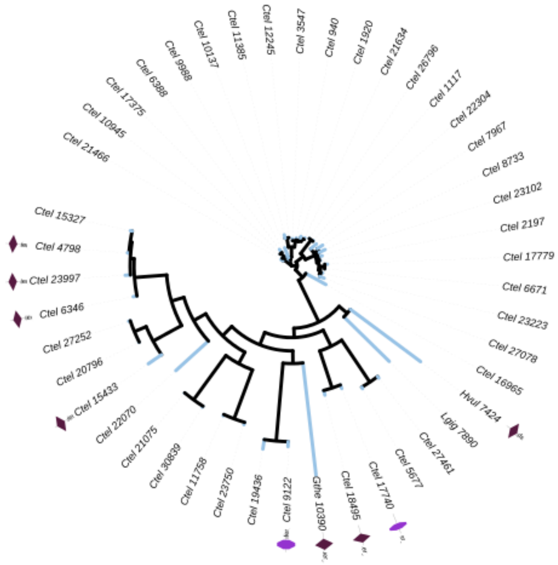

Orthogroup name: OG0005574. 45 members.

Splitting algorithm: Split v5 (because <50% members have a domain annotated).

This is a complex case. Most of the members do not have a domain annotated according to *PfamScan* results. However, some proteins have been annotated with a Sulfotransfer_1 or a Sulfotransfer_2 domain, and the regions of the annotated sequences that correspond to the domains are aligned by all proteins that do not annotations, and also the regions corresponding to the distinct domains align between them. Thus, on the one hand, both domains are considered to be equivalent, and on the other hand, the domain is assumed to be present in all proteins. Because of this, no sequence was finally excluded from the OG.

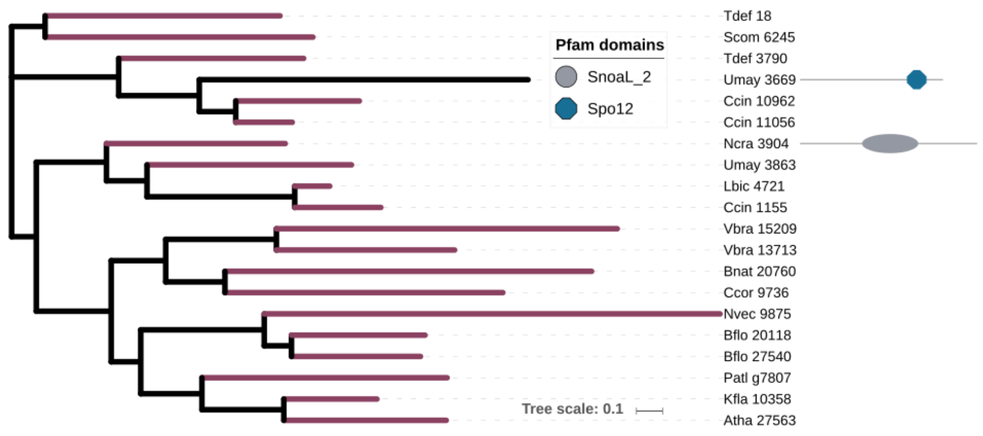

Orthogroup name: OG0008823. 20 members.

Splitting algorithm: Split v5 (because <50% members have a domain annotated).

Most of the members do not have a domain annotated according to *PfamScan* results. The protein with the Spo12 is excluded and considered to be a singleton because the other proteins do not show similarity to the region of the protein corresponding to the Spo12 domain. However, the protein with SnoaL_2 is not excluded because the proteins without domains annotated show similarity to the region corresponding to this domain, and hence it is assumed to be present in all proteins. Because of this, the protein with the SnoaL_2 domain annotated is not considered to be distinct than the other members, and is not excluded.

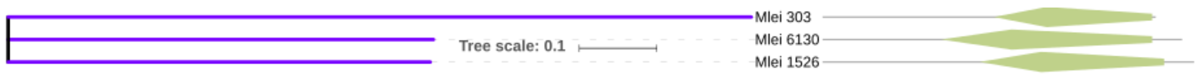

Orthogroup name: OG0033553. 3 members.

Splitting algorithm: Split v0 (because all members have the same domain architecture).

All members were retained because all have the same domain architecture.

4) Analyses of post-*MAPBOS* clusters

The combination of *OF* -I 2 with the *MAPBOS* pipeline (*OF* -I2 *MAPBOS*) overcome all other clustering approaches in the combined metric of sensitivity and precision (Supplementary Information 2-Table 2).

Supplementary Information 2-Table 2. Average sensitivity and precision shown by an extended repertoire of clustering methods, including *OF* -I 2 corrected with the *MAPBOS* pipeline.

Method	Sensitivity	Precision	(S+P) / 2
OF -I2 *MAPBOS*	0.926	0.946	0.936

MCL -I2 mcov50	0.916	0.947	0.9315
OF -I2 mcov50	0.911	0.951	0.931
OF -I2 mcov40	0.938	0.895	0.9165
MCL -I2 mcov60	0.873	0.948	0.9105
OF -I2 mcov60	0.868	0.947	0.9075
MCL -I2 mcov65	0.852	0.956	0.904
MCL -I2 mcov70	0.836	0.955	0.8955
OF -I2 mcov70	0.825	0.964	0.8945
MCL -I2 mcov40	0.949	0.82	0.8845
OF -I2	0.99	0.777	0.8835
MCL -I2	0.987	0.772	0.8795
MCL -I2 mcov75	0.792	0.964	0.878
OF -I2.5	0.974	0.78	0.877
MCL -I2 mcov80	0.73	0.983	0.8565
OF -I3	0.929	0.755	0.842
FD -id10 mcov80	0.695	0.957	0.826
FD -id20 mcov80	0.685	0.964	0.8245
MCL -I1.5	0.99	0.653	0.8215
OF -I1.5	0.99	0.646	0.818
OF -I2 mcov80	0.763	0.763	0.763
FD -id30 mcov80	0.498	0.973	0.7355
FD -id20 mcov60	0.77	0.701	0.7355
FD -id20 mcov50	0.829	0.617	0.723

FD -id20 mcov70	0.716	0.73	0.723
FD -id20 mcov40	0.893	0.239	0.566

We next explored the behavior of *OF* -I2 *MAPBOS* on a broader scale, first by focusing on the protein domain heterogeneity of the clusters (Supplementary Information 2-Fig. 6). As expected, *OF* -I 2 *MAPBOS* present lower heterogeneity at domain architecture than *OF* -I 2 and *MCL* -I 2 in most of cluster size bins. However, the differences observed may be seen as lower than expected. On the one hand, we argue that architecture heterogeneity metrics could have been systematically inflated by cases in which a same domain is present in a variable copy number among members of the same cluster, or by cases in which *PfamScan* annotated homologous regions as if they were distinct domains (e.g., Rieske_2 and Rieske). Clusters in which the heterogeneity is explained by any of these two cases are not split by *MAPBOS*, as neither is indicative of the presence of non-homologous regions between members of the same cluster. In particular, *MAPBOS* only splits clusters when clearly distinct protein domains (i.e., non-homologous regions) are detected among cluster members. By default, *MAPBOS* uses the algorithm Split v1-v2, which splits clusters into sub-clusters, each one representing a distinct domain architecture. This behavior fits with our definition of gene family for this study, in which reticulations between distinct gene families lead to novel composite families[4].

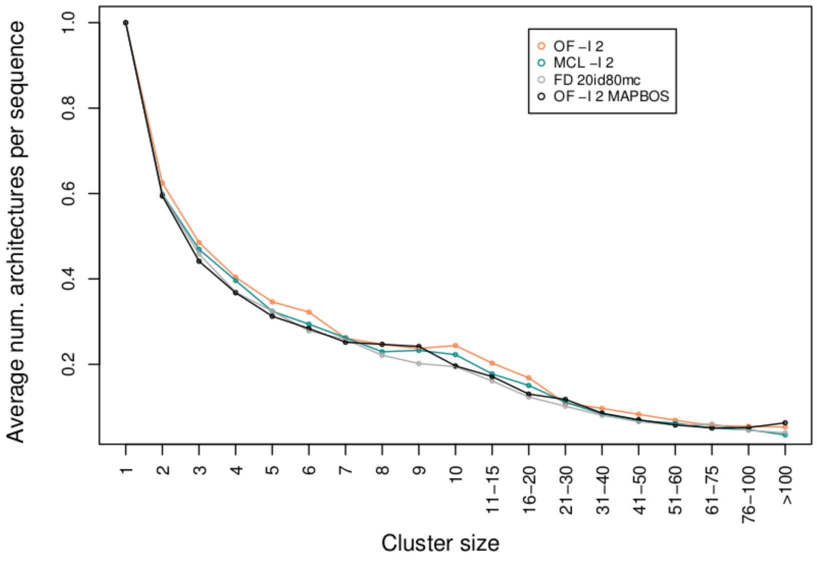

Supplementary Information 2-Fig. 6. Average domain architecture heterogeneity by cluster size in euk_db dataset according to distinct clustering methods. For each cluster the number of distinct domain architectures was counted and divided by the number of members within the cluster. These values were averaged between all clusters of a given size. For example, the value of 'OF -I 2' 0.40 in cluster size 4 in indicates that, on average, the clusters of 4 members show 0.40 architectures per member (i.e., the clusters of size 4 shown on average 1.6 distinct domain architectures). Only clusters in which all members have a Pfam

domain architecture annotated were considered, as for example, a cluster with two members without domain annotations could correspond either to proteins with distinct architectures or to proteins with a same architecture not represented in Pfam database. 'OF -I 1.5': *OrthoFinder* with inflation parameter of 1.5; 'OF -I 2': *OrthoFinder* with inflation parameter of 2; 'MCL -I 1.5': direct *MCL* with inflation parameter of 1.5; 'MCL -I2': direct *MCL* with inflation parameter of 2; 'FD 20id80mc': *FamilyDetector*, 20 % percent identity threshold and 80% of mutual coverage; 'FD 20id40mc': *FamilyDetector*, 20 % percent identity threshold and 40% of mutual coverage. 'OF -I 2 MAPBOS': *OrthoFinder* with inflation parameter of 2 post-processed with the *MAPBOS* pipeline.

Notwithstanding, far beyond the above-mentioned cases that may have inflated architecture heterogeneity metrics, some real heterogeneity may still persist in *OF* -I 2 *MAPBOS* clusters, in particular, in those clusters that were processed with Split v3, which is a less stringent split algorithm than Split v1-v2. *MAPBOS* uses Split v3 instead of Split v1-v2 when the number of singletons produced by Split v1-v2 overcome a given threshold (the threshold value was determined after a manual inspection of the outcomes from Split v1-v2 on distinct clusters). Split v1-v2 produces substantial singletons in clusters that are highly heterogeneous in domain architecture, or when domain architecture is not correlated with phylogeny. Splitting these clusters according to a domain architecture-based criteria, as done by Split v1-v2, would lead to artefactual results. However, instead of leaving them unmodified, we considered beneficial at least the exclusion of those members that do not include the most represented domain in the cluster (Split v3), as these were probably included because of having other domains that co-occur with the most represented domain in the architectures of some cluster members (members excluded from clusters are considered to be singletons). By excluding these members, we at least ensure that clusters are composed of sequences all of which share minimally a common region of homology, this being a requirement of any phylogenetic inference done from sequence alignments, which are hypothesis of positional homology[10]. In total, 16935 clusters were processed with Split v1-v2, whereas 3577 were processed with Split v3 or other more specific split algorithms (Split v4 and Split v5). 245877 of clusters remained unmodified, 224085 of which because of being originally singletons.

There are also two results from the Supplementary Information 2-Fig. 6 that may seem controversial at first glance. On the one hand, for some cluster size bins, *FD* 20id80mc present less architecture heterogeneity than *OF* -I2 *MAPBOS*. This could be explained, as we mentioned above, because *MAPBOS* does not consider a distinct copy number of a given domain *per se* a reason to split a cluster, whereas *FD* 20id80mc indirectly does it due to the strong mutual coverage threshold (80%). On the other hand, clusters with more than 100 members (>100 clusters) appear to be more heterogeneous in *OF* -I2 *MAPBOS* than in *OF* -I2 and *MCL* -I2. This result has a more complex explanation. First, despite *OF* -I2 *MAPBOS* have fewer instances of '>100 clusters' than *OF* -I2 (1302 and 2259), the number of '>100 clusters' from which heterogeneity data could be retrieved was much higher for *OF* -I2 *MAPBOS* (721 and 81). This is

because architecture heterogeneity metrics were only retrieved from clusters in which all members have protein domain information, and post-*MAPBOS* clusters have been cleaned of members without protein domain information. Second, among the 81 clusters with metrics in *OF* -I2, 67 are also among '>100 clusters' in *OF* -I2 *MAPBOS* (the other 14 have <100 members after having been processed by *MAPBOS*). For these 67 clusters, the average domain architecture per sequence in *OF* -I2 is 0.045, almost twice than in *OF* -I2 *MAPBOS* (0.029). Third, beyond these 67 clusters, *OF* -I 2 *MAPBOS* also has metrics for other 654 clusters of >100 members because they were cleaned of sequences without protein domain information. Remarkably, the average nº of architectures per sequence of these 654 clusters is 0.067, more than twice than the other 67 clusters (0.029). This could be related with their members having, on average, more domains per sequence (5.33) than the members of the other 67 clusters (4.76). In conclusion, the worse heterogeneity metrics of *OF* -I2 *MAPBOS* in Supplementary Information 2-Fig. 6 are explained because metrics from the most heterogeneous clusters could not be retrieved in *OF* -I2. Indeed, if we compare the heterogeneity metrics by only considering the 67 clusters that are in the >100 bin before and after *MAPBOS*, *OF* -I 2 *MAPBOS* (0.029) clearly overcomes *OF* -I2 in this metric (0.045).

Because MAPBOS splits clusters into sub-clusters, we next assessed how the distribution of sequences per cluster size was affected with respect to the non-processed clusters (i.e., OF -I 2). As explained in earlier sections, OF and MCL -I 2 produce lesser fragmented clusters than FD id20mcov80 (Supplementary Information 2-Fig. 1), and accordingly, their sensitivity metrics are better than FD id20mcov80 (Supplementary Information 2-Table 1). Compared to FD 20id80mc, the distribution of OF -I2 MAPBOS follows a similar tendency than OF and MCL -I2 (Supplementary Information 2-Fig 7). This suggests that the improvement in precision (Supplementary Information 2-Table 2) was achieved by MAPBOS with a much lesser impact on the fragmentation of the clusters than FD id20mcov80, in agreement with OF -I2 MAPBOS sensitivity values being at the level of OF and MCL -I 2 (Supplementary Information 2-Table 2). Notwithstanding, a major difference between OF -I2 MAPBOS and OF -2 is observed at the level of singletons (>10% more data is found in singleton clusters), which comes at the expense of a lower fraction of the data being distributed in large clusters (Supplementary Information 2-Supplementary Information 2-Fig. 7). We argue that this increment of singletons is overall well justified because they include cases of sequences with distinct architectures than their cluster ex-partners (Split v1-v2), or sequences whose exclusion from the clusters are beneficial because they do not present the most abundant domain in that cluster (Split v3). Accordingly, the proportion of post-MAPBOS new singletons that are detected as composites by *CompositeSearch* is 17.56 times higher (24.81%) than in the original singletons detected by OF -I 2 (1.41%).

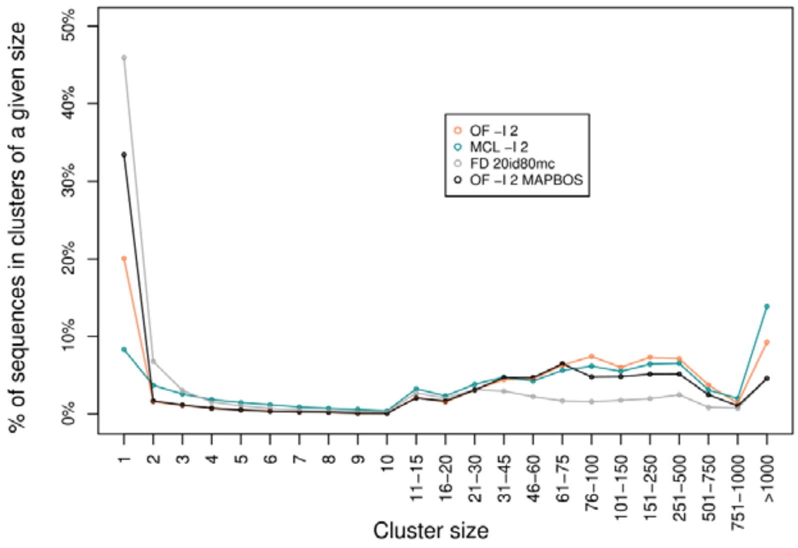

Supplementary Information 2-Fig. 7. Distribution of sequences by cluster size in euk_db dataset according to distinct clustering methods. For example, the ~20% value of 'OF -I2' cluster size 1 indicates that 20% of the sequences within the dataset are in clusters of one single member (i.e., 20% of the sequences are singletons). 'OF -I 1.5': OrthoFinder with inflation parameter of 1.5; 'OF -I 2': OrthoFinder with inflation parameter of 2; 'MCL -I 1.5': direct MCL with inflation parameter of 1.5; 'MCL -I2': direct MCL with inflation parameter of 2; 'FD20id80mc': FamilyDetector, 20 % percent identity threshold and 80% of mutual coverage; 'FD20id40mc': FamilyDetector, 20 % percent identity threshold and 40% of mutual coverage. 'OF -I2 MAPBOS': OrthoFinder with inflation parameter of 2 post-processed with the *MAPBOS* pipeline.

References

1. Enright, A. J., Van Dongen, S. & Ouzounis, C. A. An efficient algorithm for large-scale detection of protein families. *Nucleic Acids Res.* **30**, 1575–1584 (2002).
2. Emms, D. M. & Kelly, S. OrthoFinder: solving fundamental biases in whole genome comparisons dramatically improves orthogroup inference accuracy. *Genome Biol.* **16**, 157 (2015).
3. Paps, J. & Holland, P. W. H. Reconstruction of the ancestral metazoan genome reveals an increase in genomic novelty. *Nat. Commun.* **9**, 1730 (2018).
4. Pathmanathan, J. S., Lopez, P., Lapointe, F.-J. & Bapteste, E. CompositeSearch: A Generalized Network Approach for Composite Gene Families Detection. *Mol. Biol. Evol.* **35**, 252–255 (2017).
5. Ward, N. & Moreno-Hagelsieb, G. Quickly finding orthologs as reciprocal best hits with BLAT, LAST, and UBLAST: How much do we miss? *PLoS One* **9**, e101850 (2014).
6. Corel, E. *et al.* MultiTwin: a software suite to analyze evolution at multiple levels of organization using multipartite graphs. *Genome Biol. Evol.* **10**, 2777–2784 (2018).
7. Ocaña-Pallarès, E., Najle, S. R., Scazzocchio, C. & Ruiz-Trillo, I. Reticulate evolution in eukaryotes: origin and evolution of the nitrate assimilation pathway. *PLoS Genet* **15**, e1007986 (2019).
8. Ocaña-Pallarès, E. *et al.* Origin recognition complex (ORC) evolution is influenced by global gene duplication/loss patterns in eukaryotic genomes. *Genome Biol. Evol.* **12**, 3878–3889 (2020).
9. Haggerty, L. S. *et al.* A pluralistic account of homology: Adapting the models to the data. *Mol. Biol. Evol.* **31**, 501–516 (2014).
10. Rosenberg, M. S. Multiple sequence alignment accuracy and evolutionary distance estimation. *BMC Bioinformatics* **6**, 278 (2005).
