# Supplementary material for: Divergent genomic trajectories predate the origin of animals and fungi
Source: Nature. 2022 Aug 24;609(7928):747–53. doi: 10.1038/s41586-022-05110-4 (PMC9492541; doi:10.1038/s41586-022-05110-4)
Supplement: Supplementary file 5 — Phylogenetic analyses for the species tree reconstruction. [file 41586_2022_5110_MOESM5_ESM.pdf]

Supplementary Information 3

1) Advanced phylogenetic analyses

For the species tree reconstruction (required for the reconciliation analyses), we performed a supermatrix-based maximum likelihood (ML) and Bayesian inference of the Opisthokonta phylogeny (Supplementary Information 3-Fig. 1 and Supplementary Information 3-Fig. 2, respectively, both based on the MCs70 datasets, see Methods). Since we expected our analyses to reproduce uncertainties already reported in the bibliography, we performed a series of phylogenetic analyses in order to explore the differential support for all possible topological resolutions with the aim of setting up a reliable species tree for the reconciliation analyses. First, for both Holozoa and Holomycota MCs70 datasets, we computed 10 additional unconstrained phylogenies, each one progressively removing the 5% of the fastest-evolving sites remaining in the supermatrix. Second, we generated two extra datasets, MCs60 and MCs50, in order to evaluate variations in nodal supports with respect to increments of positions in supermatrix. Both datasets included the OGs in MCs70 but also those with a MCs >0.60 and >0.50, respectively, among the initial set of 342 OGs selected. The newly selected OGs were previously evaluated and filtered as with MCs70 before being incorporated into MCs60 and MCs50 datasets, as we did for those included in MCs70 (see Methods). The number of OGs remaining in MCs60 and MCs50 after the filtering process is 104 and 152, respectively. As with MCs70, we generated two versions for both datasets: Holozoa MCs60 (37 taxa, 27237 sites and 9.65% of missing data), Holomycota MCs60 (28 taxa, 26965 sites and 8.03% of missing data), Holozoa MCs50 (37 taxa, 40845 sites and 10.36% of missing data) and Holomycota MCs50 (28 taxa, 40366 sites and 8.45% of missing data). Nodal supports were retrieved from UFBoot values of ML inferences, and model selection for ML inferences was done using the same criteria as with MCs70. Finally, in order to find potential phylogenetic artifacts related to compositional heterogeneity and/or saturation, we also performed additional ML inferences for Holozoa and Holomycota MCs70, MCs60, and MCs50 datasets [GTR+F+RX] using recoded supermatrices according to Susko and Rogers' four letter alphabet (SR-4)[1]. Alignments and the phylogenetic trees reconstructed are available in the figshare repository ('Alignments_and_trees.zip', https://doi.org/10.6084/m9.figshare.13140191.v1).

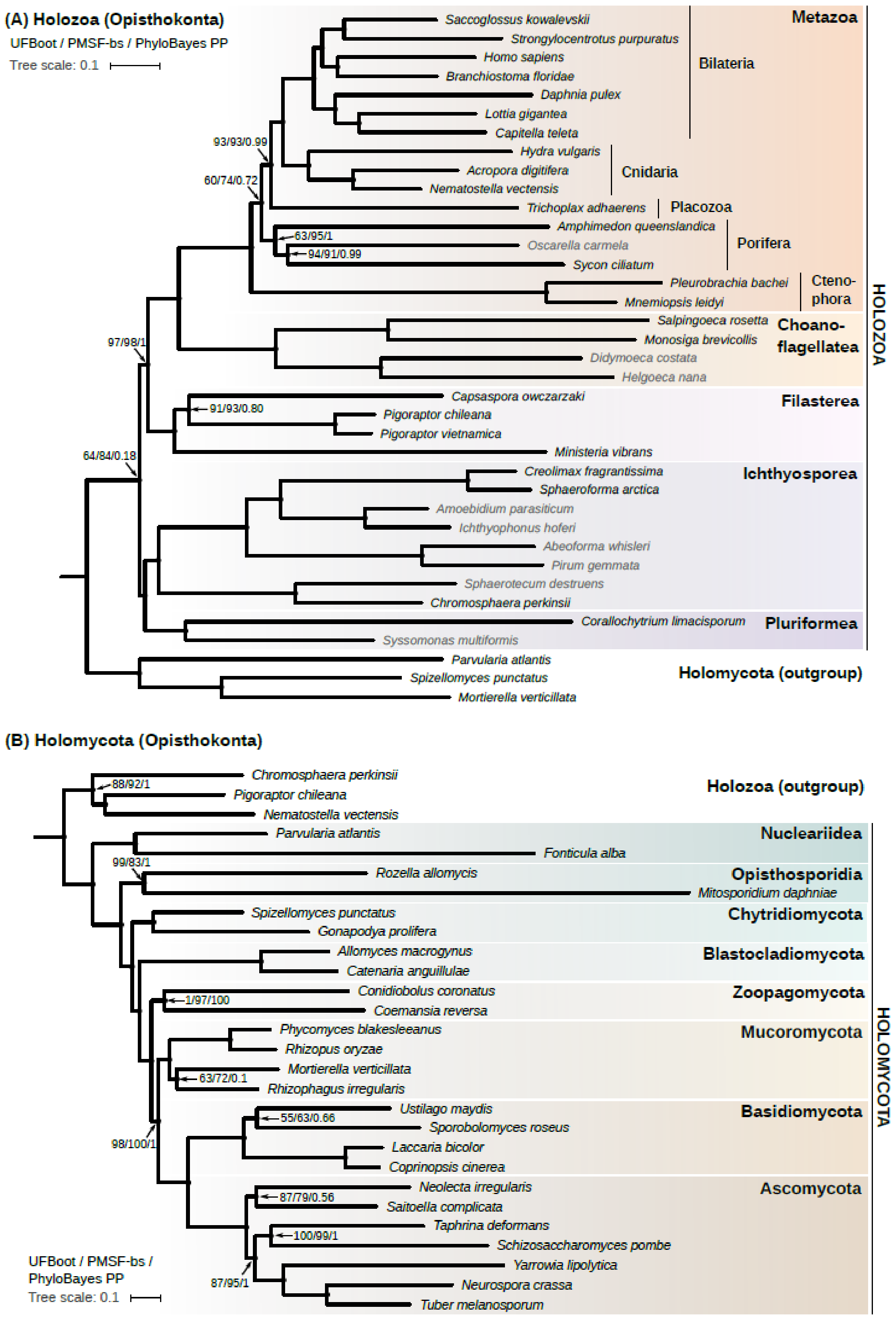

Supplementary Information 3-Fig. 1A-B. Phylogenomic tree of (A) Holozoa and (B) Holomycota, the two clades that together form Opisthokonta, inferred from the corresponding MCs70 supermatrix datasets using the maximum-likelihood IQ-TREE software. Holozoa MCs70 dataset included 17475 sites, and the Holomycota MCs70 dataset included 17409 sites. The models used for the Holozoa and Holomycota MCs70

datasets were, respectively, 'LG+C50+F+R7' and 'LG+C30+F+R6'. Statistical supports are shown only for nodes with support values lower than the maximum possible. UFBoot: maximum likelihood ultrafast bootstrap approximation percentages (1000 replicates; max. value = 100). PMSF-bs: posterior mean site frequency (PMSF) bootstrap percentages (100 normal bootstrap replicates; max. value = 100). PhyloBayes PP: Bayesian posterior probabilities computed under the 'CAT+GTR+Gamma(4)' model (max. value = 1). The taxon names colored in grey correspond to those species whose genomic data was only used for species tree reconstruction purposes (i.e., they were not included in the gene tree species tree reconciliation analyses).

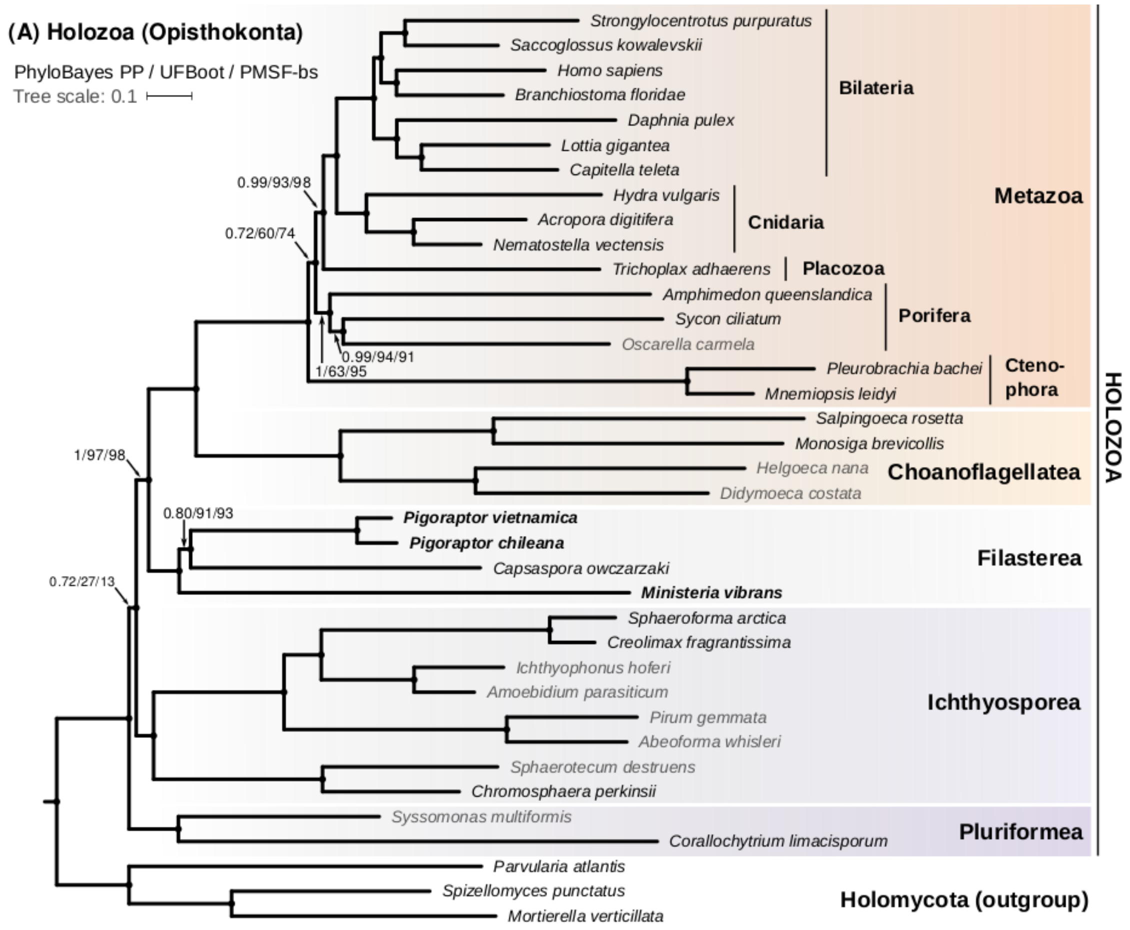

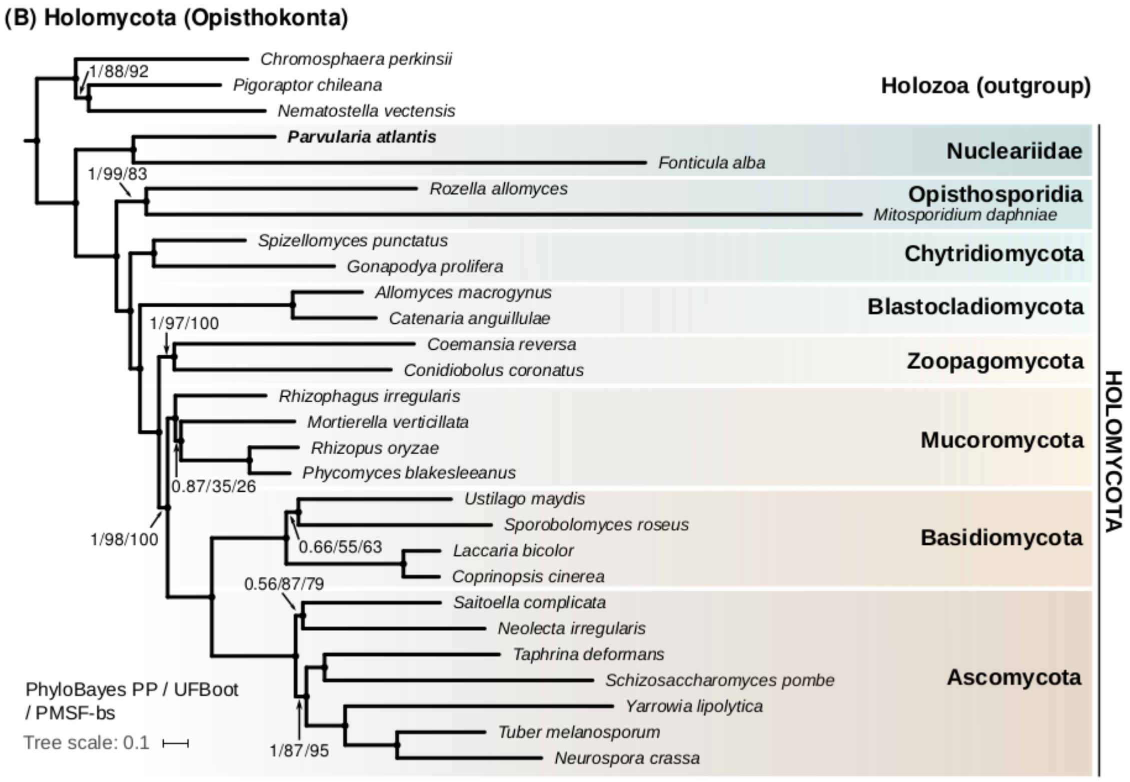

Supplementary Information 3-Fig. 2A-B. Bayesian phylogenomic tree of (A) Holozoa and (B) Holomycota, the two clades in which Opisthokonta divides, inferred from the corresponding MCs70 supermatrix datasets using the PhyloBayes 'CAT+GTR+Gamma(4)'. PhyloBayes PP: Bayesian posterior probabilities computed under the 'CAT+GTR+Gamma(4)' model (max. value = 1). Two chains were run for Holozoa MCs70 and for Holomycota MCs70 supermatrices. Consensus trees were built when the maximum between chain discrepancy in bipartition frequencies fell below 0.1 (burn-in 33%). UFBoot: maximum likelihood ultrafast bootstrap approximation percentages (1000 replicates; max. value = 100). PMSF-bs: posterior mean site frequency (PMSF) bootstrap percentages (100 normal bootstrap replicates; max. value = 100). For ML inferences, the MCs70dataset of Holozoa was inferred under the 'LG+C50+F+R7' model, whereas the MCs70 dataset of Holomycota was inferred under the 'LG+C30+F+R6' model. Statistical supports are shown only for nodes with support values lower than the maximum possible. The taxon names colored in grey correspond to those species whose genomic data was only used for species tree reconstruction purposes (i.e., they were not included in the gene tree species tree reconciliation analyses).

Starting from the uncertainties found within Metazoa, at the phylogenetic scale of this study, there are two major uncertainties. On the one hand, it is uncertain whether Placozoa (here represented by *Trichoplax adhaerens*) is the sister-group of Eumetazoa, as typically recovered by previous phylogenies (e.g., [2,3]), or the sister-group of Cnidaria, as pointed by a recent study[4]. Our ML phylogeny (Supplementary Information 3-Fig. 1) recovered Placozoa+Eumetazoa with almost maximum support, and the support increases as more sites are included in the supermatrix (MCs60 and MCs50 datasets), for both the non-recoded and SR4-recoded datasets (Supplementary Information 3-Fig. 3A). Whereas the support decreases with the progressive removal of the fastest evolving sites, this behaviour is most likely due to a loss of phylogenetic

signal, as the support for Placozoa+Cnidaria (the alternative topology considered) did not increase at the expense of this decrement.

The study that reported Placozoa+Cnidaria[5] incorporated novel placozoan taxa (which in the published tree appear at the terminus of the placozoan branch), and pointed out that the Placozoa+Eumetazoa topology could be a compositional attraction problem. Whereas the support for Placozoa+Bilateria decreased in our compositionally SR4-recoded dataset (Supplementary Information 3-Fig. 3A), the support for Placozoa+Cnidaria was constantly 0%. We thus chose the Placozoa+Bilateria topology for the species tree to be used in the reconciliations. On the other hand, another long-standing uncertainty within Metazoa is whether the earliest lineage of this group is Ctenophora[6] or Porifera[3]. Both ML (Supplementary Information 3-Fig. 1) and Bayesian inferences (Supplementary Information 3-Fig. 2) recovered Ctenophora as sister-group to other Metazoa, and the support for this topology (Ctenophora-early) in both non-recoded and for SR4-recoded datasets increases as more sites are included (Supplementary Information 3-Fig. 3B). As with Placozoa+Bilateria, the support for Ctenophora-early decreases with the removal of fast evolving sites, but again this is more likely to be explained by a loss of phylogenetically informative positions, as the support for the monophyly of Porifera also decreases -unexpected- in proportion to the Ctenophora-early support. In all analyses the support for Porifera-early was much lower than the support for Ctenophora-early, and hence we chose this last topology.

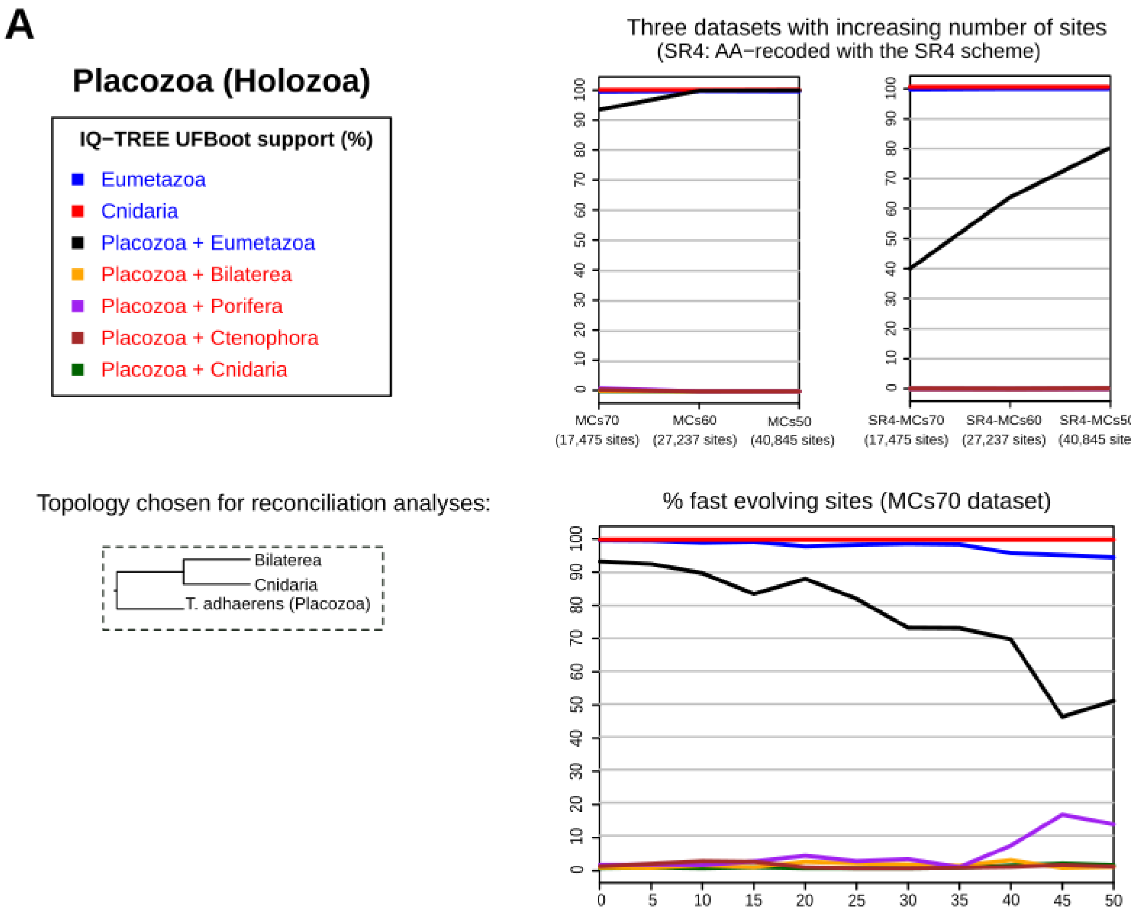

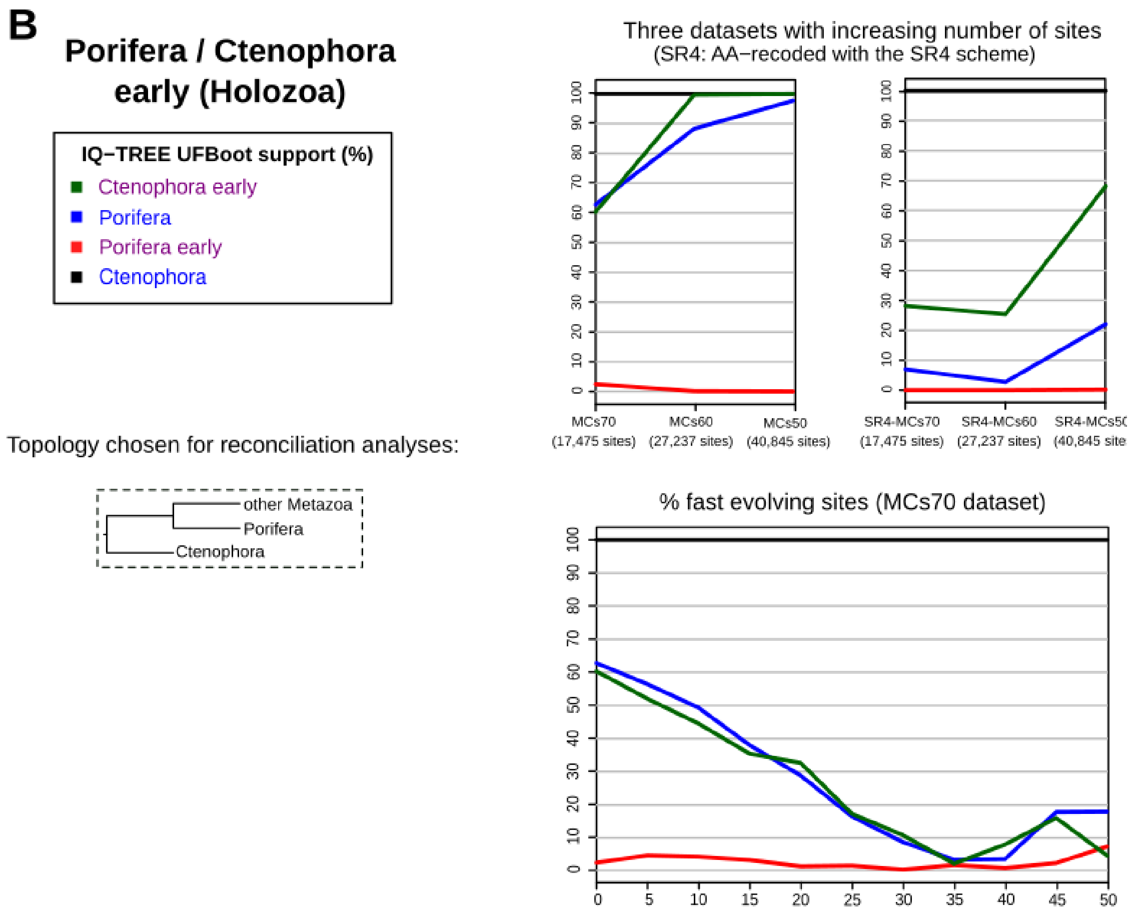

Supplementary Information 3-Fig. 3. Phylogenetic analyses done to evaluate the support for a series of potential topologies related to two conflicting scenarios within Metazoa: (**A**) the position of Placozoa with respect to Eumetazoa or Cnidaria and (**B**) the position of Ctenophora or Porifera as the earliest metazoan lineage. Three distinct analyses were done. The first analysis consisted in measuring the UFBoot % support of all topologies evaluated under three datasets with distinct sizes. The second analysis is similar to the first but using a recoded version of the MCs70, MCs60 and MCs50 datasets according to Susko and Rogers' four letter alphabet (SR-4). The third analyses consisted in measuring the UFBoot % support of all topologies under the progressive removal of the 5% of the fastest-evolving sites remaining in the supermatrix (see Methods). The topologies evaluated for every scenario correspond to those shown in the top left panels. Topologies colored in blue correspond to those that were confidently accepted after the analyses (except for Porifera, which is colored in blue because the monophyly of this group is well established in the bibliography), in red those that were confidently rejected, and in purple those topologies that could not be neither confidently accepted nor rejected. The final topology chosen for each scenario is shown in the bottom panels.

At the holozoan level, there are two uncertainties in our phylogeny. On the one hand, whereas the positions of Filasterea as sister-group to Choanozoa (choanoflagellates+Metazoa) is well established, the topology within Filasterea is more uncertain. Our ML and Bayesian phylogenies recovers *C. owczarzaki* as sister-group to both *Pigoraptor* species, with *M. vibrans* being the earliest-branching filasterean lineage (Supplementary Information 3-Fig. 1). However, the *C. owczarzaki+Pigoraptor* clade is not fully supported, as it was not in [7]. Whereas *C.*

owczarzaki+*Pigoraptor* is the best supported topology in any of the non-recoded phylogenies (Supplementary Information 3-Fig. 4A), this topology is the least supported one in the SR4-recoded datasets. In particular, in the SR4-recoded datasets, *C. owczarzaki*+*M. vibrans* is the preferred topology. However, given that neither this nor any other topology is highly supported in SR-4 trees, we finally chose the *C. owczarzaki*+*Pigoraptor* topology. On the other hand, another recent uncertainty in Holozoa is the position of the *C. limacisporum* and *S. multiformis* clade (referred to as Pluriformea in [7]) with respect to the rest of Holozoa. Whereas the Pluriformea+Filasterea+Choanozoa clade appeared as the most supported topology in [7], we recovered a minimum support for this topology in our analyses (Supplementary Information 3-Fig. 4B). In contrast, our trees recovered more support for two alternative topologies: (1) Pluriformea+Ichthyosporea and (2) Pluriformea+Holomycota. In particular, the ML tree in Fig. 4A and the fastest evolving sites removal analyses show preference for the first topology (Supplementary Information 3-Fig. 4B), whereas the second topology is better supported in the Bayesian tree (Supplementary Information 3-Fig. 2) and in the dataset with an increased number of sites (MCs60 and MCs50, for both recorded and non-recoded datasets, Supplementary Information 3-Fig. 4B). In the face of this controversy, we finally considered the first topology, given that it was strongly supported in an Opisthokonta phylogeny reconstructed in [8].

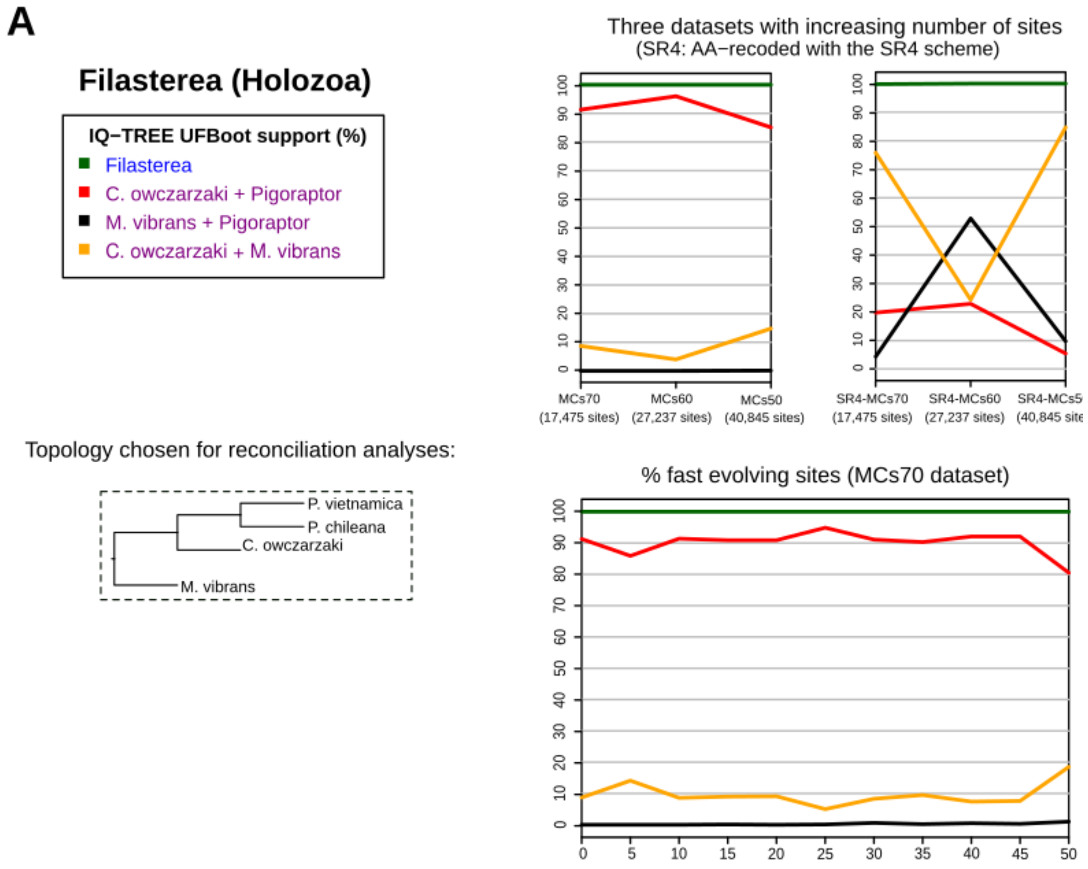

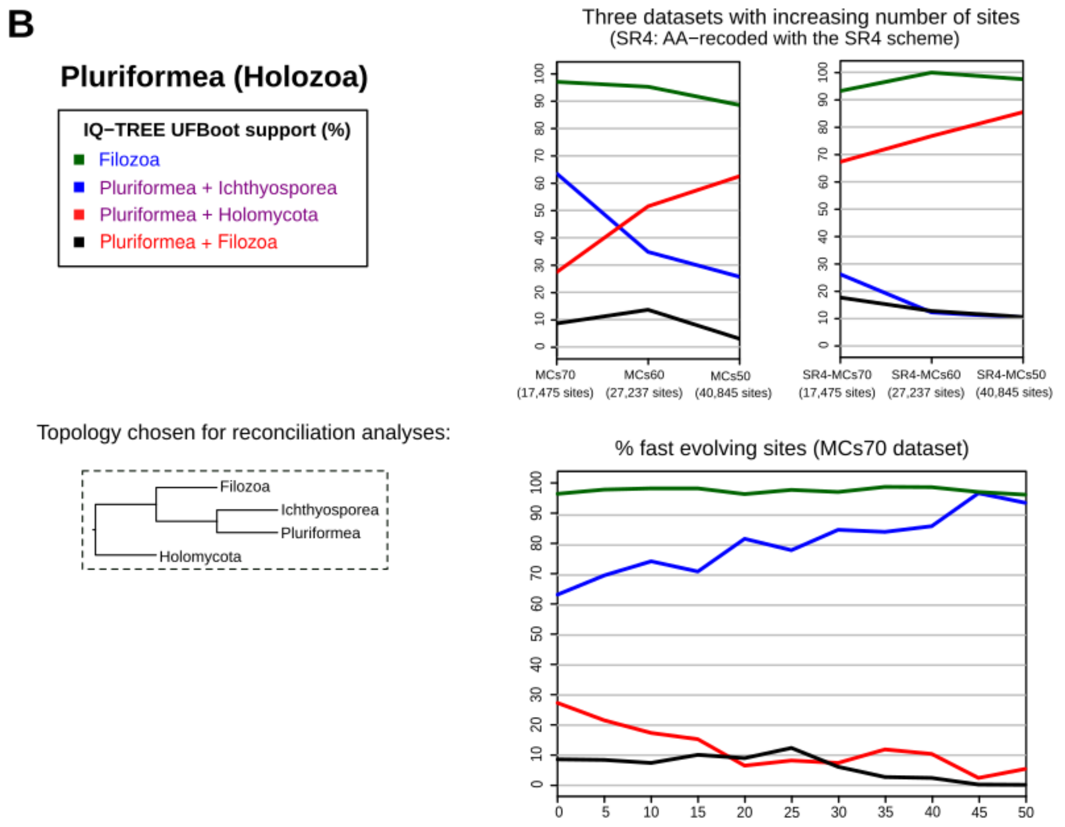

Supplementary Information 3-Fig. 4. Phylogenetic analyses done to evaluate the support for a series of potential topologies related to two conflicting scenarios within Holozoa: (**A**) the position of *Ministeria vibrans* with respect to the other filastereans and (**B**) the position of Pluriformea respect to the other opisthokonts. Three distinct analyses were done. The first analysis consisted in measuring the UFBoot % support of all topologies evaluated under three datasets with distinct sizes. The second analysis is similar to the first but using a recoded version of the MCs70, MCs60 and MCs50datasets according to Susko and Rogers' four letter alphabet (SR-4). The third analyses consisted in measuring the UFBoot % support of all topologies under the progressive removal of the 5% of the fastest-evolving sites remaining in the supermatrix (see Methods). The topologies evaluated for every scenario correspond to those shown in the top left panels. Topologies colored in blue correspond to those that were confidently accepted after the analyses, in red those that were confidently rejected, and in purple those topologies that could not be neither confidently accepted nor rejected. The final topology chosen for each scenario is shown in the bottom panels.

On the holomycotan side, we evaluated two uncertainties underlying the phylogenetic relationships between the sampled pre-dikaryotic fungal lineages. On the one hand, it is uncertain which is the earliest lineage of the Fungi *sensu stricto* clade (i.e., excluding Opisthosporidia [9]). Although phylogenies tend to recover Blastocladiomycota-early, generally with low support (e.g., [9,10]), our ML (Supplementary Information 3-Fig. 1) and Bayesian phylogenies (Supplementary Information 3-Fig. 5A) recovered Chytridiomycota-early, and this topology was consistently recovered with high support in all analyses (Supplementary Information 3-Fig. 5A). We thus considered the Chytridiomycota-early topology, although further sampling would be needed to confidently solve this major uncertainty in early fungal diversification. We also found uncertainty within the Mucuromycota clade, in particular, regarding the position of *Mortierella verticillata* and

Rhizophagus irregularis respect to *Phycomyces blakesleeanus* and *Rhizopus oryzae*. In particular, *M. verticillata*+*R. irregularis* appeared as sister-group to *P. blakesleeanus*+*R. oryzae* in the ML tree (Supplementary Information 3-Fig. 1), but this topology was poorly supported in the Bayesian tree (Supplementary Information 3-Fig. 5B). Despite the uncertainty, the *M. verticillata*+*R. irregularis* topology was finally chose as it was more supported than *P. blakesleeanus*+*R. oryzae*+*R. irregularis* and *P. blakesleeanus*+*R. oryzae*+*M. verticillata* in most of the phylogenetic analyses done (Supplementary Information 3-Fig. 5B), with the last topology being poorly supported in all of them.

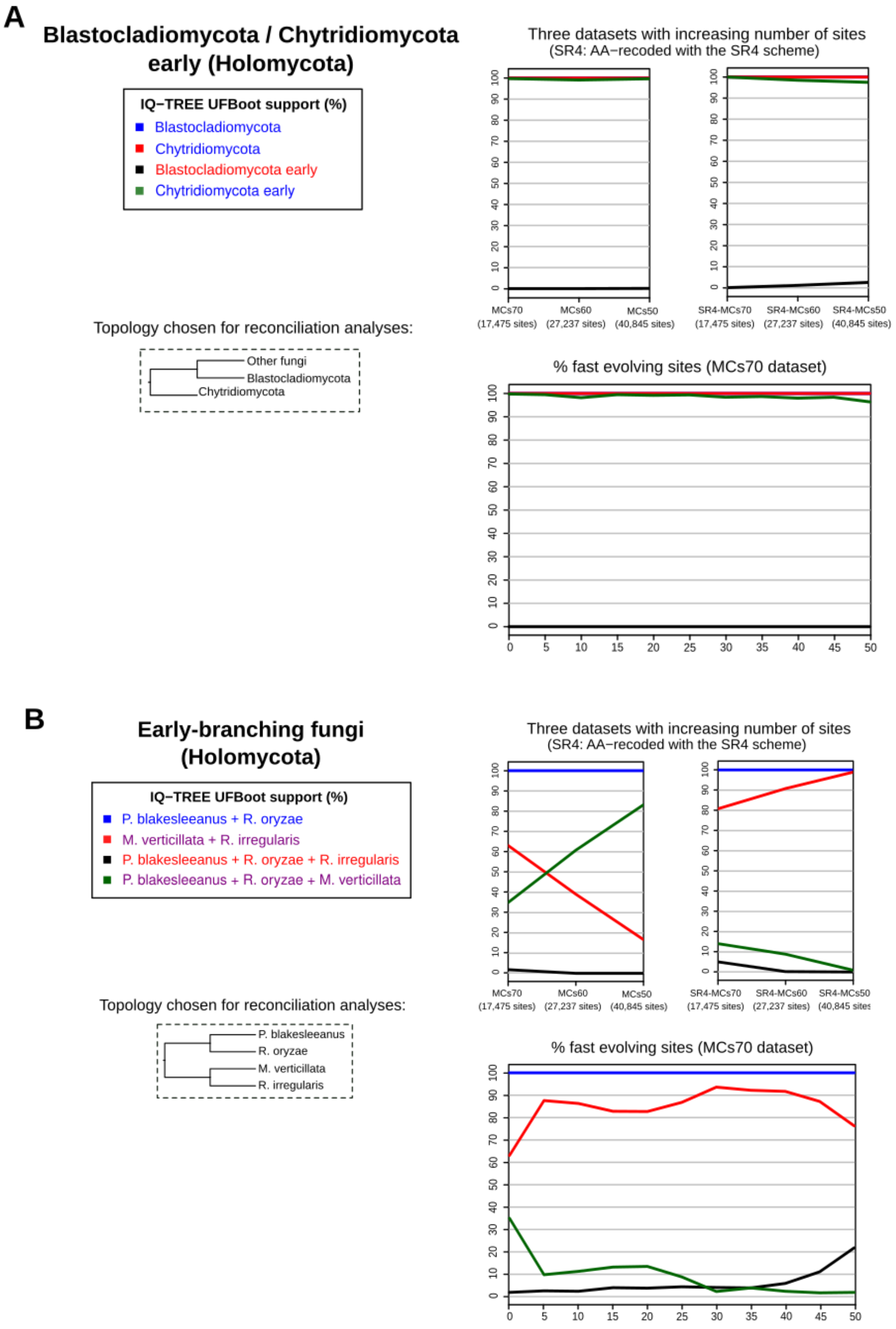

Supplementary Information 3-Fig. 5. Phylogenetic analyses done to evaluate the support for a series of potential topologies related to two conflicting scenarios within Holomycota: (**A**) the earliest branching lineage of the fungal clade (either Chytridiomycota or Blastocladiomycota) and (**B**) the internal phylogeny of Mucoromycota. Three distinct analyses were done. The first analysis consisted in measuring the UFBoot % support of all topologies evaluated under three datasets with distinct sizes. The second analysis is similar to the first but using a recoded version of the MCs70, MCs60and MCs50 datasets according to Susko and Rogers' four letter alphabet (SR-4). The third analyses consisted in measuring the UFBoot % support of all topologies under the progressive removal of the 5% of the fastest-evolving sites remaining in the supermatrix (see Methods). The topologies evaluated for every scenario correspond to those shown in the top left panels. Topologies colored in blue correspond to those that were confidently accepted after the analyses, in red those that were confidently rejected, and in purple those topologies that could not be neither confidently accepted nor rejected. The final topology chosen for each scenario is shown in the bottom panels.

Our phylogenies also recovered phylogenetic uncertainties within Dikarya. On the one hand, within Basidiomycota, the position of *Ustilago maydis* and *Sporobolomyces roseus* is uncertain with respect to clade formed by *Laccaria bicolor* and *Coprinopsis cinerea*. The *U. maydis*+*S. roseus* topology is the most supported in both ML (Supplementary Information 3-Fig. 1) and Bayesian trees (Supplementary Information 3-Fig. 2), as well as in all phylogenetic analyses shown in Supplementary Information 3-Fig. 6A, and hence we chose this one rather than the others. On the other hand, there are two uncertainties within Ascomycota: a first one concerning the position of *Saitoella complicata* and *Neolecta irregularis* with respect to other taxa; and a second one concerning the position of *Tuber melanosporum*+*Neurospora crassa*+ *Yarrowia lipolytica* clade with respect to *Taphrina deformans*+*Schizosaccharomyces pombe* clade. We finally considered the topology shown by both ML and Bayesian trees, as these was the overall most supported one in the analyses done (Supplementary Information 3-Fig. 6B).

A

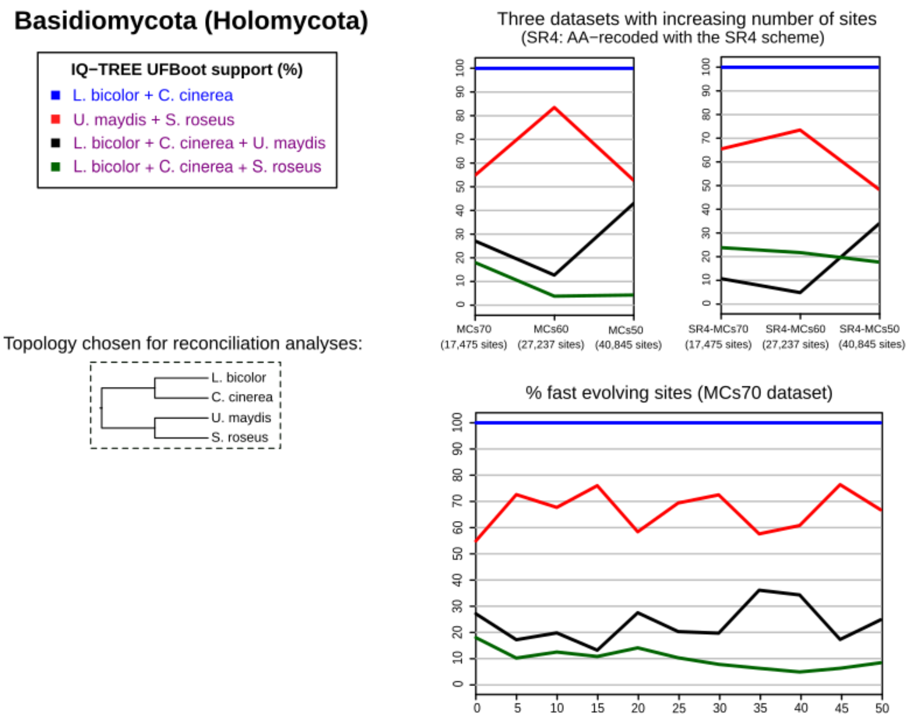

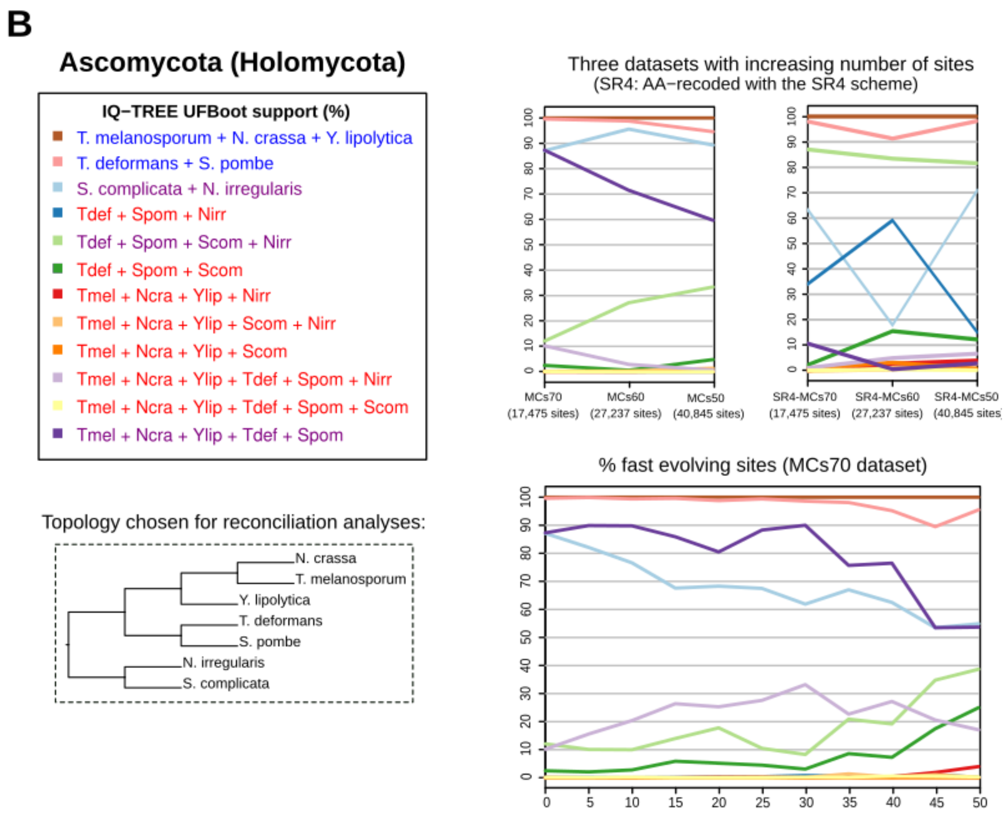

Supplementary Information 3-Fig. 6. Phylogenetic analyses done to evaluate the support for a series of potential topologies related to two conflicting scenarios within Ascomycota and Basidiomycota. Three distinct analyses were done. The first analysis consisted in measuring the UFBoot % support of all topologies evaluated under three datasets with distinct sizes. The second analysis similar to the first but using a recoded version of the MCs70, MCs60 and MCs50 datasets according to Susko and Rogers' four letter alphabet (SR-4). The third analyses consisted in measuring the UFBoot % support of all topologies under the progressive removal of the 5% of the fastest-evolving sites remaining in the supermatrix (see Methods). The topologies evaluated for every scenario correspond to those shown in the top left panels. Topologies colored in blue correspond to those that were confidently accepted after the analyses, in red those that were confidently rejected, and in purple those topologies that could not be neither confidently accepted nor rejected. The final topology chosen for each scenario is shown in the bottom panels.

Overall, these phylogenomic analyses were specifically designed to establish a reliable phylogenetic framework of Opisthokonta, as we particularly focused on this eukaryotic supergroup. However, the reconciliation analyses also incorporated representatives from other non-Opisthokonta lineages (all species included in the reconciliation analyses are highlighted in bold font in Supplementary Table 4). Because determining the phylogenetic relationships between that other lineages and Opisthokonta would have required a highly comprehensive eukaryotic dataset that would have remarkably increased the computational burden, these were directly determined from a consensus of currently available bibliographical references [11–19]. Regarding Opisthokonta taxa, species with only transcriptomic or poor quality genomic data were excluded from the reconciliation analyses (those labeled in grey in Supplementary Information 3-Fig. 1). See Supplementary Information 3-Fig. 7 for an ultrametric tree showing the species tree topology used in the reconciliation analyses.

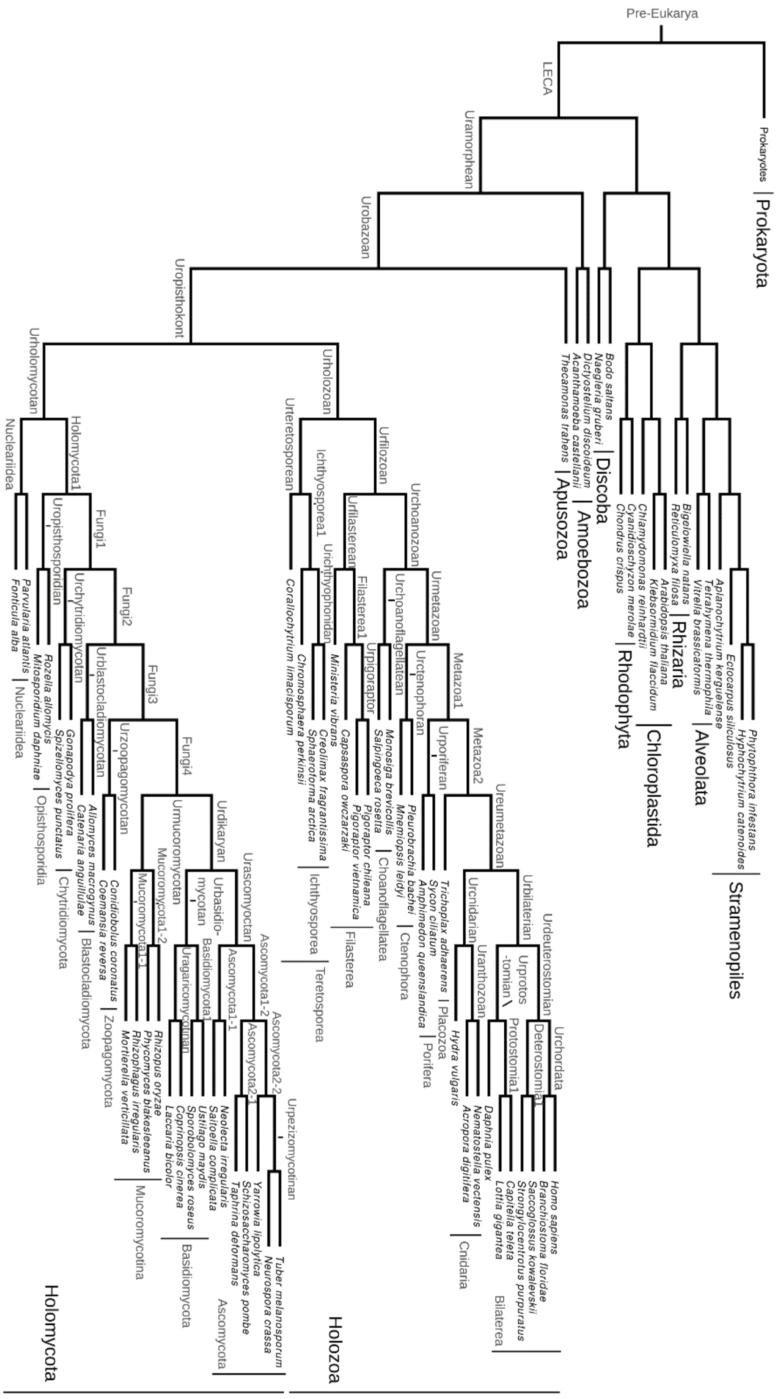

Supplementary Information 3-Fig. 7. Ultrametric species tree used for the gene tree species trees reconciliation analyses. Names given to the ancestral opisthokont lineages, used in Supplementary Tables, are indicated on top of the corresponding branches.

References

1. Susko, E. & Roger, A. J. On reduced amino acid alphabets for phylogenetic inference. *Mol. Biol. Evol.* **24**, 2139–2150 (2007).

2. Simion, P. *et al.* A Large and Consistent Phylogenomic Dataset Supports Sponges as the Sister

Group to All Other Animals. *Curr. Biol.* **27**, 958–967 (2017).

3. Pisani, D. *et al.* Genomic data do not support comb jellies as the sister group to all other animals. *Proc. Natl. Acad. Sci.* **112**, 15402–15407 (2015).

4. Laumer, C. E. *et al.* Revisiting metazoan phylogeny with genomic sampling of all phyla. *Proc. R. Soc. B Biol. Sci.* **286**, 20190831 (2019).

5. Laumer, C. E. *et al.* Support for a clade of Placozoa and Cnidaria in genes with minimal compositional bias. *Elife* **7**, e36278 (2018).

6. Whelan, N. V, Kocot, K. M., Moroz, L. L. & Halanych, K. M. Error, signal, and the placement of Ctenophora sister to all other animals. *Proc. Natl. Acad. Sci. U. S. A.* **112**, 5773–5778 (2015).

7. Hehenberger, E. *et al.* Novel Predators Reshape Holozoan Phylogeny and Reveal the Presence of a Two-Component Signaling System in the Ancestor of Animals. *Curr. Biol.* **27**, 2043–2050 (2017).

8. López-Escardó, D. *et al.* Reconstruction of protein domain evolution using single-cell amplified genomes of uncultured choanoflagellates sheds light on the origin of animals. *Philos. Trans. R. Soc. B Biol. Sci.* **374**, 20190088 (2019).

9. Torruella, G. *et al.* Global transcriptome analysis of the aphelid Paraphelidium tribonemae supports the phagotrophic origin of fungi. *Commun. Biol.* **1**, 231 (2018).

10. Ahrendt, S. R. *et al.* Leveraging single-cell genomics to expand the fungal tree of life. *Nat. Microbiol.* **3**, 1417–1428 (2018).

11. Brown, M. W. *et al.* Phylogenomics Places Orphan Protistan Lineages in a Novel Eukaryotic Super-Group. *Genome Biol. Evol.* **10**, 427–433 (2018).

12. Janouškovec, J. *et al.* A New Lineage of Eukaryotes Illuminates Early Mitochondrial Genome Reduction. *Curr. Biol.* **27**, 3717–3724 (2017).

13. Parfrey, L. W., Lahr, D. J. G., Knoll, A. H. & Katz, L. A. Estimating the timing of early eukaryotic diversification with multigene molecular clocks. *Proc. Natl. Acad. Sci.* **108**, 13624–13629 (2011).

14. Karnkowska, A. *et al.* A Eukaryote without a Mitochondrial Organelle. *Curr. Biol.* **26**, 1274–1284 (2016).

15. Derelle, R. *et al.* Bacterial proteins pinpoint a single eukaryotic root. *Proc. Natl. Acad. Sci. U. S. A.* **112**, E693–E699 (2015).

16. Derelle, R., López-García, P., Timpano, H. & Moreira, D. A phylogenomic framework to study the diversity and evolution of stramenopiles (=heterokonts). *Mol. Biol. Evol.* **33**, 2890–2898 (2016).

17. Strassert, J. F. H., Jamy, M., Mylnikov, A. P., Tikhonenkov, D. V. & Burki, F. New phylogenomic analysis of the enigmatic phylum Telonemia further resolves the eukaryote tree of life. *Mol. Biol. Evol.* **36**, 757–765 (2019).

18. Burki, F. *et al.* Untangling the early diversification of eukaryotes: a phylogenomic study of the evolutionary origins of Centrohelida, Haptophyta and Cryptista. *Proc. R. Soc. B Biol. Sci.* **283**, 20152802 (2016).

19. Betts, H. C. *et al.* Integrated genomic and fossil evidence illuminates life’s early evolution and eukaryote origin. *Nat. Ecol. Evol.* **2**, 1556–1562 (2018).
