# Supplementary material for: Divergent genomic trajectories predate the origin of animals and fungi
Source: Nature. 2022 Aug 24;609(7928):747–53. doi: 10.1038/s41586-022-05110-4 (PMC9492541; doi:10.1038/s41586-022-05110-4)
Supplement: Supplementary file 6 — Brief introduction to multicellularity and complex multicellularity in the context of the eukaryotic supergroup Opisthokonta with a series of analyses in relation to the origin of complex multicellularity in Fungi. [file 41586_2022_5110_MOESM6_ESM.pdf]

Supplementary Information 4

This supplementary information file includes a brief introduction to multicellularity and complex multicellularity in the context of the eukaryotic supergroup Opisthokonta. The final section includes a series of analyses in relation to the origin of complex multicellularity in Fungi.

Multicellularity and complex multicellularity

Multicellularity has been particularly successful in eukaryotes, not only because it would have independently evolved at least 16 times[1], but also because it is in this domain of life where it has reached the highest levels of biological complexity, particularly in five groups: animals, land plants, and some groups of brown algae, red algae, and fungi[2]. Besides cellular aggregation, which is relatively common in eukaryotes[1], the multicellularity of these five groups also includes molecular mechanisms involved in cell-to-cell communication and both spatial and temporal cell differentiation. For this reason, there is consensus in describing the multicellular phenotype of these groups as complex multicellularity (CM), which differentiates from simpler forms of multicellularity that are more prevalent in eukaryotes. Another common feature of complex multicellularity would be the existence of a three dimensional structure in which only a fraction of cells is exposed to the extracellular environment[2].

Multicellularity in Opisthokonta

Animals, land plants and multicellular fungi (mushrooms) are the most well studied groups among those that present CM. Opisthokonta thus represents an excellent phylogenetic framework where to study the origin of CM in eukaryotes, as it includes animals, fungi, and the protist relative lineages of both groups[3]. Given the widespread distribution of multicellularity in Opisthokonta (see below), it is tentative to suggest that the common ancestor of this eukaryotic supergroup had at least a clear potential to evolve multicellularity. However, there is no evidence to suggest that this ancestor could have presented CM. Firstly, because CM is completely different in Metazoa and Fungi from a cell biology perspective. Secondly, because CM is not observed neither in the protist lineages that branch as sister-group to Metazoa in the Holozoa division of Opisthokonta, nor in the the protist relative lineages of Fungi in the Holomycota division of Opisthokonta (see below).

The main difference between animals and the rest of Holozoa is the fact that all metazoans are multicellular. Animal multicellularity is characterized by a complex genetically regulated spatial and temporal cell differentiation program. CM was a remarkable innovation in the path to Metazoa[4], and indeed, most of the gene functional categories that expanded in the

metazoan root are tightly linked to multicellularity (see main text). However, besides Metazoa, simpler versions of multicellularity have been observed in some representatives of the other holozoan groups. Clonal multicellularity have been characterized in the choanoflagellate *Salpingoeca rosetta*[5], and it has recently been described a choanoflagellate species that presents an aggregative multicellular behavior[6]. Aggregative multicellularity has also been observed in the four known species of the group Filasterea. This includes the amoeboid species *Ministeria vibrans* and the flagellated species *Pigoraptor vietnamica* and *Pigoraptor chileana*[7] (for which we produced genomic data for this manuscript), although it has only been studied in the filasterean *Capsaspora owczarzaki*[8,9]. Finally, coenocytes are characteristic of some multinucleated stages in some members of the group Teretosporea[10,11].

Holomycota, the other division of Opisthokonta, also includes different forms of multicellularity. On the one hand, *Fonticula alba*, a poorly characterized species that branches as sister-group to the nucleariids (a group of amoeboid organisms that includes *Parvularia atlantis*, another species that was sequenced for this study). *F. alba* presents two distinct multicellular behaviors: a bacteria-feeding sorocarpic amoeba stage[12], and collective invasive behavior which has been recently described[13]. On the other hand, the most paradigmatic example of multicellularity in Holomycota is the fungal hypha. However, not all Fungi present hypha, and the presence of hypha is not *per se* a signature of CM (see the following section).

Complex multicellularity in Fungi

In contrast to animal clonal multicellularity, fungal multicellularity is based on chains of interconnected cells organized in branching filaments known as hyphae[14]. Despite hyphae being widespread across Fungi, CM (i.e., tissues with tridimensional organization, with molecular mechanisms for cell communication and spatial cell differentiation regulated by a developmental program) is restricted to only a few phylogenetically distant lineages[15]. The convoluted distribution of CM in Fungi complicates the inference of its origins, which could go from a single event and multiple losses to multiple independent origins[15]. Previous studies have reported mostly differences but also similarities in the molecular toolkits involved in CM across Fungi[16,17]. While the genetic similarities may favor the single origin hypothesis, they may also be explained by convergence on a molecular level (e.g., co-option and/or expansion of the same gene families in distinct CM lineages). Knowing whether these similarities were already present or not in the common ancestor of CM fungi would provide insights into this yet unsolved evolutionary history. We thus searched for signatures at gene content level differentiating CM fungi from other fungi using a manual and an unsupervised approach and explored the inferred gene contents of the corresponding ancestors for the hypothetical presence of these signatures (see below).

Despite being controversy on which fungal lineages should be considered to present CM[14–16], Agaricomycotina (*Coprinopsis cinerea* + *Laccaria bicolor*), Pezizomycotina (*Neurospora crassa* + *Tuber melanosporum*) and *Neolecta* (*Neolecta irregularis*) are *bona fide* cases of fungal CM in our dataset[15,16]. For the manual approach, we first searched for orthogroups (OGs) distributed only in CM taxa among Fungi (Supplementary Information 4-Fig. 1A). Of these, 71 OGs were found in at least 3 CM taxa (subset A in Supplementary Table 15). Second, we searched for OGs present also in other fungi but with more copies in CM taxa (Supplementary Information 4-Fig. 1B). Of these, 13 were found with more copies in at least 3 CM taxa (subset B in Supplementary Table 15). Only 9 and 3 OGs from subsets A and B, respectively, were common to at least 4 of the 5 CM taxa, and none was common to all of them. While these values are concordant with the overall poor homology between the CM molecular toolkits of the distinct CM lineages[15], our stringent conditions may have limited the detection of further OGs potentially related to CM origin/s in Fungi. For example, while a given ancestral innovation related to CM may have been secondarily lost in multiple yeast lineages due to a reversion to unicellularity, some could have retained and co-opted it for other purposes. Also, some of those OGs that experienced gene gains in CM fungi could have also been expanded in non-CM lineages. We thus used a less stringent and unsupervised approach based on the Random Forest (RF) classifier, which not only classifies data into categories (CM taxa vs other fungi) but also reports which features (in this case, which OGs) were the most informative for the classification purpose. In particular, RF decides on a consensus of multiple suboptimal decision trees, each of which has only access to a subset of the available features to prevent overfitting. We performed 5 independent runs of RF, as each round is blind to a distinct subset of the data. There were 14 OGs used by at least 3 of the 5 RF runs. Of these, 4 and 1, respectively, coincide with the subsets A and B, and 13 have more copy numbers in CM fungi than in non-CM fungi. These 13 OGs (see subset C in Supplementary Table 15) are thus good candidates for being used as markers to explore the evolutionary history of CM in Fungi.

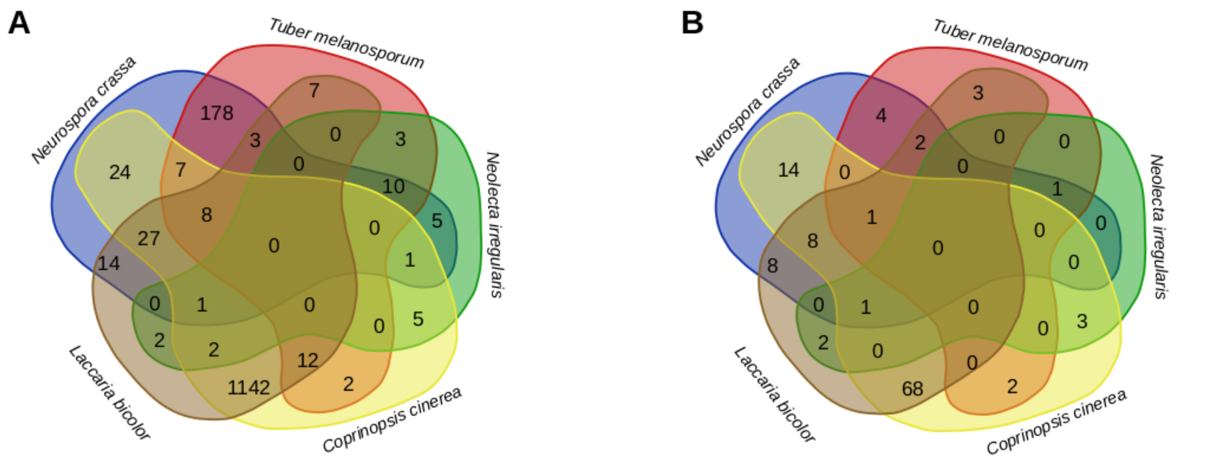

Supplementary Information 4-Fig. 1. (**A**) Taxonomic distribution of those OGs present in > 1 CM fungi but not in non-CM fungi. For example, the number 8 in the intersection between *N. crassa*, *T. melanosporum*, *L. bicolor* and *C. cinerea* indicates that there are eight OGs which are present in these four CM species despite being absent from the rest of the sampled fungi. (**B**) Distribution of those OGs presenting more copy numbers in at least two CM fungi than in any other fungi. For example, the number 68 in the intersection between *L. bicolor* and *C. cinerea* indicates that there are 68 OGs which show more copy numbers specifically in these two CM species than in any non-CM fungi. Note that the values are higher at this particular intersection (*L. bicolor* + *C. cinerea*) because both species are more phylogenetically related between them than any other pair of CM fungi.

On the whole, the low number of OGs that compose the three subsets agree with previous studies which have reported poor homology at genetic level in relation to CM in Fungi[15]. This could be explained either because CM would have evolved independently in each CM group, or because a given hypothetical ancestral toolkit for CM would have been replaced at least to a great extent by group-specific genetic innovations. If the second scenario were true, we would expect to find that the 13 OGs from subset C, which are potential components of an ancestral toolkit for CM (provided that it ever existed), would not have expanded independently in the distinct CM groups but rather along the ancestral path that is common to all them.

Our results partially agree with each of the two scenarios proposed above. On the one hand, the highest copy number for these 13 OGs is found in Pezizomycotina (Ascomycota) and in Agaricomycotina (Basidiomycota), which in our dataset are represented by *N. crassa* and *T. melanosporum* and by *C. cinerea* and *L. bicolor*, respectively (Supplementary Information 4-Fig. 2B). Given that both groups present the most complex multicellularity among Fungi, our results confirm the occurrence of genetic changes accompanying this phenotypic complexification. However, we observe that the increase in copy number for these OGs predated the emergence of both groups, and indeed started not in their last common ancestor (Dikarya), but in the preceding ancestral node (Dikarya + Mucoromycotina)..

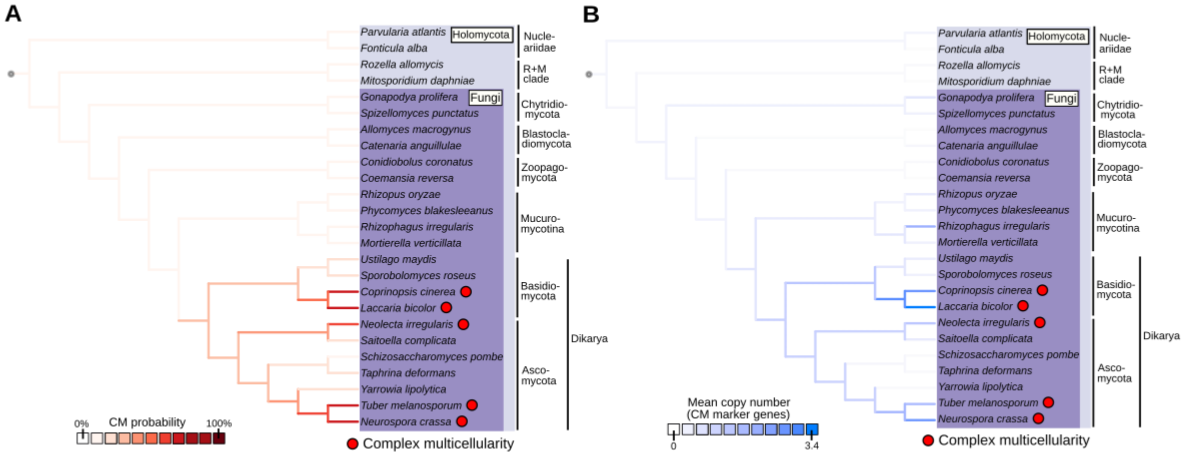

Supplementary Information 4-Fig. 2. Phylogeny of Holomycota, with branches being colored according to (**A**) the probabilities retrieved from the Random Forest classifier trained to detect CM-Fungi based on the relative OG representation of every genome (excluding species-specific OGs) and (**B**) mean counts for the 13 OGs used by more than half of the runs of the classifier (see text).

Although the canonical cases of fungal CM are in Dikarya, fruiting bodies -i.e., fungal organs with CM patterns- have also been described in Mucoromycotina[15]. Within Dikarya, these OGs are mostly absent from non-CM species, most of which evolved a yeast phenotype. The absence of these OGs from these species is not surprising given that the classifier was trained to find potential markers of CM in Fungi (i.e., OGs present in CM fungi and absent in non-CM fungi). However, for this same reason, it is remarkable that some of the best markers of CM found by the classifier (the 13 OGs from subset C) are also distributed across Mucoromycotina, in spite of the fact that the species from this group were not considered as CM fungi during the training step of the classifier. Based on this finding, we suggest that future studies exploring the hypothesis of a single origin for CM in Fungi should focus not only on those evolutionary changes occurred at the root of Dikarya, but also on those occurred at the preceding ancestral node. .

Overall, given the ancestral signal detected (Supplementary Information 4-Fig. 2B), it is tentative to hypothesize that CM could have already been present at the root of Dikarya + Mucoromycotina, or at least a rudimentary version of this phenotype. CM would have later become more complex in some groups of Dikarya. However, an alternative scenario, proposed by [15], is that fungal CM is the outcome of an outstanding process of convergent evolution that could have been favored by the possibility of co-opting ancestral genes that were appropriate to evolve this phenotype [15]. Both scenarios are compatible with the probabilities retrieved by the classifier, which are minor but still not null in the deeper nodes of Dikarya and show a substantial increment in Agaricomycotina and Pezizomycotina (Supplementary Information 4-Fig. 2A).

Bibliography

1. Sebé-Pedrós, A., Degnan, B. M. & Ruiz-Trillo, I. The origin of Metazoa, a unicellular perspective. *Nat. Rev. Genet.* **18**, 498–512 (2017).
2. Knoll, A. H. The Multiple Origins of Complex Multicellularity. *Annu. Rev. Earth Planet. Sci.* **39**, 217–239 (2011).
3. Torruella, G., de Mendoza, A., Grau-Bové, X. & Ruiz-Trillo, I. Phylogenomics Reveals Convergent Evolution of Lifestyles in Close Relatives of Animals and Fungi. *Curr. Biol.* **25**, 2404–2410 (2015).
4. Ros-Rocher, N., Pérez-Posada, A. & Leger, M. M. The origin of animals: an ancestral reconstruction of the unicellular-to- multicellular transition. *Open Biol.* **11**, 200359 (2021).
5. Dayel, M. J. *et al.* Cell differentiation and morphogenesis in the colony-forming choanoflagellate Salpingoeca rosetta. *Dev. Biol.* **357**, 73–82 (2011).
6. Brunet, T. *et al.* Light-regulated collective contractility in a multicellular choanoflagellate. *Science.* **366**, 326–334 (2019).
7. Tikhonenkov, D. V. *et al.* Insights into the origin of metazoan multicellularity from predatory unicellular relatives of animals. *BMC Biol.* **18**, 39 (2020).
8. Ferrer-Bonet, M. & Ruiz-Trillo, I. Capsaspora owczarzaki. *Current Biology* **27**, R829–R830 (2017).
9. Sebé-Pedrós, A. *et al.* Regulated aggregative multicellularity in a close unicellular relative of metazoa. *Elife* **2**, e01287 (2013).
10. Ondracka, A., Dudin, O. & Ruiz-Trillo, I. Decoupling of Nuclear Division Cycles and Cell Size during the Coenocytic Growth of the Ichthyosporean Sphaeroforma arctica. *Curr. Biol.* **28**, 1964-1969.e2 (2018).
11. Kożyczkowska, A. *et al.* Stable transfection in protist Corallochytrium limacisporum identifies novel cellular features among unicellular animals relatives. *Curr. Biol.* **31**, 4104-4110.e5 (2021).
12. Worley, A. C., Raper, K. B. & Hohl, M. Fonticula alba: A New Cellular Slime Mold (Acrasiomycetes). *Mycologia* **71**, 760 (1979).
13. Toret, C., Picco, A., Boiero-Sanders, M., Michelot, A. & Kaksonen, M. The cellular slime mold Fonticula alba forms a dynamic, multicellular collective while feeding on bacteria. *Curr. Biol.* **32**, 1–13 (2022).
14. Kües, U., Khonsuntia, W. & Subba, S. Complex fungi. *Fungal Biol. Rev.* **32**, 205–218 (2018).
15. Nagy, L. G., Kovács, G. M. & Krizsán, K. Complex multicellularity in fungi: evolutionary convergence, single origin, or both? *Biol. Rev.* **93**, 1778–1794 (2018).
16. Nguyen, T. A. *et al.* Innovation and constraint leading to complex multicellularity in the Ascomycota. *Nat. Commun.* **8**, 14444 (2017).
17. Krizsán, K. *et al.* Transcriptomic atlas of mushroom development reveals conserved genes behind complex multicellularity in fungi. *Proc. Natl. Acad. Sci. U. S. A.* **116**, 7409–7418 (2019).
